# Regioselective S_N_2' Mitsunobu reaction of Morita–Baylis–Hillman alcohols: A facile and stereoselective synthesis of α-alkylidene-β-hydrazino acid derivatives

**DOI:** 10.3762/bjoc.10.98

**Published:** 2014-04-30

**Authors:** Silong Xu, Jian Shang, Junjie Zhang, Yuhai Tang

**Affiliations:** 1 Department of Chemistry, School of Science, Xi’an Jiaotong University, Xi’an 710049, P. R. China

**Keywords:** azodicarboxylate, hydrazine, Mitsunobu reaction, Morita–Baylis–Hillman, S_N_2' reaction

## Abstract

A highly regioselective S_N_2' Mitsunobu reaction between Morita–Baylis–Hillman (MBH) alcohols, azodicarboxylates, and triphenylphosphine is developed, which provides an easy access to α-alkylidene-β-hydrazino acid derivatives in high yields and good stereoselectivity. This reaction represents the first direct transformation of MBH alcohols into hydrazines.

## Introduction

Hydrazines and their derivatives are an important class of compounds in organic chemistry. They are widely used in the fields of pesticides, polymers, dyestuff, and pharmaceutical agents [1]. They are also versatile building blocks for accessing many important nitrogen-containing heterocyclic compounds, especially pyrazole derivatives [2–7]. Although various methods detailing the synthesis of hydrazines have been established [8], the development of an efficient synthesis of hydrazines with highly structural diversity from simple starting materials under mild conditions is still desirable.

Morita–Baylis–Hillman (MBH) adducts [9] are a class of unique substrates of great synthetic potential which contain three manipulatable groups, namely, a hydroxy group, a carbon–carbon double bond, and an electron-withdrawing group. Over the past several decades, a myriad of transformations involving MBH adducts have been reported, leading to a wide variety of molecular scaffolds of high diversity and complexity [10–12]. A number of reports have dealt with the conversion of the hydroxy group of MBH adducts into useful functionalities, such as halides [13–14], ethers [15–16], amines [17–18], thioethers [19], phosphonates [20–21], alkyl groups [22–23], and so on. In this context, however, reports on the conversion of MBH alcohols into hydrazine derivatives are scanty.

**Scheme 1:**
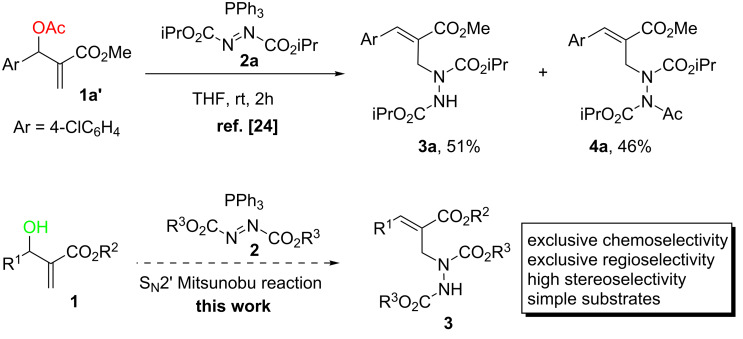
Synthesis of α-alkylidene-β-hydrazino acid derivatives from MBH adducts.

In 2009, Nair and co-workers [24] reported an interesting reaction of MBH acetates with azodicarboxylates in the presence of PPh_3_ (Mitsunobu reaction conditions), which gives an efficient access to α-alkylidene-β-hydrazino acid derivatives, an important precursor for many bioactive compounds [25–30] including β-amino acids [25] (Scheme 1, top). However, the reaction exhibited poor chemoselectivity which gave comparable yields of tri- and tetrasubstituted hydrazines. For example, the reaction of MBH acetate **1a**' with diisopropyl azodicarboxylate (**2a**) and PPh_3_ afforded hydrazines **3a** and **4a** in 51% and 46% yields, respectively. Inspired by this report, and a pioneering S_N_2' Mitsunobu reaction of MBH alcohols with carboxylic acids [31–32], we envisioned that direct treatment of simpler MBH alcohols with azodicarboxylates and PPh_3_ (Mitsunobu conditions) would chemoselectively provide trisubstituted hydrazines of type **3**, via a distinct S_N_2' Mitsunobu reaction approach (Scheme 1, bottom). As part of our interest in exploring new reactivities of MBH adducts [33–34], herein we wish to report the preliminary results from such an investigation.

## Results and Discussion

**Scheme 2:**
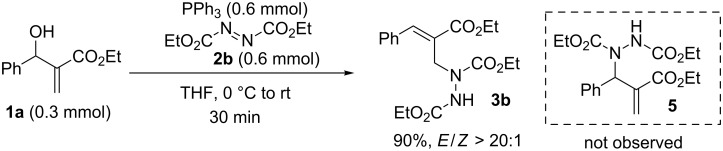
Initial investigation.

In our initial study, MBH alcohol **1a** was treated with 2.0 equivalents of diethyl azodicarboxylate (**2b**) and triphenylphosphine under very mild conditions (Scheme 2). To our delight, the reaction was completed in 30 minutes providing the anticipated trisubstituted hydrazine **3b** in 90% isolated yield with excellent *E*-selectivity (*E*/*Z* > 20:1). To the best of our knowledge, this reaction represents the first direct conversion of MBH alcohols into hydrazines. In addition, the normal S_N_2 Mitsunobu reaction [35–36] product **5**, namely, diethyl 1-(2-(ethoxycarbonyl)-1-phenylallyl)hydrazine-1,2-dicarboxylate, could not be detected in the reaction mixture, which suggested a highly regioselective S_N_2' Mitsunobu process occurred in the reaction (see discussion on mechanism below).

With the encouraging result, the reaction conditions were further optimized using the above reaction as a model (Table 1). Among several common solvents screened, dichloromethane, chloroform, and toluene gave comparable yields to that of THF, while ethyl acetate emerged as the best solvent, offering an excellent 98% yield (Table 1, entries 2–5). However, polar solvents such as DMF, ethanol, and acetonitrile were detrimental to the reaction, giving very low yields (Table 1, entries 6–8). Reducing the amounts of **2b** and PPh_3_ from 2.0 equivalents to 1.5 or 1.0 equivalent resulted in substantial decrease in the yields (Table 1, entries 9 and 10). It was also found that replacing PPh_3_ with more nucleophilic PBu_3_ in the reaction could shorten the reaction time but led to an inferior yield (Table 1, entry 11). Therefore PPh_3_ was chosen as the preferable phosphine due to its high efficiency and cost-effectiveness.

**Table 1:** Investigation on reaction conditions.^a^

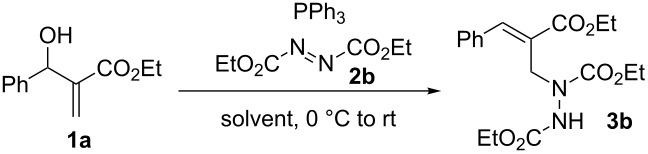			

Entry	Solvent	Time (min)	Yield^b^ (%)

1	THF	30	90
2	CH_2_Cl_2_	20	80
3	CHCl_3_	40	86
4	toluene	35	88
5	EtOAc	25	98
6	DMF	24	trace
7	EtOH	50	23
8	CH_3_CN	30	39
9^c^	EtOAc	25	29
10^d^	EtOAc	25	54
11^e^	EtOAc	10	82

^a^Diethyl azodicarboxylate (**2b**, 0.6 mmol) was slowly added to a solution of MBH alcohol **1a** (0.3 mmol) and PPh_3_ (0.6 mmol) in the specified solvent (2 mL) at 0 °C under N_2_, and then the mixture was allowed to warm up to room temperature. ^b^Isolated yield. ^c^The reaction was conducted using 1.0 equiv of both PPh_3_ and **2b**. ^d^1.5 equiv of PPh_3_ and **2b** were adopted. ^e^PBu_3_ was used instead of PPh_3_.

Under the optimized conditions, the scope of the S_N_2' Mitsunobu reaction was examined (Table 2). Variation in the ester alkyl groups of both MBH alcohols and azodicarboxylates had little influence on the reaction; the corresponding hydrazines with different ester groups were generated in excellent yields and stereoselectivity (Table 2, entries 1–3). In addition, a range of aromatic MBH alcohols **1c–g** featuring either an electron-donating or an electron-withdrawing group on the benzene ring all worked well, delivering the hydrazines **3e–i** in high yields (79–91%) and excellent *E*/*Z* selectivity, with an exception of the nitro-substituted **1g** giving a moderate *E*/*Z* ratio (Table 2, entries 4–8). An *ortho* substituent on the benzene ring of the MBH alcohol was also well tolerated (Table 2, entry 5 vs 6). Notably, in contrast with Nair’s report [24], it was revealed that aliphatic MBH alcohols were excellent candidates in the reaction. By switching the solvent to dichloromethane, several alkyl MBH alcohols containing ethyl, methyl, or hydrogen substituents (**1h–l**) readily reacted with representative azodicarboxylates, producing the corresponding α-alkylidene-β-hydrazino esters **3** in high yields (80–90%) and good *E*/*Z* selectivity (Table 2, entries 9–18). An exception was observed for the reaction of MBH alcohol **1j** with *tert*-butyl azodicarboxylate (**2c**), which gave 55% yield and a modest *E*/*Z* ratio (5:1) (Table 2, entry 14).

**Table 2:** Synthesis of α-alkylidene-β-hydrazino esters **3**.^a^

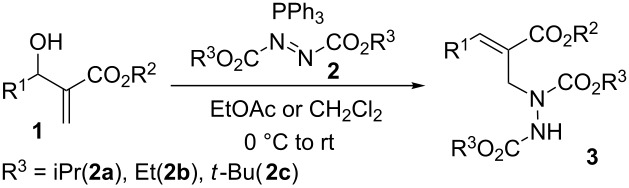					

Entry	R^1^, R^2^ in **1**	**2**	Time (min)	**3**, Yield^b^ (%)	*E*/*Z*^c^

1	C_6_H_5_, Et (**1a**)	**2b**	25	**3b**, 98	20:1
2	C_6_H_5_, Me (**1b**)	**2b**	25	**3c**, 95	20:1
3	C_6_H_5_, Me (**1b**)	**2c**	50	**3d**, 95	20:1
4	4-CH_3_C_6_H_4_, Et (**1c**)	**2b**	35	**3e**, 81	20:1
5	2-ClC_6_H_4_, Et (**1d**)	**2b**	35	**3f**, 87	20:1
6	4-ClC_6_H_4_, Et (**1e**)	**2b**	30	**3g**, 91	20:1
7	4-FC_6_H_4_, Me (**1f**)	**2b**	30	**3h**, 79	20:1
8	4-NO_2_C_6_H_4_, Et (**1g**)	**2c**	40	**3i**, 86	3:1
9	C_2_H_5_, Et (**1h**)	**2a**	30	**3j**, 80	9:1
10	C_2_H_5_, Et (**1h**)	**2b**	20	**3k**, 82	20:1
11	C_2_H_5_, Me (**1i**)	**2b**	25	**3l**, 80	20:1
12	C_2_H_5_, Me (**1i**)	**2a**	30	**3m**, 81	13:1
13	CH_3_, Et (**1j**)	**2b**	21	**3n**, 83	13:1
14	CH_3_, Et (**1j**)	**2c**	40	**3o**, 55	5:1
15	H, Et (**1k**)	**2b**	10	**3p**, 90	–
16	H, Et (**1k**)	**2a**	20	**3q**, 89	–
17	H, Et (**1k**)	**2c**	25	**3r**, 90	–
18	H, Me (**1l**)	**2b**	13	**3s**, 87	–

^a^Under N_2_ atmosphere and at 0 °C, to a solution of MBH alcohol **1** (0.3 mmol) and PPh_3_ (0.6 mmol) in 2 mL ethyl acetate (for entries 9–18, dichloromethane was used as the solvent) was slowly added azodicarboxylates **2** (0.6 mmol), and the mixture was stirred at room temperature and monitored by TLC. ^b^Isolated yield. ^c^Determined by ^1^H NMR assay.

**Scheme 3:**
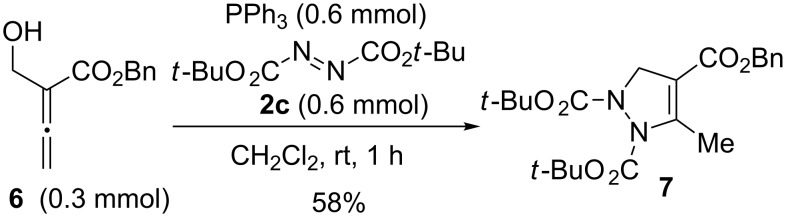
Synthesis of 1*H*-pyrazole **7**.

To further investigate the scope, a single case using allenic MBH alcohol **6** as the substrate was studied (Scheme 3). However, under similar conditions, the reaction between alcohol **6**, *tert*-butyl azodicarboxylate (**2c**), and PPh_3_ afforded an interesting 1*H*-pyrazole compound **7** in 58% yield. A possible mechanism for the formation of **7** is outlined in Supporting Information File 1.

The above results clearly show that the S_N_2' Mitsunobu reaction between MBH alcohols, azodicarboxylates, and PPh_3_ has a broad substrate scope. The reaction is clean and fast, with all the reactions completed in less than one hour. The reaction exhibits good stereoselectivity (*E*/*Z* 3:1 to 20:1) and exclusive regioselectivity. In all cases, the normal S_N_2 Mitsunobu products of type **5** could not be detected by using ^1^H and ^13^C DEPT NMR analysis. The structure and stereochemistry of all hydrazines **3** were well identified by ^1^H, ^13^C NMR, IR, HRMS, and NOESY analysis for a representative product **3b** (for characterization data, see Supporting Information File 1).

**Scheme 4:**
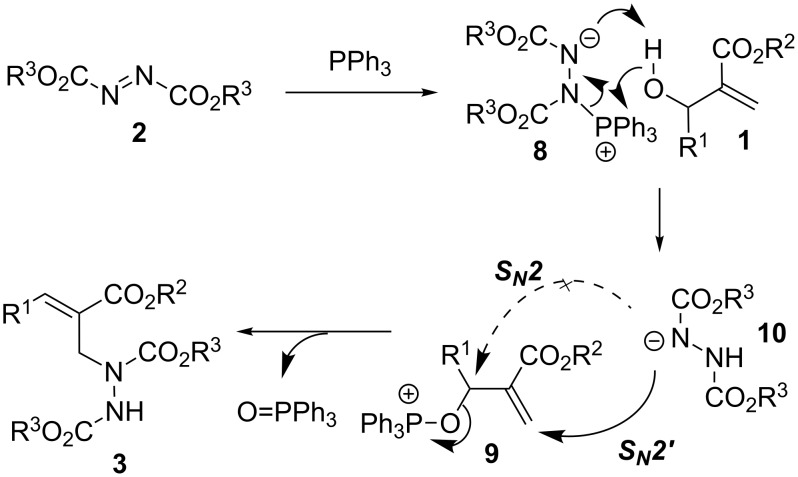
A plausible mechanism for the formations of **3**.

A possible mechanism for the S_N_2' Mitsunobu reaction between MBH alcohols **1**, azodicarboxylates **2**, and PPh_3_ is depicted in Scheme 4. Initially, the addition of PPh_3_ to azodicarboxylates **2** generates the Huisgen zwitterion intermediate **8** [37–38]. Subsequent proton transfer and phosphonium migration between **8** and the MBH alcohol **1** produce an oxophosphonium intermediate **9** and a hydrazine anionic species **10** [36]. Finally, an expedient S_N_2' attack of species **10** on **9**, probably facilitated by the ester group of **9** [31–32], leads to the formation of hydrazines **3** and phosphine oxide. The alternative S_N_2 displacement of the oxophosphonium moiety of **9** by the species **10** may be retarded by steric hindrance. Recently, S_N_2' Mitsunobu reactions [31–32,39–45] have attracted considerable interest from the organic chemistry community due to their great synthetic potential being complementary to the Mitsunobu reactions. This report accordingly adds to a new valuable example of S_N_2' Mitsunobu reactions.

## Conclusion

In conclusion, we have developed a highly regioselective S_N_2' Mitsunobu reaction between Morita–Baylis–Hillman (MBH) alcohols, azodicarboxylates, and triphenylphosphine as an efficient synthetic method for α-alkylidene-β-hydrazino acid derivatives in high yields and good stereoselectivity. This reaction features additional advantages as mild conditions, wide substrate scope, and simple starting materials. The reaction represents the first direct transformation of MBH alcohols into hydrazines, and constitutes a valuable example of regioselective S_N_2' Mitsunobu reactions. Our future efforts will focus on the application of the current reaction in the synthesis of nitrogen-containing heterocyclic compounds.

## Experimental

### General information

All reactions were carried out in nitrogen atmosphere under anhydrous conditions. Solvents were purified according to standard procedures. Mortia–Baylis–Hillman (MBH) alcohol **1** were prepared according to literature procedures [46–47]. Benzyl 2-(hydroxymethyl)buta-2,3-dienoate (**6**) is a known compound and was prepared according to a literature [48]. Reagents from commercial sources were used without further purification. ^1^H and ^13^C NMR spectra were recorded on a Bruker AV 400 spectrometer in CDCl_3_ with tetramethylsilane (TMS) as the internal standard. High resolution ESI mass spectra were acquired with an IonSpec QFT-ESI instrument. IR spectra were recorded on a Nicolet Avatar 330 FTIR spectrometer (in KBr). Column chromatography was performed on silica gel (200–300 mesh) using a mixture of petroleum ether (bp 60–90 °C)/ethyl acetate as the eluant.

### General procedure for the synthesis of hydrazines **3**

Under N_2_ atmosphere and at 0 °C, to a stirred solution of MBH alcohols **1** (0.3 mmol) and PPh_3_ (0.6 mmol) in EtOAc or CH_2_Cl_2_ (2 mL) in a Schlenk tube (25 mL) was slowly added azodicarboxylates **2** (0.6 mmol) over 5 minutes by the means of a microsyringe. The resulting reaction mixture was allowed to warm up to room temperature and stirred until the MBH alcohols **1** were completely consumed, as monitored by TLC. The solvent was removed under reduced pressure and the residue was purified by column chromatography on silica gel (gradient eluant: petroleum ether/ethyl acetate 9:1–3:1) to afford the hydrazines **3**.

### Synthesis of 1*H*-pyrazole **7**

Under N_2_ atmosphere and at room temperature, PPh_3_ (157 mg, 0.6 mmol) was slowly added to a stirred solution of allenic MBH alcohol **6** (61 mg, 0.3 mmol) and di-*tert*-butyl azodicarboxylate **2c** (138 mg, 0.6 mmol) in CH_2_Cl_2_ (2 mL) in a Schlenk tube (25 mL). The resulting reaction mixture was stirred for 1 hour at that time the alcohol **6** was disappeared as monitored by TLC. The solvent was removed using a rotatory evaporator under reduced pressure. The residue was then directly subject to column chromatography on silica gel (eluant: petroleum ether/ethyl acetate 9:1) to afford the 1*H*-pyrazole compound **7** in 73 mg, 58% yield, as slightly yellow oil.

## Supporting Information

File 1 Experimental details on the synthesis of all hydrazines **3** and 1*H*-pyrazole **7**, full characterization data and ^1^H, ^13^C, DEPT NMR spectra for all compounds **3** and **7**, and a mechanistic rationale for the formation of **7**.
