# Supplementary material for: Regioselective SN2' Mitsunobu reaction of Morita–Baylis–Hillman alcohols: A facile and stereoselective synthesis of α-alkylidene-β-hydrazino acid derivatives
Source: Beilstein J Org Chem. 2014 Apr 30;10:990–5. doi: 10.3762/bjoc.10.98 (PMC4077529; doi:10.3762/bjoc.10.98)
Supplement: File 1 — Experimental details on the synthesis of all hydrazines 3 and 1H-pyrazole 7, full characterization data and 1H, 13C, DEPT NMR spectra for all compounds 3 and 7, and a mechanistic rationale for the formation of 7. [file Beilstein_J_Org_Chem-10-990-s001.pdf]

Supporting Information

for

Regioselective S_N2' Mitsunobu reaction of Morita–Baylis–Hillman alcohols: A facile and stereoselective synthesis of α-alkylidene-β-hydrazino acid derivatives

Silong Xu*, Jian Shang, Junjie Zhang and Yuhai Tang*

Address: Department of Chemistry, School of Science, Xi'an Jiaotong University, Xi'an 710049, P. R. China

Email: Silong Xu - silongxu@mail.xjtu.edu.cn; Yuhai Tang - tyh57@mail.xjtu.edu.cn

* Corresponding author

Experimental details on the synthesis of all hydrazines 3 and 1*H*-pyrazole 7, full characterization data and ^{1}H, ^{13}C, DEPT NMR spectra for all compounds 3 and 7, and a mechanistic rationale for the formation of 7.

Table of contents

1. Analytical data for compounds **3b–s** ······ S2
2. Analytical data for 1*H*-pyrazole **7** and mechanistic rationale ······ S11
3. Copies of ^{1}H, ^{13}C, DEPT, and NOESY NMR spectra ······ S13

1. Analytical data for compounds 3b–s[1]

Table 2, entry 1. Following the general procedure, the reaction of MBH alcohol **1a** (62 mg, 0.3 mmol), PPh_3 (157 mg, 0.6 mmol) and diethyl azodicarboxylate **2b** (94 μL, 0.6 mmol) was performed for 25 minutes.

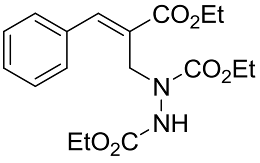

3b

3b: 107 mg, 98% yield, as slightly yellow oil; ^{1}H NMR (400 MHz, $CDCl_3$) δ 7.89 (s, H), 7.37 (m, 5H), 6.75 (br m, 1H), 4.64 (s, 2H), 4.27 (q, J = 7.1 Hz, 2H), 4.11 (m, 4H), 1.33 (t, J = 7.1 Hz, 3H), 1.22 (m, 6H); ^{13}C NMR (100 MHz, $CDCl_3$) δ 167.6, 156.0(2C), 143.7, 134.5, 129.3, 128.9, 128.5, 127.3, 62.5, 61.9, 61.1, 46.6, 14.41, 14.37, 14.2; IR (KBr): υ_{max} 3433, 2983, 1638, 1401, 1124 cm^{-1}; HRMS (ESI) calcd for $C_{18}H_{25}N_2O_6$ $[M+H]^+$ requires 365.1713, found 365.1712.

Table 2, entry 2. Following the general procedure, the reaction of MBH alcohol **1b** (58 mg, 0.3 mmol), PPh_3 (157 mg, 0.6 mmol) and diethyl azodicarboxylate **2b** (94 μL, 0.6 mmol) was performed for 25 minutes.

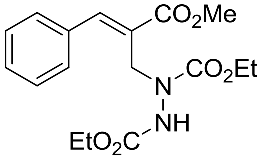

3c

3c: 100 mg, 95% yield, as yellow oil; ^{1}H NMR (400 MHz, $CDCl_3$) δ7.90 (s, 1H), 7.36 (m, 5H), 6.61 (br m, 1H), 4.65 (s, 2H), 4.18 (m, 4H), 3.83 (s, 3H), 1.29 (m, 6H); ^{13}C NMR (100 MHz, $CDCl_3$) δ168.0, 155.9(2C), 143.9, 134.3, 129.2, 128.9, 128.4, 127.0, 62.4, 61.7, 52.1, 46.5, 14.3(2C); IR (KBr): υ_{max} 3430, 2980, 1711, 1401, 1125 cm^{-1}; HRMS (ESI) calcd for $C_{17}H_{23}N_2O_6$ $[M+H]^+$ requires 351.1556, found 351.1557.

[1] Rotamers were often observed in the ^{1}H and ^{13}C NMR spectra of compounds **3**.

Table 2, entry 3. Following the general procedure, the reaction of MBH alcohol **1b** (58 mg, 0.3 mmol), PPh_3 (157 mg, 0.6 mmol) and di-*tert*-butyl azodicarboxylate **2c** (138 mg, 0.6 mmol) was performed for 50 minutes.

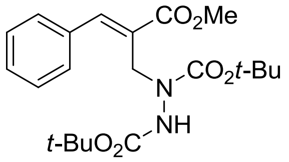

3d

3d: 116 mg, 95% yield, as colorless oil; ^{1}H NMR (400 MHz, $CDCl_3$) δ 7.77 (s, 1H), 7.28 (m, 5H), 6.32 (br m, 1H), 4.53 (s, 2H), 3.72 (s, 3H), 1.35 (m, 18H); ^{13}C NMR (100 MHz, $CDCl_3$) δ 168.0, 155.7, 154.9, 143.5, 134.3, 129.3, 128.8, 128.3, 127.2, 80.9, 80.6, 51.9, 45.1, 27.9(2C); IR (KBr): υ_{max} 3435, 2981, 1639, 1401, 1122 cm^{-1}; HRMS (ESI) calcd for $C_{21}H_{30}N_2NaO_6$ $[M+Na]^+$ requires 429.2002, found 429.1995.

Table 2, entry 4. Following the general procedure, the reaction of MBH alcohol **1c** (66 mg, 0.3 mmol), PPh_3 (157 mg, 0.6 mmol) and diethyl azodicarboxylate **2b** (94 μL, 0.6 mmol) was performed for 35 minutes.

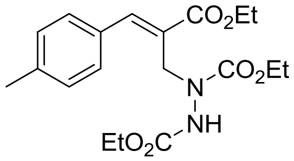

3e

3e: 92 mg, 81% yield, as yellow oil; ^{1}H NMR (400 MHz, $CDCl_3$) δ7.77 (s, 1H), 7.20 (br s, 2H), 7.08 (d, *J* = 7.9 Hz, 2H), 6.78 (br m, 1H), 4.56 (s, 2H), 4.16 (q, *J* = 7.1 Hz, 2H), 4.03 (m, 4H), 2.25 (s, 3H), 1.23 (t, *J* = 7.1 Hz, 3H), 1.11 (m, 6H); ^{13}C NMR (100 MHz, $CDCl_3$) δ 167.5, 155.8(2C), 143.7, 138.9, 131.4, 129.3, 129.0, 126.1, 62.1, 61.5, 60.8, 46.0, 21.0, 14.2, 14.1, 13.9; IR (KBr): υ_{max} 3413, 2981, 1710, 1401, 1124 cm^{-1}; HRMS (ESI) calcd for $C_{19}H_{27}N_2O_6$ $[M+H]^+$ requires 379.1869, found 379.1867.

Table 2, entry 5. Following the general procedure, the reaction of MBH alcohol **1d** (72 mg, 0.3 mmol), PPh_3 (157 mg, 0.6 mmol) and diethyl azodicarboxylate **2b** (94 μL, 0.6 mmol) was performed for 35 minutes.

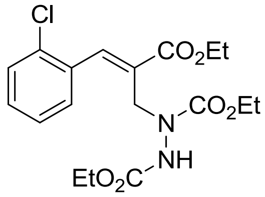

3f

3f: 104 mg, 87 % yield, as slightly yellow oil; ^{1}H NMR (400 MHz, $CDCl_3$) δ 7.94 (s, 1H), 7.33 (m, 4H), 6.61 (br m, 1H), 4.52 (s, 2H), 4.30 (q, J = 7.1 Hz, 2H), 4.11 (m, 4H), 1.35 (t, J = 7.2 Hz, 3H), 1.23 (m, 6H); ^{13}C NMR (100 MHz, $CDCl_3$) δ 166.9, 155.9(2C), 140.9, 133.7, 133.2, 130.3, 129.9, 129.4(2C), 126.6, 62.4, 61.8, 61.2, 46.8, 14.3(2C), 14.1; IR (KBr): υ_{max} 3422, 2985, 1717, 1401, 1136cm^{-1}; HRMS (ESI) calcd for $C_{18}H_{24}ClN_2O_6$ $[M+H]^+$ requires 399.1323, found 399.1324.

Table 2, entry 6. Following the general procedure, the reaction of MBH alcohol **1e** (72 mg, 0.3 mmol), PPh_3 (157 mg, 0.6 mmol) and diethyl azodicarboxylate **2b** (94 μL, 0.6 mmol) was performed for 30 minutes.

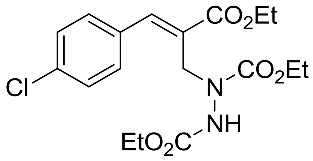

3g

3g: 109 mg, 91% yield, as colorless oil; ^{1}H NMR (400 MHz, $CDCl_3$) δ 7.82 (s, 1H), 7.35 (m, 4H), 6.92 (br m, 1H), 4.61 (s, 2H), 4.27 (q, J = 7.1 Hz, 2H), 4.13 (m, 4H), 1.33 (t, J = 7.1 Hz, 3H), 1.21 (m, 6H); ^{13}C NMR (100 MHz, $CDCl_3$) δ 167.1, 155.8(2C), 142.2, 134.8, 132.9, 130.5, 128.6, 127.9, 62.3, 61.7, 61.0, 46.1, 14.3, 14.2, 14.0; IR (KBr): υ_{max} 3448, 2985, 1712, 1401, 1124 cm^{-1}; HRMS (ESI) calcd for $C_{18}H_{24}ClN_2O_6$ $[M+H]^+$ requires 399.1323, found 399.1320.

Table 2, entry 7. Following the general procedure, the reaction of MBH alcohol **1f** (63 mg, 0.3 mmol), PPh_3 (157 mg, 0.6 mmol) and diethyl azodicarboxylate **2b** (94 μL, 0.6 mmol) was performed for 30 minutes.

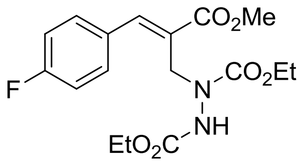

3h

3h: 87 mg, 79% yield, as yellow oil; ^{1}H NMR (400 MHz, $CDCl_3$) δ 7.76 (s, 1H), 7.33 (br s, 2H), 7.00 (m, 2H), 6.76 (br s, 1H), 4.53 (s, 2H), 4.06 (m, 4H), 3.72 (s, 3H), 1.15 (m, 6H); ^{13}C NMR (100 MHz, $CDCl_3$) δ 167.8, 162.9 (d, $J_{C\text{-}F}$ = 250.2 Hz), 155.9(2C), 142.7, 131.3 (d, $J_{C\text{-}F}$ = 7.5 Hz), 130.4, 126.8, 115.5 (d, $J_{C\text{-}F}$ = 21.7 Hz), 62.38, 61.73, 52.03, 46.27, 14.25, 14.19; IR (KBr): υ_{max} 3170, 2984, 1718, 1401, 1124 cm^{-1}; HRMS (ESI) calcd for $C_{17}H_{22}FN_2O_6$ $[M+H]^+$ requires 369.1462, found 369.1464.

Table 2, entry 8. Following the general procedure, the reaction of MBH alcohol **1g** (75 mg, 0.3 mmol), PPh_3 (157 mg, 0.6 mmol) and di-*tert*-butyl azodicarboxylate **2c** (138 mg, 0.6 mmol) was performed for 40 minutes.

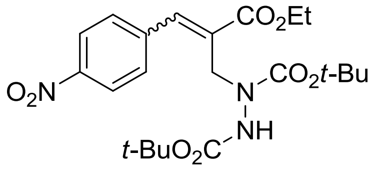

3i

3i: obtained as an inseparable mixture of *E*/*Z* isomers (*E*/*Z* 3:1) in 120 mg, 86% yield, as colorless oil; NMR data for *E*–**3i**: ^{1}H NMR (400 MHz, $CDCl_3$) δ 8.23 (d, *J* = 8.2 Hz, 2H), 7.85 (s, 1H), 7.61 (m, 2H), 6.39 (br m, 1H), 4.56 (s, 2H), 4.30 (q, *J* = 7.1 Hz, 2H), 1.46 (m, 18H), 1.36 (t, *J* = 7.1 Hz, 3H); ^{13}C NMR (100 MHz, $CDCl_3$) δ 166.8, 154.8(2C), 147.4, 141.1, 140.3, 130.0, 129.0, 123.4, 81.3, 81.0, 61.2, 45.3, 27.9(2C), 14.0; selected NMR data for *Z*–**3i**: ^{1}H NMR (400 MHz, $CDCl_3$) δ 8.18 (d, *J* = 8.3 Hz, 2H), 7.46 (d, *J* = 8.3 Hz, 2H), 6.94 (s, 1H), 4.48 (s, 2H), 4.16 (q, *J* = 7.1 Hz, 2H); ^{13}C NMR (100 MHz, $CDCl_3$) δ 147.0, 142.1, 131.1, 123.1, 61.1, 13.6; IR (KBr): υ_{max} 3433, 2986, 1521, 1710, 1401, 1369, 1122cm^{-1}; HRMS (ESI) calcd for $C_{22}H_{31}N_3NaO_8$ $[M+Na]^+$ requires 488.2009, found 488.2003.

Table 2, entry 9. Following the general procedure, the reaction of MBH alcohol **1h**

(47 mg, 0.3 mmol), PPh_3 (157 mg, 0.6 mmol) and diisopropyl azodicarboxylate **2a** (118 μL, 0.6 mmol) was performed for 30 minutes.

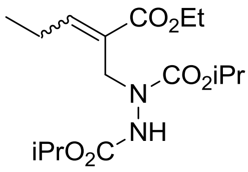

3j

3j: obtained as an inseparable mixture of *E*/*Z* isomers (*E*/*Z* 9:1) in 83 mg, 80 % yield, as slightly yellow oil; NMR data for *E*–**3j**: ^{1}H NMR (400 MHz, $CDCl_3$) δ 6.98 (t, J = 7.0 Hz, 1H), 6.56 (br m, 1H), 5.00 (m, 2H), 4.35 (m, 2H), 4.19 (q, J = 7.1 Hz, 2H), 2.31 (br s, 2H), 1.29 (t, J = 7.1 Hz, 3H), 1.25 (m, 12H), 1.06 (t, J = 7.6 Hz, 3H); ^{13}C NMR (100 MHz, $CDCl_3$) δ 167.0, 155.4(2C), 149.8, 126.3, 69.7, 69.3, 60.5, 44.9, 21.9, 21.81, 21.76, 14.1, 13.1; selected NMR data for *Z*–**3j**: ^{1}H NMR (400 MHz, $CDCl_3$) δ 6.10 (br s, 1H), 4.29 (s, 2H), 2.51 (m, 2H); ^{13}C NMR (100 MHz, $CDCl_3$) δ 149.5, 126.2, 70.3, 60.3, 44.5, 21.6, 13.5; IR (KBr): υ_{max} 3437, 2982, 1715, 1401, 1135 cm^{-1}; HRMS (ESI) calcd for $C_{16}H_{29}N_2O_6$ $[M+H]^+$ requires 345.2026, found 345.2020.

Table 2, entry 10. Following the general procedure, the reaction of MBH alcohol **1h** (47 mg, 0.3 mmol), PPh_3 (157 mg, 0.6 mmol) and diethyl azodicarboxylate **2b** (94 μL, 0.6 mmol) was performed for 20 minutes.

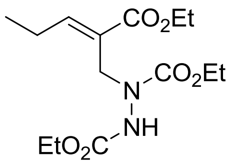

3k

3k: 78 mg, 82% yield, as colorless oil; ^{1}H NMR (400 MHz, $CDCl_3$) δ 6.99 (m, 2H), 4.43 (s, 2H), 4.18 (m, 6H), 2.32 (br s, 2H), 1.28 (m, 9H), 1.06 (t, J = 7.5 Hz, 3H); ^{13}C NMR (100 MHz, $CDCl_3$) δ 166.9, 155.7(2C), 149.7, 126.0, 62.1, 61.6, 60.5, 45.0, 21.6, 14.3, 14.2, 13.9, 12.9; IR (KBr): υ_{max} 3434, 2983, 1712, 1401, 1124 cm^{-1}; HRMS (ESI) calcd for $C_{14}H_{25}N_2O_6$ $[M+H]^+$ requires 317.1713, found 317.1712.

Table 2, entry 11. Following the general procedure, the reaction of MBH alcohol **1i** (43 mg, 0.3 mmol), PPh_3 (157 mg, 0.6 mmol) and diethyl azodicarboxylate **2b** (94 μL, 0.6 mmol) was performed for 25 minutes.

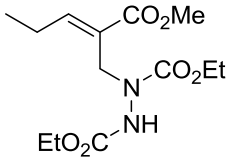

3l

3l: 72 mg, 80% yield, as slightly yellow oil; ^{1}H NMR (400 MHz, $CDCl_3$) δ 6.94 (m,2H), 4.37 (s, 2H), 4.13 (m, 4H), 3.69 (s, 3H), 2.28 (m, 2H), 1.22 (t, *J* = 7.1 Hz, 6H), 1.02 (t, *J* = 7.5 Hz, 3H); ^{13}C NMR (100 MHz, $CDCl_3$) δ 167.4, 156.0(2C), 150.3, 125.8, 62.2, 61.6, 51.7, 45.2, 21.7, 14.33, 14.28, 13.0; IR (KBr): υ_{max} 3448, 2981, 1718, 1401, 1122cm^{-1}; HRMS (ESI) calcd for $C_{13}H_{23}N_2O_6$ $[M+H]^+$ requires 303.1556, found 303.1554.

Table 2, entry 12. Following the general procedure, the reaction of MBH alcohol **1i** (43 mg, 0.3 mmol), PPh_3 (157 mg, 0.6 mmol) and diisopropyl azodicarboxylate **2a** (118 μL, 0.6 mmol) was performed for 30 minutes.

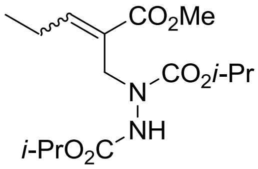

3m

3m: obtained as an inseparable mixture of *E*/*Z* isomers (*E*/*Z* 13:1) in 80 mg, 81% yield, as slightly yellow oil; NMR data for *E*–**3m**: ^{1}H NMR (400 MHz, $CDCl_3$) δ 6.98 (br s, 1H), 6.54 (br m, 1H), 4.95 (m, 2H), 4.41 (s, 2H), 3.74 (s, 3H), 2.31 (m, 2H), 1.26 (m, 12H), 1.06 (t, *J* = 7.4 Hz, 3H); ^{13}C NMR (100 MHz, $CDCl_3$) δ 167.4, 155.6(2C), 150.2, 126.0, 69.8, 69.3, 51.7, 45.0, 21.9, 21.8, 21.5, 13.0; selected NMR data for *Z*–**3m**: ^{1}H NMR (400 MHz, $CDCl_3$) δ 6.12 (br s, 1H), 4.19 (s, 2H), 2.51 (m, 2H); ^{13}C NMR (100 MHz, $CDCl_3$) δ 149.8, 125.8, 51.3, 44.5, 13.4; IR (KBr): υ_{max} 3427, 2981, 1718, 1401, 1144 cm^{-1}; HRMS (ESI) calcd for $C_{15}H_{27}N_2O_6$ $[M+H]^+$ requires 331.1869, found 331.1864.

Table 2, entry 13. Following the general procedure, the reaction of MBH alcohol **1j** (43 mg, 0.3 mmol), PPh_3 (157 mg, 0.6 mmol) and diethyl azodicarboxylate **2b** (94 μL, 0.6 mmol) was performed for 21 minutes.

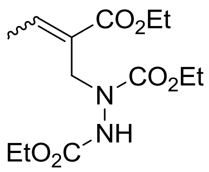

3n

3n: obtained as an inseparable mixture of *E*/*Z* isomers (*E*/*Z* 13:1) in 75 mg, 83% yield, as colorless oil; NMR data for *E*–**3n**: ^{1}H NMR (400 MHz, $CDCl_3$) δ 7.11 (q, *J* = 6.9 Hz, 1H), 6.70 (br m, 1H), 4.34 (s, 2H), 4.19 (m, 6H), 1.92 (m, 3H), 1.28 (m, 9H); ^{13}C NMR (100 MHz, $CDCl_3$) δ 166.9, 155.8(2C), 143.5, 127.8, 62.4, 61.8, 60.6, 45.2, 14.43, 14.38, 14.3, 14.1; selected NMR data for *Z*–**3n**: ^{1}H NMR (400 MHz, $CDCl_3$) δ 6.26 (br m, 1H), 2.05 (d, *J* = 7.2 Hz, 3H); ^{13}C NMR (100 MHz, $CDCl_3$) δ 64.0, 60.4, 15.6; IR (KBr): υ_{max} 3427, 2985, 1712, 1401, 1137 cm^{-1}; HRMS (ESI) calcd for $C_{13}H_{23}N_2O_6$ $[M+H]^+$ requires 303.1556, found 303.1555.

Table 2, entry 14. Following the general procedure, the reaction of MBH alcohol **1j** (43 mg, 0.3 mmol), PPh_3 (157 mg, 0.6 mmol) and di-*tert*-butyl azodicarboxylate **2c** (138 mg, 0.6 mmol) was performed for 40 minutes.

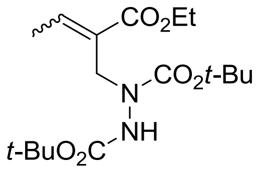

3o

3o: obtained as an inseparable mixture of *E*/*Z* isomers (*E*/*Z* 5:1) in 59 mg, 55% yield, as colorless oil; NMR data for *E*–**3o**: ^{1}H NMR (400 MHz, $CDCl_3$) δ 7.09 (q, *J* = 6.9 Hz, 1H), 6.24 (br m, 1H), 4.41 (s, 2H), 4.19 (q, *J* = 7.1 Hz, 2H), 1.90 (d, *J* = 6.9 Hz, 3H), 1.46 (m, 18H), 1.30 (t, *J* = 7.1 Hz, 3H); ^{13}C NMR (100 MHz, $CDCl_3$) δ 166.9, 155.0(2C), 143.1, 128.3, 80.8(2C), 60.5, 44.2, 28.1(2C), 14.2, 14.1; selected NMR data for *Z*–**3o**: ^{1}H NMR (400 MHz, $CDCl_3$) δ 6.07 (br s, 1H), 2.04 (d, *J* = 7.2 Hz, 3H), 1.14 (t, *J* = 7.2 Hz, 3H); ^{13}C NMR (100 MHz, $CDCl_3$) δ 142.6, 128.1, 60.2, 29.6, 15.6;

IR (KBr): υ_{max} 3432, 2980, 1711, 1400, 1152 cm^{-1}; HRMS (ESI) calcd for $C_{17}H_{31}N_2O_6$ $[M+H]^+$ requires 359.2182, found 359.2177.

Table 2, entry 15. Following the general procedure, the reaction of MBH alcohol **1k** (39 mg, 0.3 mmol), PPh_3 (157 mg, 0.6 mmol) and diethyl azodicarboxylate **2b** (94 μL, 0.6 mmol) was performed for 10 minutes.

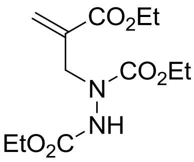

3p

3p: 78 mg, 90% yield, as colorless oil; ^{1}H NMR (400 MHz, $CDCl_3$) δ 7.12 (br m, 1H), 6.33 (s, 1H), 5.78 (s, 1H), 4.37 (s, 2H), 4.20 (m, 6H), 1.28 (m, 9H); ^{13}C NMR (100 MHz, $CDCl_3$) δ 165.9, 156.0(2C), 135.3, 128.0, 62.4, 61.8, 60.8, 51.3, 14.28, 14.24, 13.9; IR (KBr): υ_{max} 3417, 2983, 1718, 1401, 1138 cm^{-1}; HRMS (ESI) calcd for $C_{12}H_{21}N_2O_6$ $[M+H]^+$ requires 289.1400, found 289.1398.

Table 2, entry 16. Following the general procedure, the reaction of MBH alcohol **1k** (39 mg, 0.3 mmol), PPh_3 (157 mg, 0.6 mmol) and diisopropyl azodicarboxylate **2a** (118 μL, 0.6 mmol) was performed for 20 minutes.

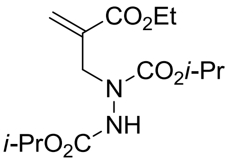

3q

3q: 84 mg, 89% yield, as colorless oil; ^{1}H NMR (400 MHz, $CDCl_3$) δ 7.10 (br m, 1H), 6.24 (s, 1H), 5.69 (s, 1H), 4.86 (m, 2H), 4.27 (s, 2H), 4.13 (q, J = 7.1 Hz, 2H), 1.22 (t, J = 7.1 Hz, 3H), 1.16 (d, J = 6.3 Hz, 12H); ^{13}C NMR (100 MHz, $CDCl_3$) δ 165.8, 155.6(2C), 135.4, 127.6, 69.8, 69.3, 60.6, 50.9, 21.73, 21.67, 13.9; IR (KBr): υ_{max} 3448, 2983, 1718, 1401, 1108 cm^{-1}; HRMS (ESI) calcd for $C_{14}H_{25}N_2O_6$ $[M+H]^+$ requires 317.1713, found 317.1713.

Table 2, entry 17. Following the general procedure, the reaction of MBH alcohol **1k** (39 mg, 0.3 mmol), PPh_3 (157 mg, 0.6 mmol) and di-*tert*-butyl azodicarboxylate **2c** (138 mg, 0.6 mmol) was performed for 25 minutes.

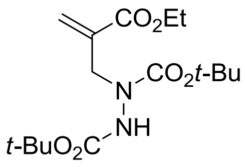

3r

3r: 93 mg, 90% yield, as colorless oil; ^{1}H NMR (400 MHz, $CDCl_3$) δ 6.50 (br m, 1H), 6.31 (s, 1H), 5.73 (s, 1H), 4.32 (s, 2H), 4.22 (q, J = 7.1 Hz, 2H), 1.47 (s, 18H), 1.30 (t, J = 7.1 Hz, 3H); ^{13}C NMR (100 MHz, $CDCl_3$) δ 166.0, 155.0(2C), 135.9, 127.3, 81.2(2C), 60.7, 50.9, 28.1(2C), 14.1; IR (KBr): υ_{max} 3344, 2981, 1713, 1400, 1152 cm^{-1}; HRMS (ESI) calcd for $C_{16}H_{28}N_2NaO_6$ $[M+Na]^+$ requires 367.1845, found 367.1840.

Table 2, entry 18. Following the general procedure, the reaction of MBH alcohol **1l** (35 mg, 0.3 mmol), PPh_3 (157 mg, 0.6 mmol) and diethyl azodicarboxylate **2b** (94 μL, 0.6 mmol) was performed for 13 minutes.

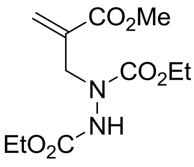

3s

3s: 72 mg, 87% yield, as slightly yellow oil; ^{1}H NMR (400 MHz, $CDCl_3$) δ 6.84 (br m, 1H), 6.34 (s, 1H), 5.79 (m, 1H), 4.37 (s, 2H), 4.19 (m, 4H), 3.77 (s, 3H), 1.27 (m, 6H); ^{13}C NMR (100 MHz, $CDCl_3$) δ 166.4, 156.1(2C), 135.1, 128.6, 62.6, 61.9, 51.9, 51.6, 14.4, 14.3; IR (KBr): υ_{max} 3449, 2981, 1719, 1401, 1139 cm^{-1}; HRMS (ESI) calcd for $C_{11}H_{19}N_2O_6$ $[M+H]^+$ requires 275.1243, found 275.1243..

2. Analytical data for 1*H*-pyrazole 7 and mechanistic rationale

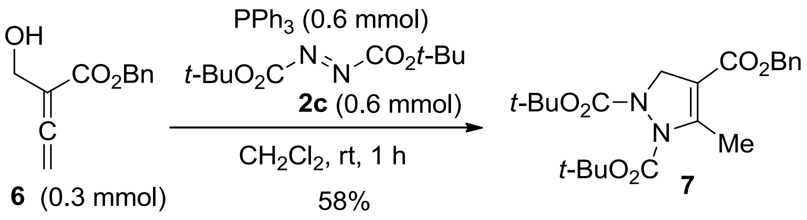

7: ^{1}H NMR (400 MHz, $CDCl_3$) δ 7.36 (m, 5H), 5.20 (s, 2H), 4.49 (s, 2H), 2.59 (s, 3H), 1.51 (s, 9H), 1.49(s, 9H); ^{13}C NMR (100 MHz, $CDCl_3$) δ 163.7, 156.4, 151.5, 151.2, 136.0, 128.6, 128.2, 128.1, 107.4, 83.2, 82.4, 65.9, 52.4, 28.1, 28.0, 14.6; IR (KBr): υ_{max} 2981, 1716, 1637, 1401, 1144 cm^{-1}; HRMS (ESI) calcd for $C_{22}H_{30}N_2NaO_6$ $[M+Na]^+$ requires 441.2002, found 441.2005.

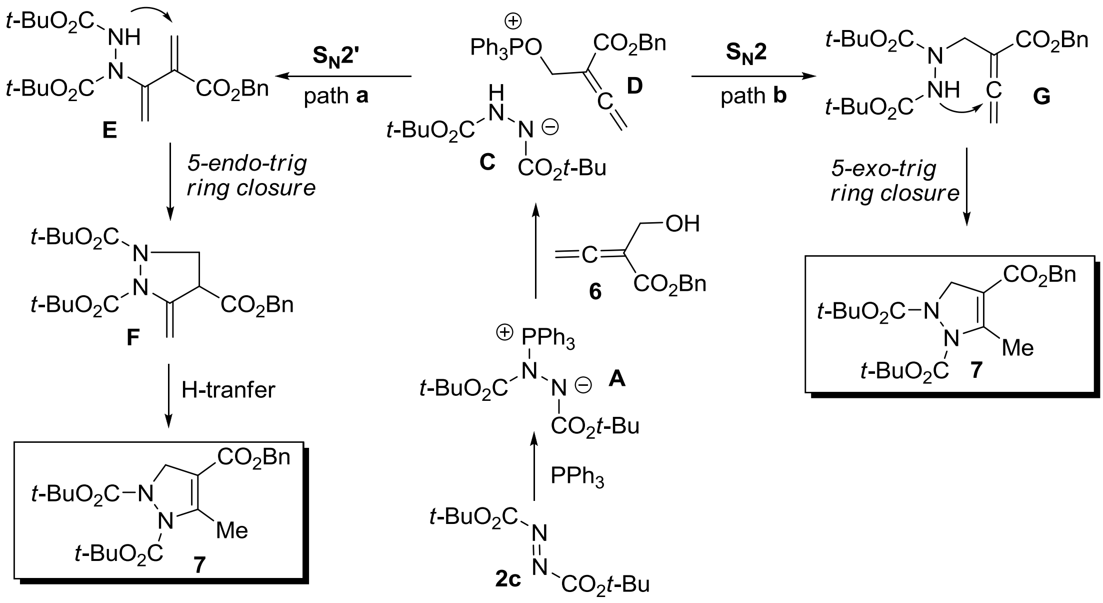

Scheme S1: Plausible mechanism for the formation of compound **7**.

The formation of compound **7** from the reaction between azodicarboxylate **2c**, allenic MBH alcohol **6**, and PPh_3 can be tentatively rationalized in Scheme S1. Initially, addition of PPh_3 to **2c** forms the Huisgen zwitterion **A**, which deprotonates the OH of **6** followed by the migration of the phosphonium to produce the anionic species **C** and the oxophosphonium intermediate **D**. In one of the possible pathways (path **a**), S_N2' attack of intermediate **C** on **D** brings about the hydrazine **E**, which undergoes a 5-*endo-trig* cyclization to furnish **F**. A final 1,3-proton transfer of **F** results in the

formation of 1*H*-pyrazole **7**. Another possible pathway for the formation of **7** (path **b**) involves the S_N2 displacement of **D** with the species **C** generating the allenic hydrazine **G**, which undertakes a favorable 5-*exo*-*trig* ring closure to furnish the 1*H*-pyrazole **7**.

3. Copies of ^{1}H, ^{13}C, DEPT, and NOESY NMR spectra

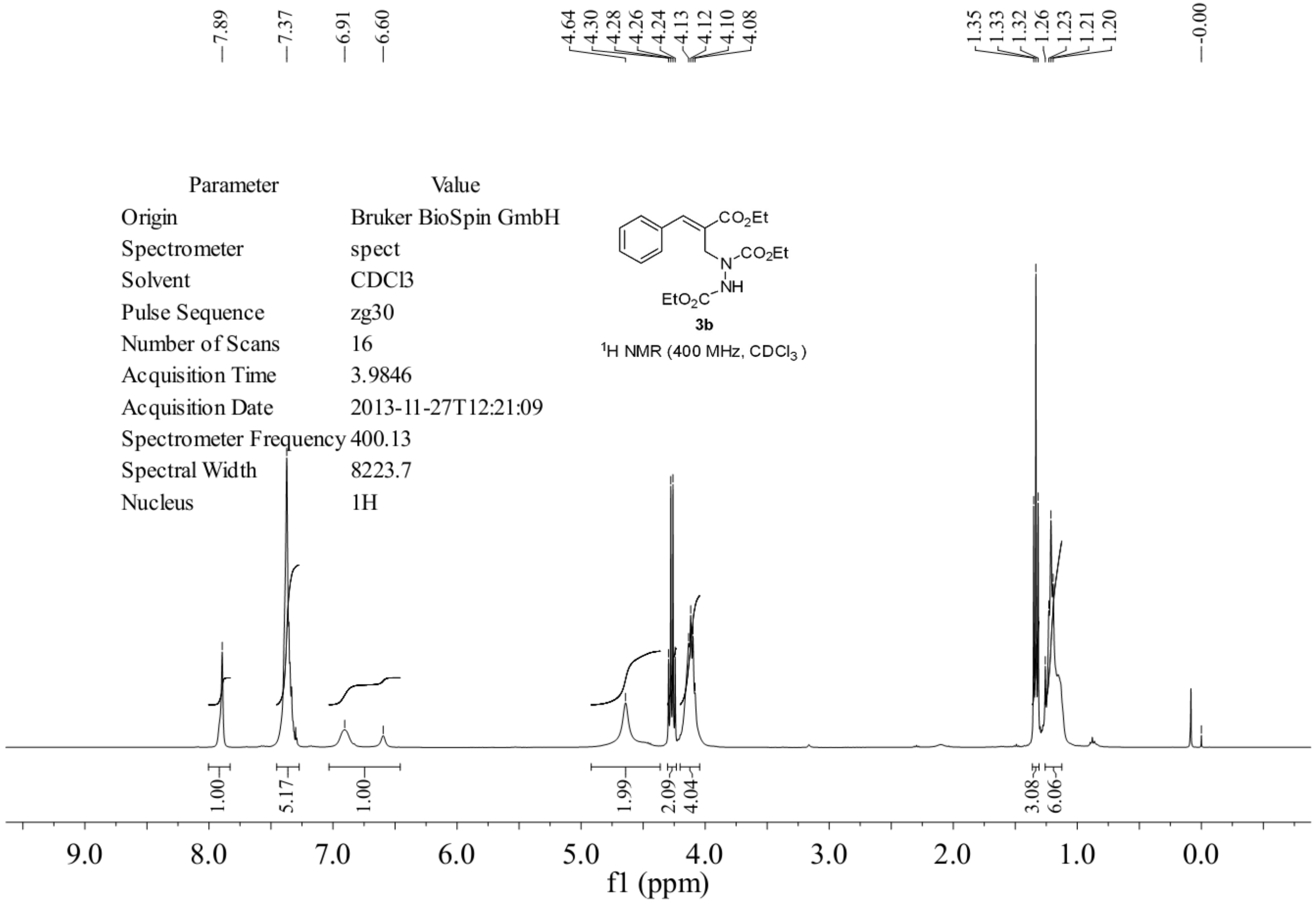

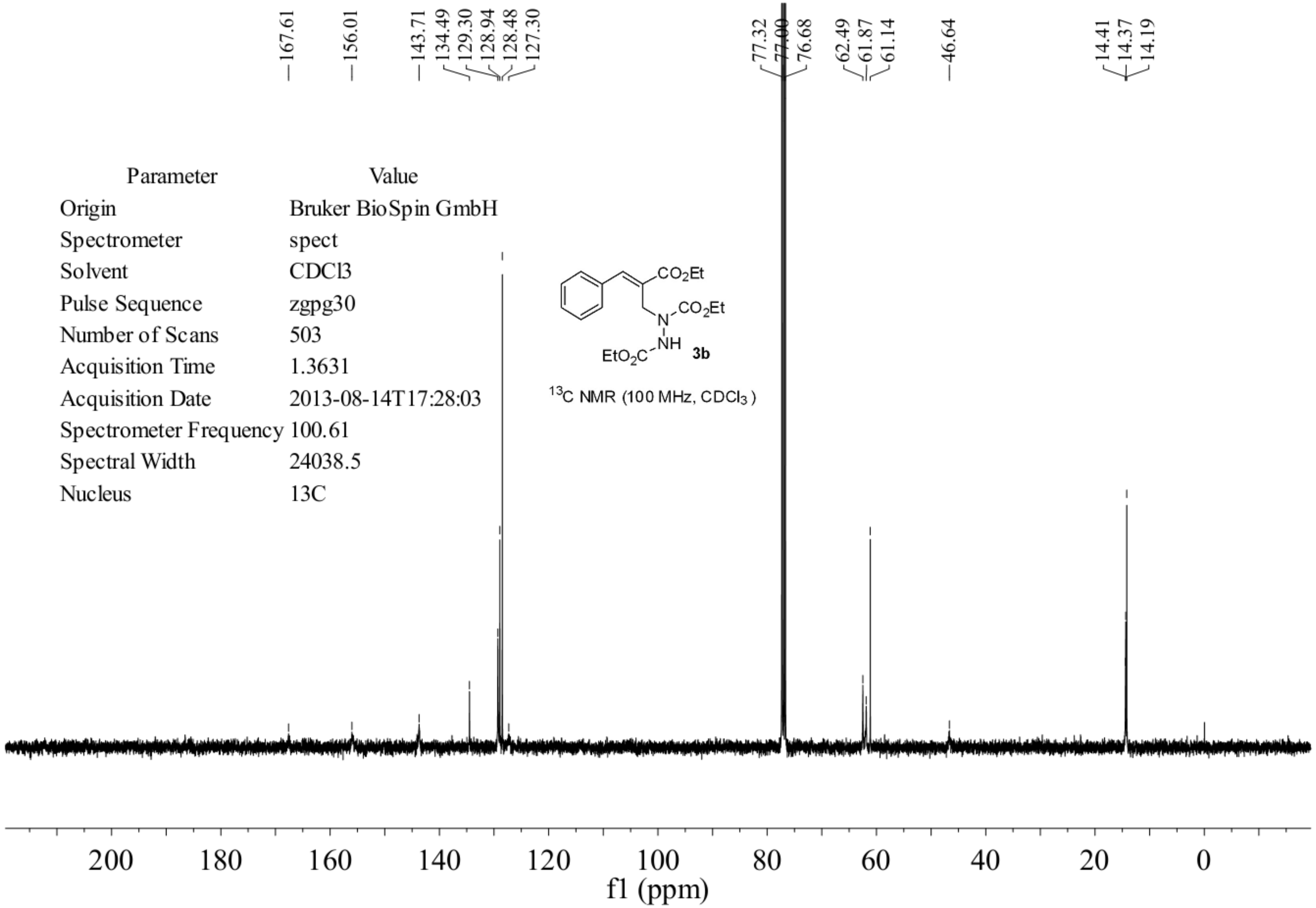
167.61
156.01
143.71
134.49
129.30
128.94
128.48
127.30
77.32
77.00
76.68
62.49
61.87
61.14
46.64
14.41
14.37
14.19
Parameter Value
Origin Bruker BioSpin GmbH
Spectrometer spect
Solvent CDCl3
Pulse Sequence zgpg30
Number of Scans 503
Acquisition Time 1.3631
Acquisition Date 2013-08-14T17:28:03
Spectrometer Frequency 100.61
Spectral Width 24038.5
Nucleus 13C
CO2Et
N
CO2Et
NH
EtO2C
3b
13C NMR (100 MHz, CDCl3)
200 180 160 140 120 100 80 60 40 20 0
f1 (ppm)

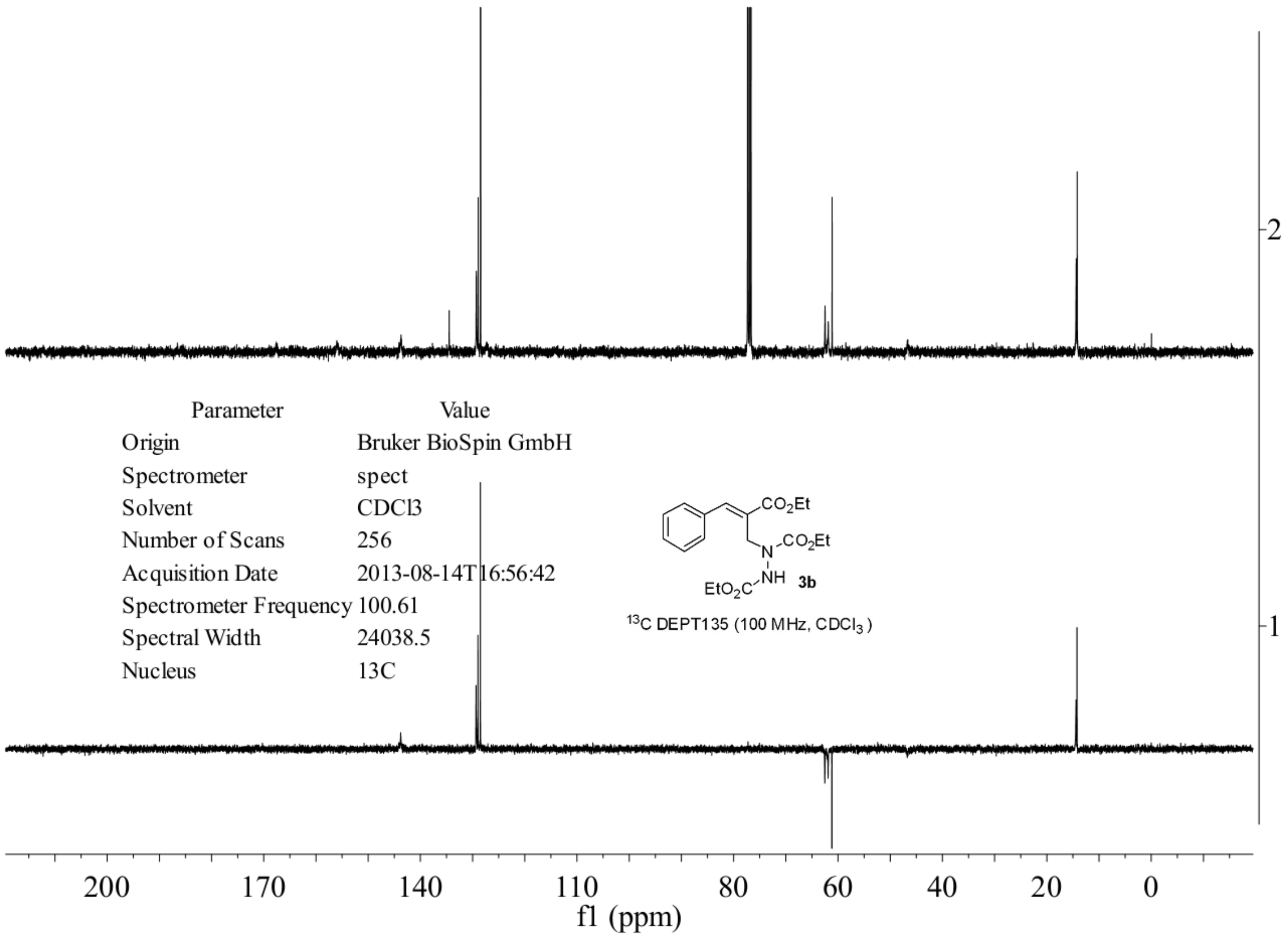
Parameter Value
Origin Bruker BioSpin GmbH
Spectrometer spect
Solvent CDCl3
Number of Scans 256
Acquisition Date 2013-08-14T16:56:42
Spectrometer Frequency 100.61
Spectral Width 24038.5
Nucleus 13C
CO2Et
N
CO2Et
NH
EtO2C
3b
13C DEPT135 (100 MHz, CDCl3)
2
1
200
170
140
110
80
60
40
20
0
f1 (ppm)

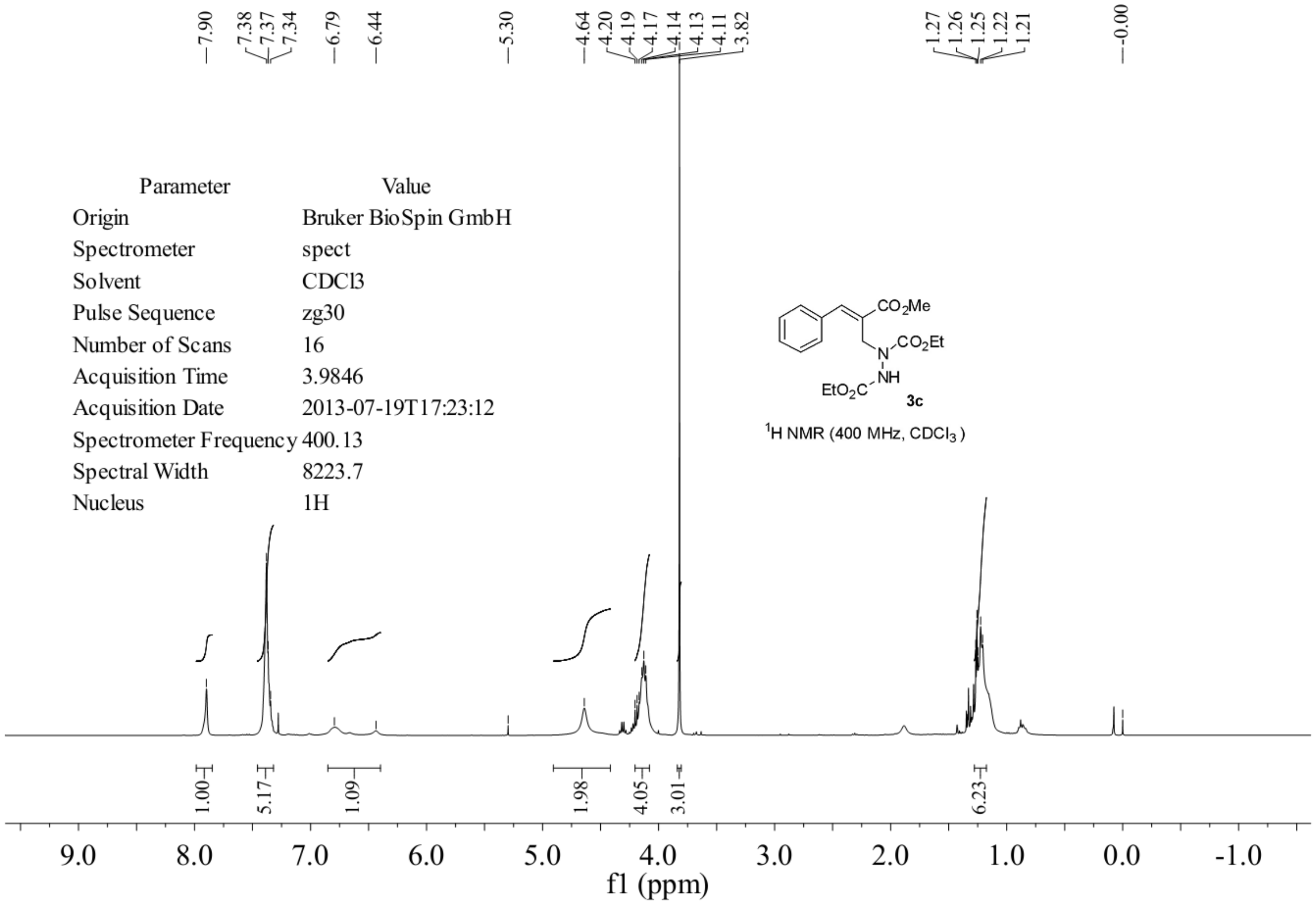
7.90
7.38
7.37
7.34
6.79
6.44
5.30
4.64
4.20
4.19
4.17
4.14
4.13
4.11
3.82
1.27
1.26
1.25
1.22
1.21
-0.00
Parameter
Value
Origin
Bruker BioSpin GmbH
Spectrometer
spect
Solvent
CDCl3
Pulse Sequence
zg30
Number of Scans
16
Acquisition Time
3.9846
Acquisition Date
2013-07-19T17:23:12
Spectrometer Frequency
400.13
Spectral Width
8223.7
Nucleus
1H
CO2Me
N
CO2Et
EtO2C
NH
3c
1H NMR (400 MHz, CDCl3)
1.00
5.17
1.09
1.98
4.05
3.01
6.23
9.0
8.0
7.0
6.0
5.0
4.0
3.0
2.0
1.0
0.0
-1.0
f1 (ppm)

Parameter	Value
Origin	Bruker BioSpin GmbH
Spectrometer	spect
Solvent	CDCl3
Pulse Sequence	zgpg30
Number of Scans	677
Acquisition Time	1.3631
Acquisition Date	2013-09-01T16:02:08
Spectrometer Frequency	100.61
Spectral Width	24038.5
Nucleus	13C

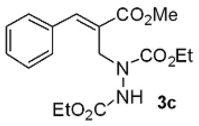

3c

^{13}C NMR (100 MHz, $CDCl_3$)

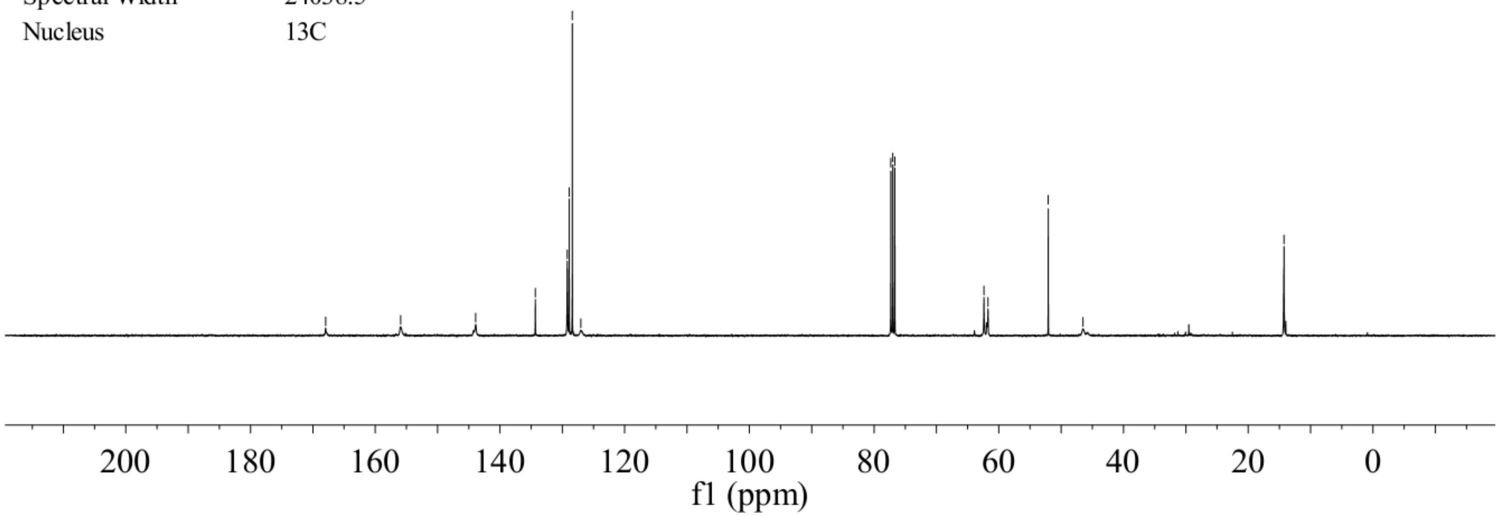

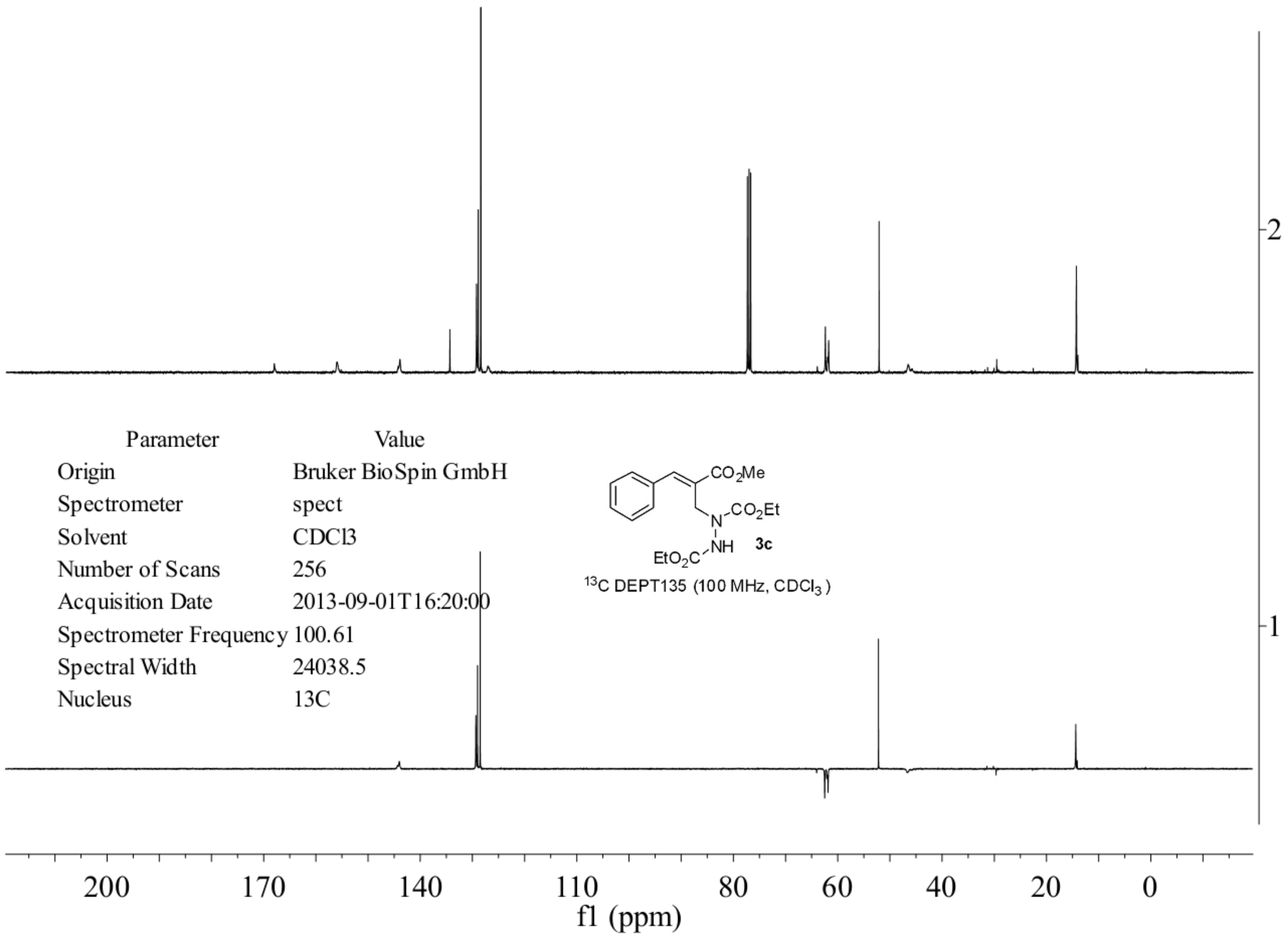
Parameter Value
Origin Bruker BioSpin GmbH
Spectrometer spect
Solvent CDCl3
Number of Scans 256
Acquisition Date 2013-09-01T16:20:00
Spectrometer Frequency 100.61
Spectral Width 24038.5
Nucleus 13C
CO2Me
N
CO2Et
EtO2C
NH
3c
13C DEPT135 (100 MHz, CDCl3)
2
1
200
170
140
110
80
60
40
20
0
f1 (ppm)

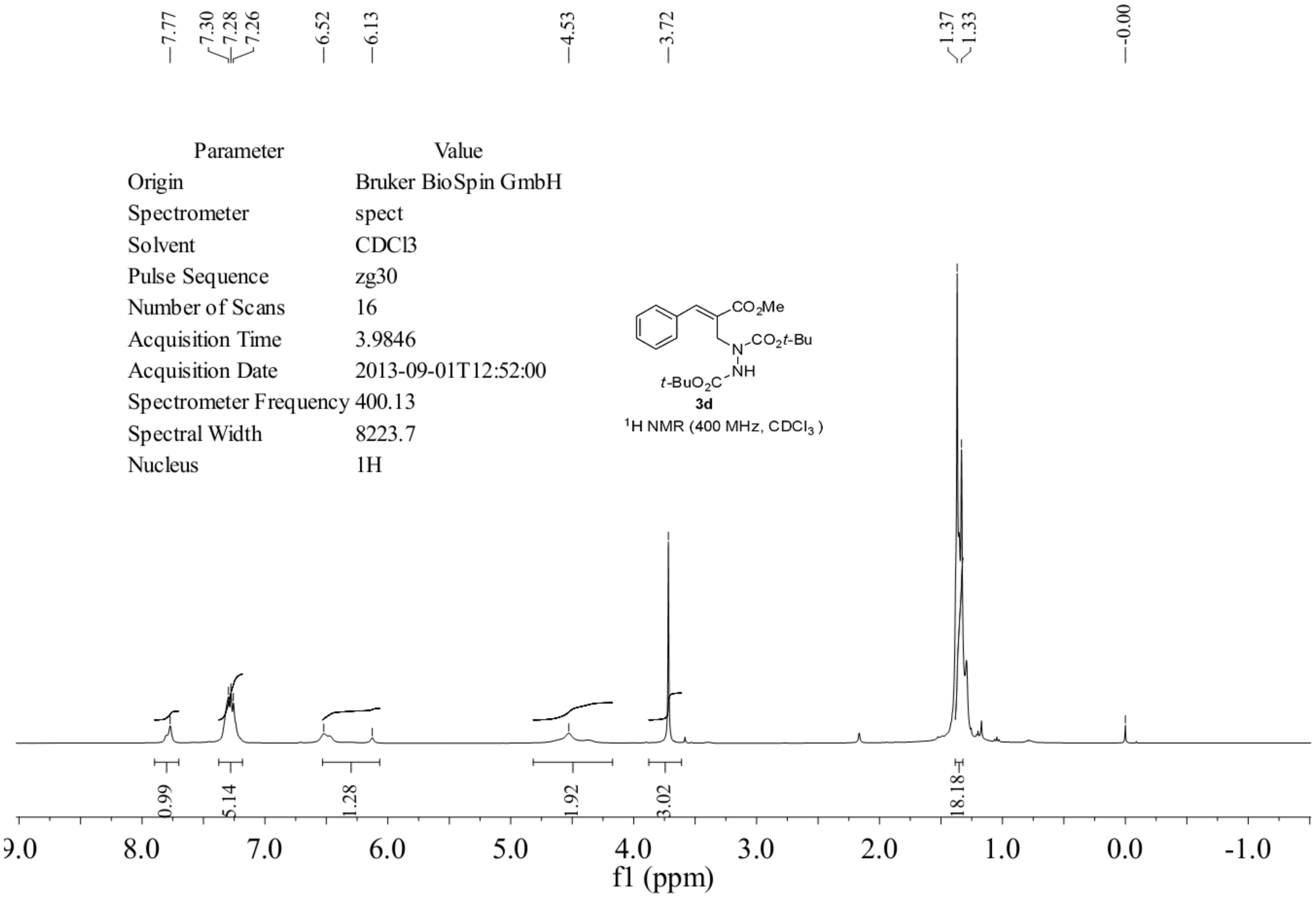

Parameter	Value
Origin	Bruker BioSpin GmbH
Spectrometer	spect
Solvent	CDCl3
Pulse Sequence	zg30
Number of Scans	16
Acquisition Time	3.9846
Acquisition Date	2013-09-01T12:52:00
Spectrometer Frequency	400.13
Spectral Width	8223.7
Nucleus	1H

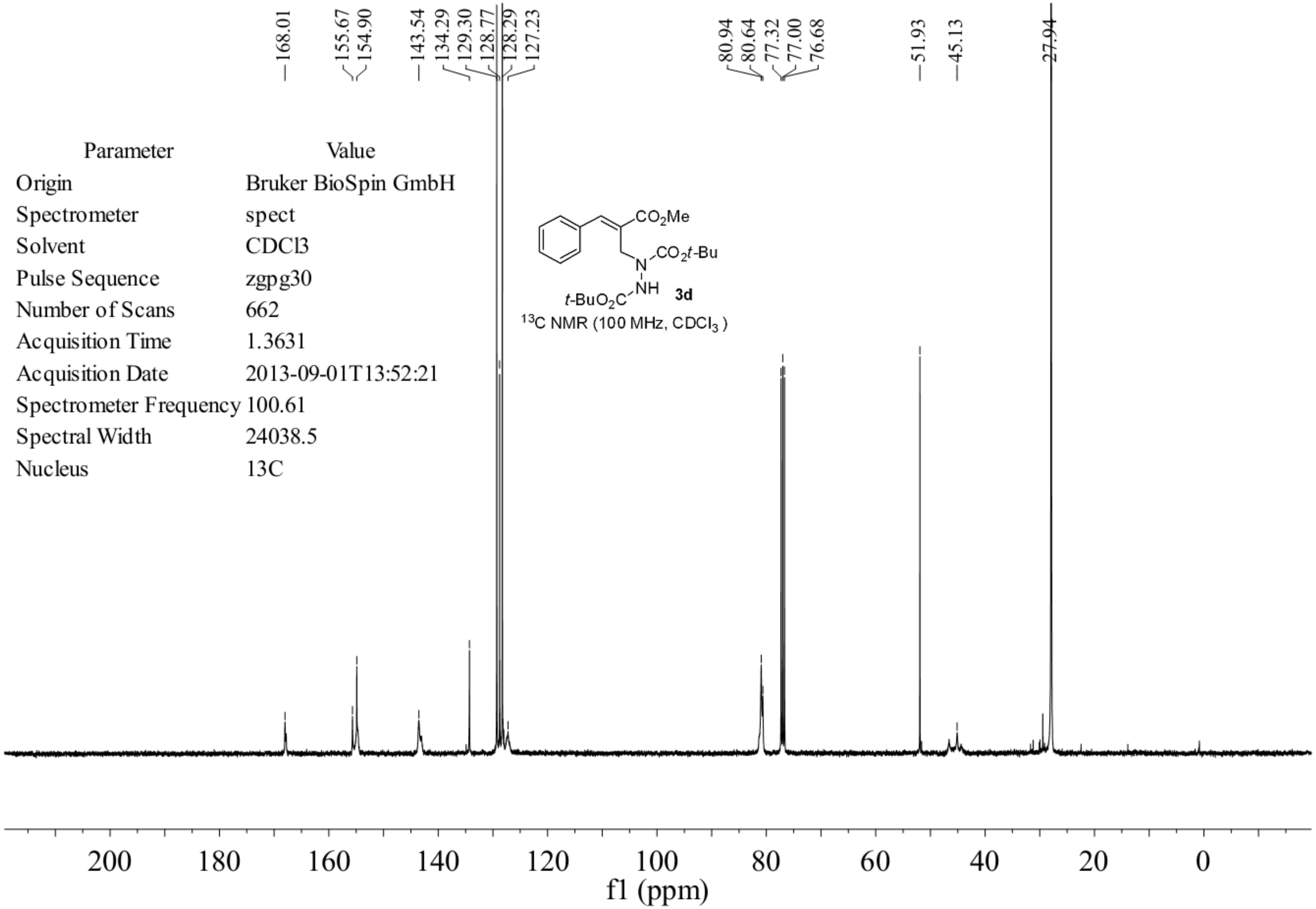
168.01
155.67
154.90
143.54
134.29
129.30
128.77
128.29
127.23
80.94
80.64
77.32
77.00
76.68
51.93
45.13
27.94
Parameter Value
Origin Bruker BioSpin GmbH
Spectrometer spect
Solvent CDCl3
Pulse Sequence zgpg30
Number of Scans 662
Acquisition Time 1.3631
Acquisition Date 2013-09-01T13:52:21
Spectrometer Frequency 100.61
Spectral Width 24038.5
Nucleus 13C
CO2Me
CO2t-Bu
N
NH
t-BuO2C
3d
13C NMR (100 MHz, CDCl3)
200
180
160
140
120
100
80
60
40
20
0
f1 (ppm)

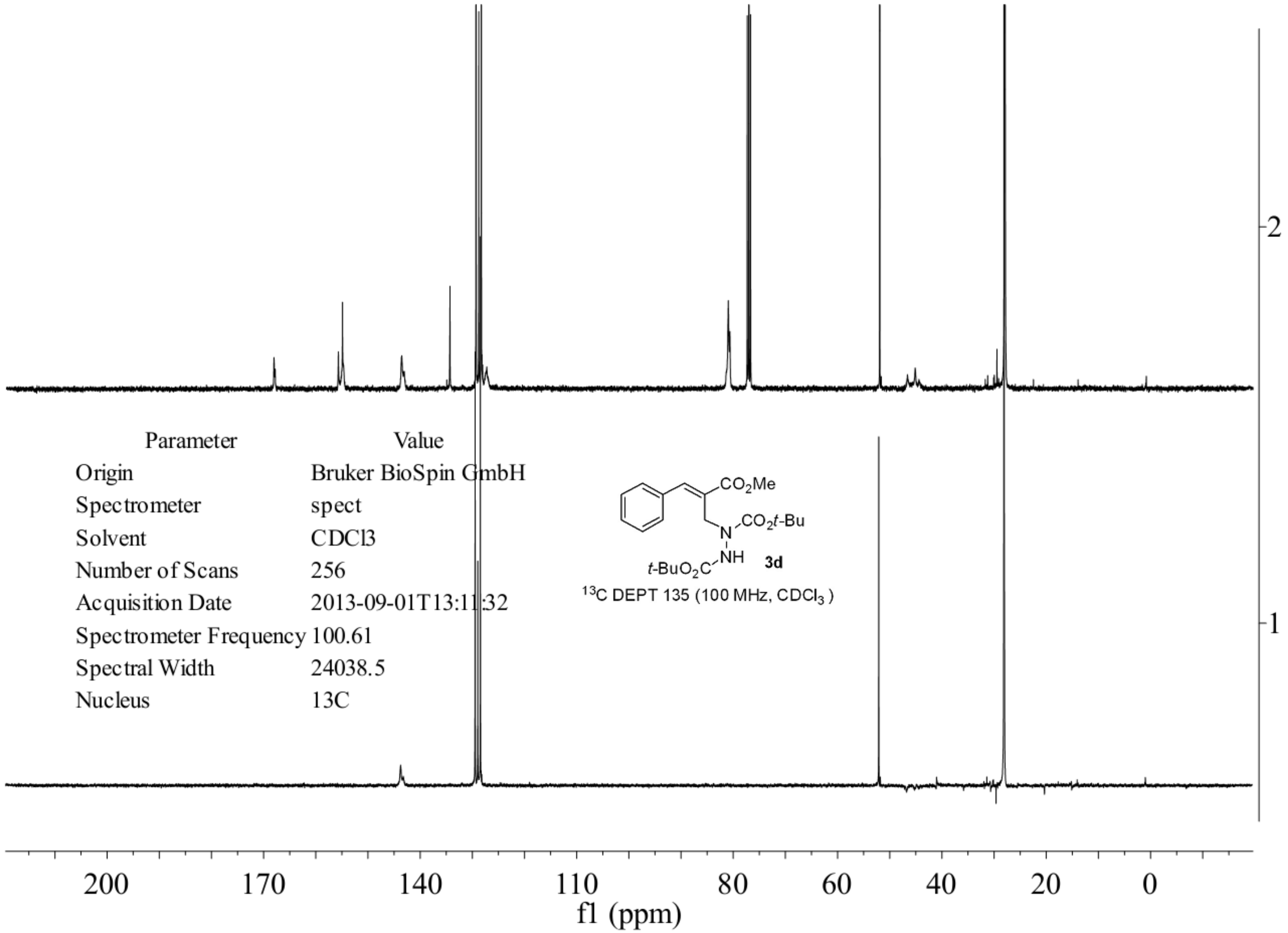
Parameter Value
Origin Bruker BioSpin GmbH
Spectrometer spect
Solvent CDCl3
Number of Scans 256
Acquisition Date 2013-09-01T13:11:32
Spectrometer Frequency 100.61
Spectral Width 24038.5
Nucleus 13C
CO2Me
CO2t-Bu
N
NH
t-BuO2C
3d
13C DEPT 135 (100 MHz, CDCl3)
2
1
200 170 140 110 80 60 40 20 0
f1 (ppm)

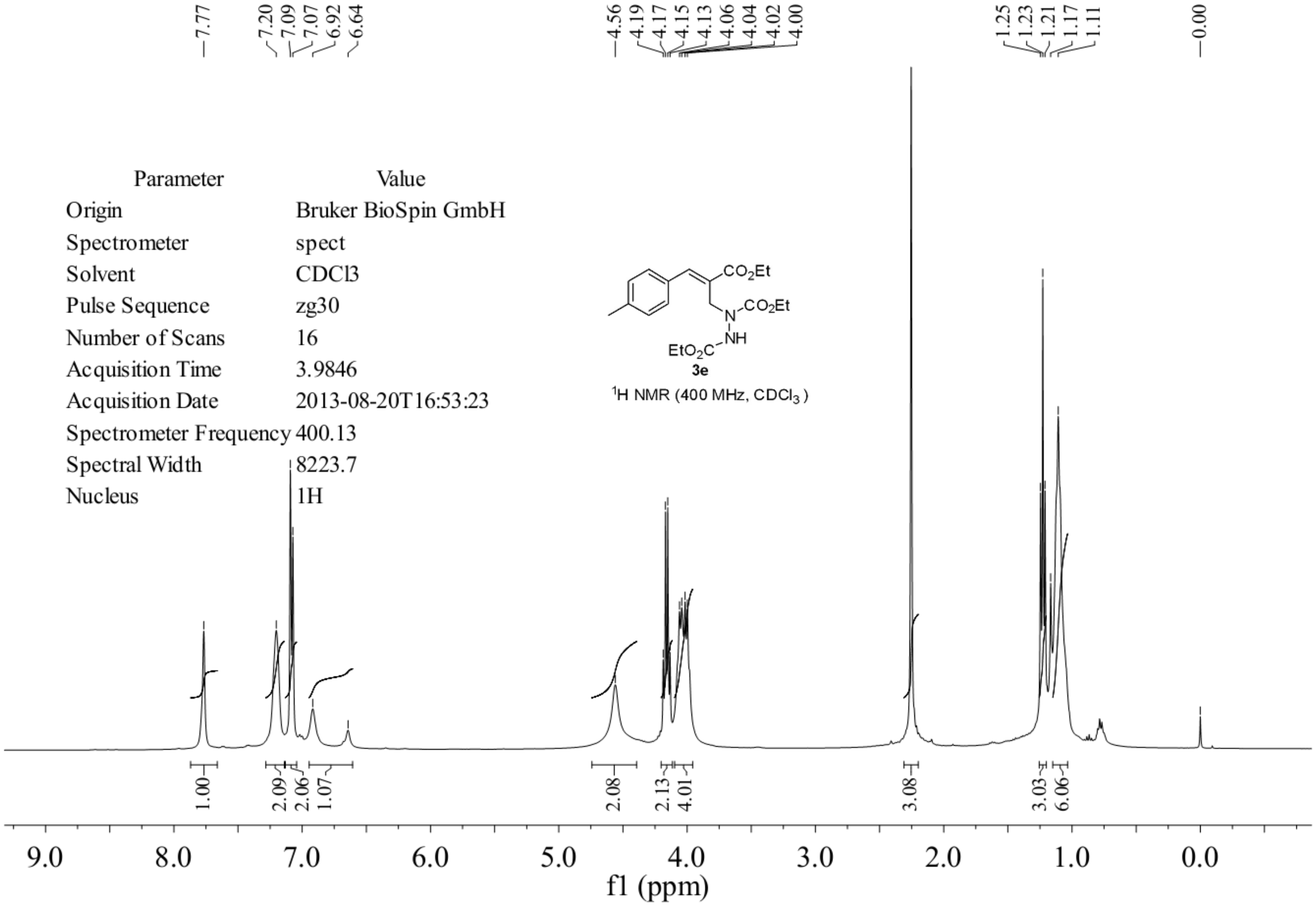

Parameter	Value
Origin	Bruker BioSpin GmbH
Spectrometer	spect
Solvent	CDCl3
Pulse Sequence	zg30
Number of Scans	16
Acquisition Time	3.9846
Acquisition Date	2013-08-20T16:53:23
Spectrometer Frequency	400.13
Spectral Width	8223.7
Nucleus	1H

Parameter	Value
Origin	Bruker BioSpin GmbH
Spectrometer	spect
Solvent	CDCl3
Pulse Sequence	zgpg30
Number of Scans	532
Acquisition Time	1.3631
Acquisition Date	2013-08-20T17:27:26
Spectrometer Frequency	100.61
Spectral Width	24038.5
Nucleus	13C

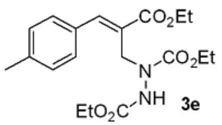

3e

^{13}C NMR (100 MHz, $CDCl_3$)

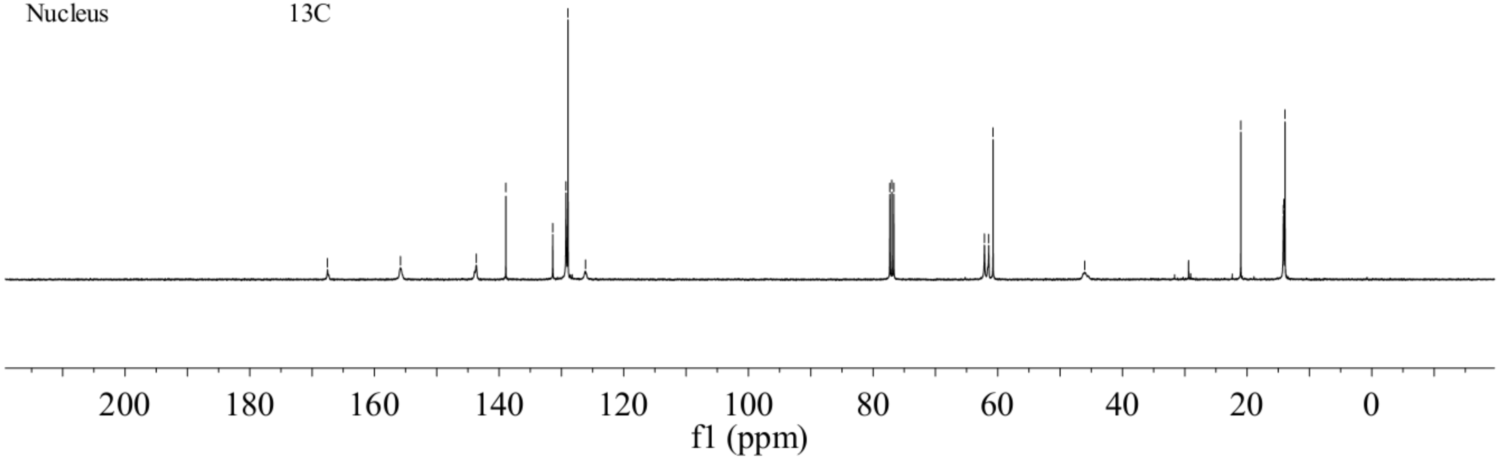

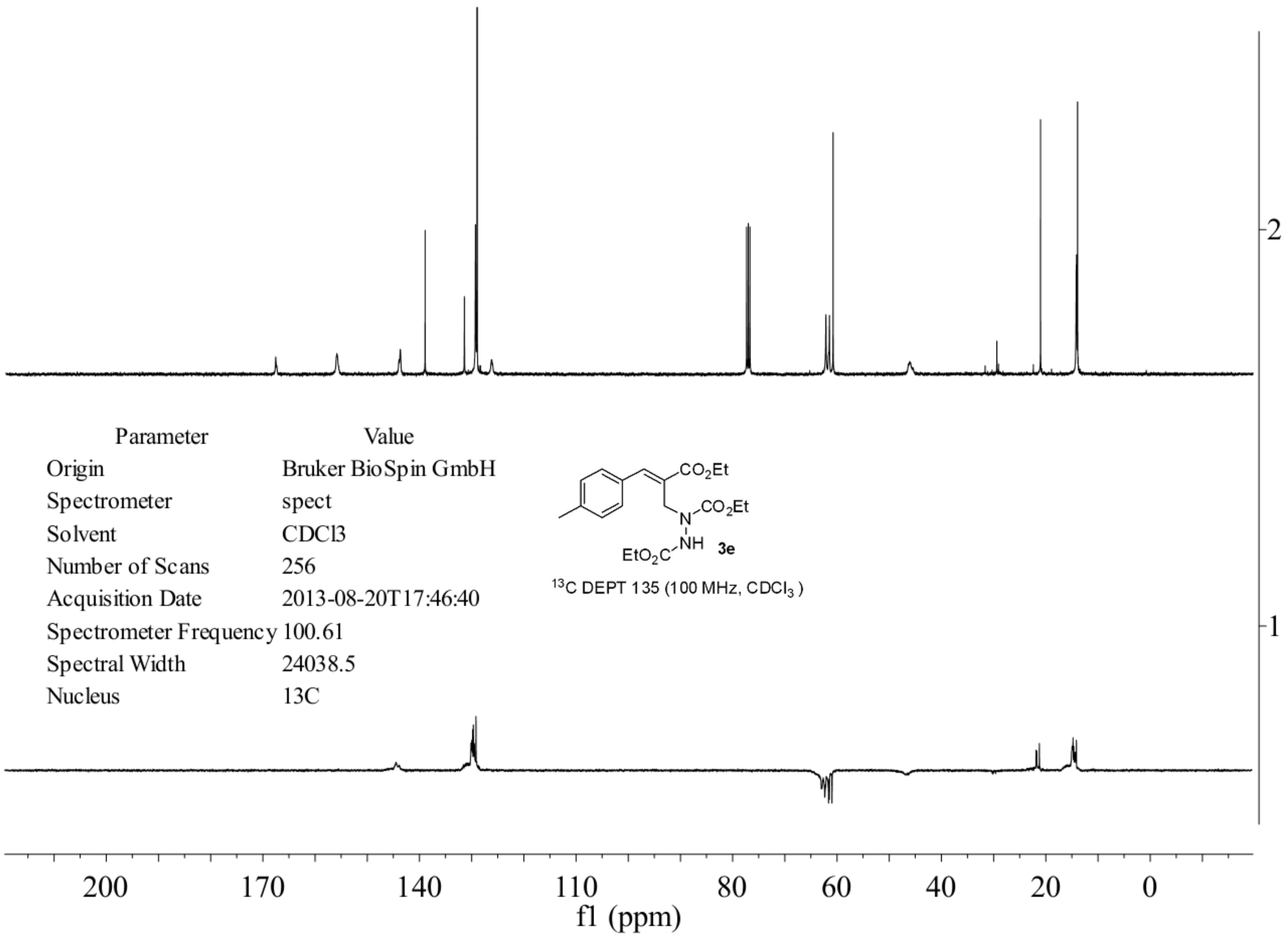
Parameter Value
Origin Bruker BioSpin GmbH
Spectrometer spect
Solvent CDCl3
Number of Scans 256
Acquisition Date 2013-08-20T17:46:40
Spectrometer Frequency 100.61
Spectral Width 24038.5
Nucleus 13C
CO2Et
N
CO2Et
NH
EtO2C
3e
13C DEPT 135 (100 MHz, CDCl3)
2
1
200
170
140
110
80
60
40
20
0
f1 (ppm)

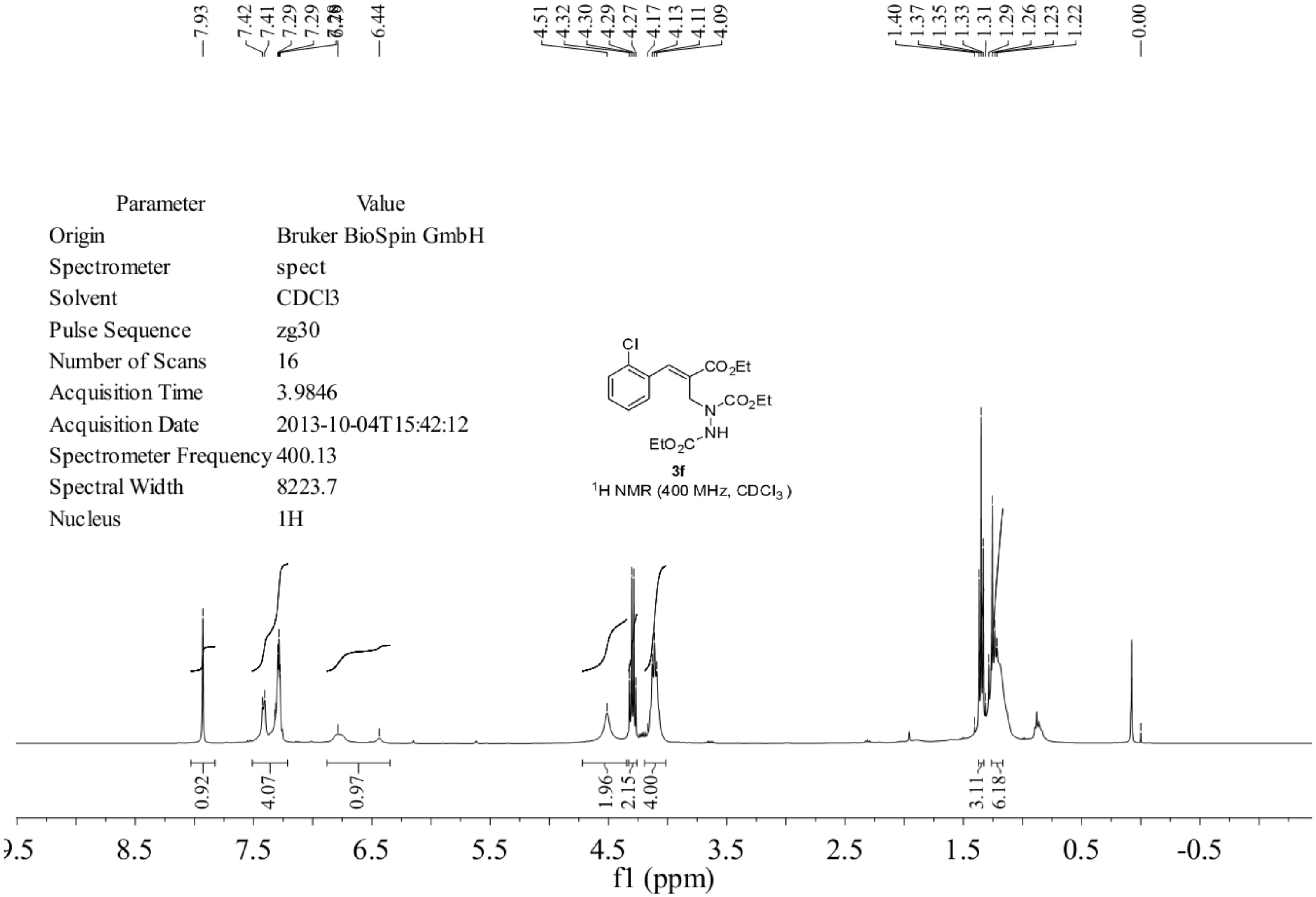

Parameter	Value
Origin	Bruker BioSpin GmbH
Spectrometer	spect
Solvent	CDCl3
Pulse Sequence	zg30
Number of Scans	16
Acquisition Time	3.9846
Acquisition Date	2013-10-04T15:42:12
Spectrometer Frequency	400.13
Spectral Width	8223.7
Nucleus	1H

3f

^1H NMR (400 MHz, $CDCl_3$)

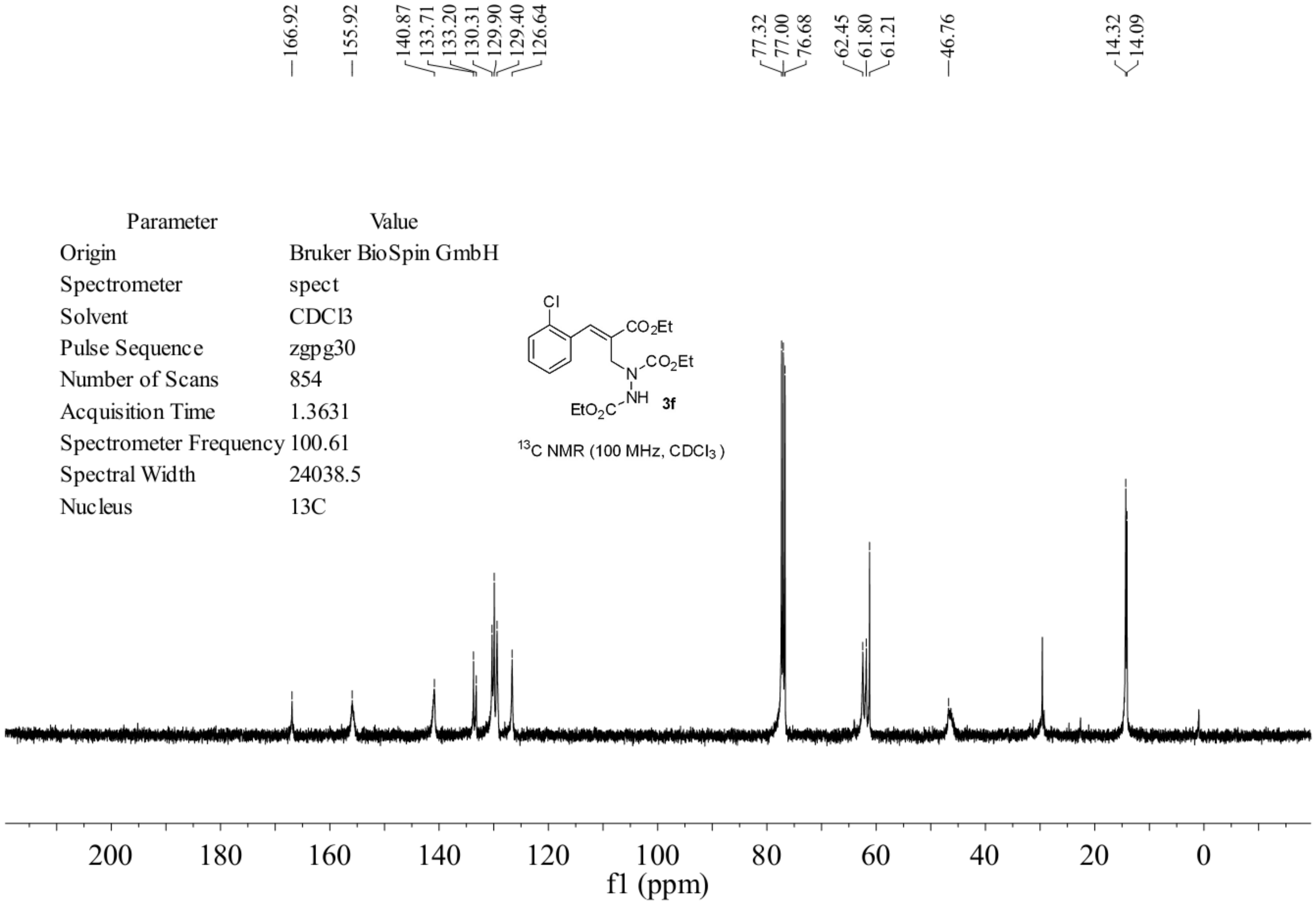

Parameter	Value
Origin	Bruker BioSpin GmbH
Spectrometer	spect
Solvent	CDCl3
Pulse Sequence	zgpg30
Number of Scans	854
Acquisition Time	1.3631
Spectrometer Frequency	100.61
Spectral Width	24038.5
Nucleus	13C

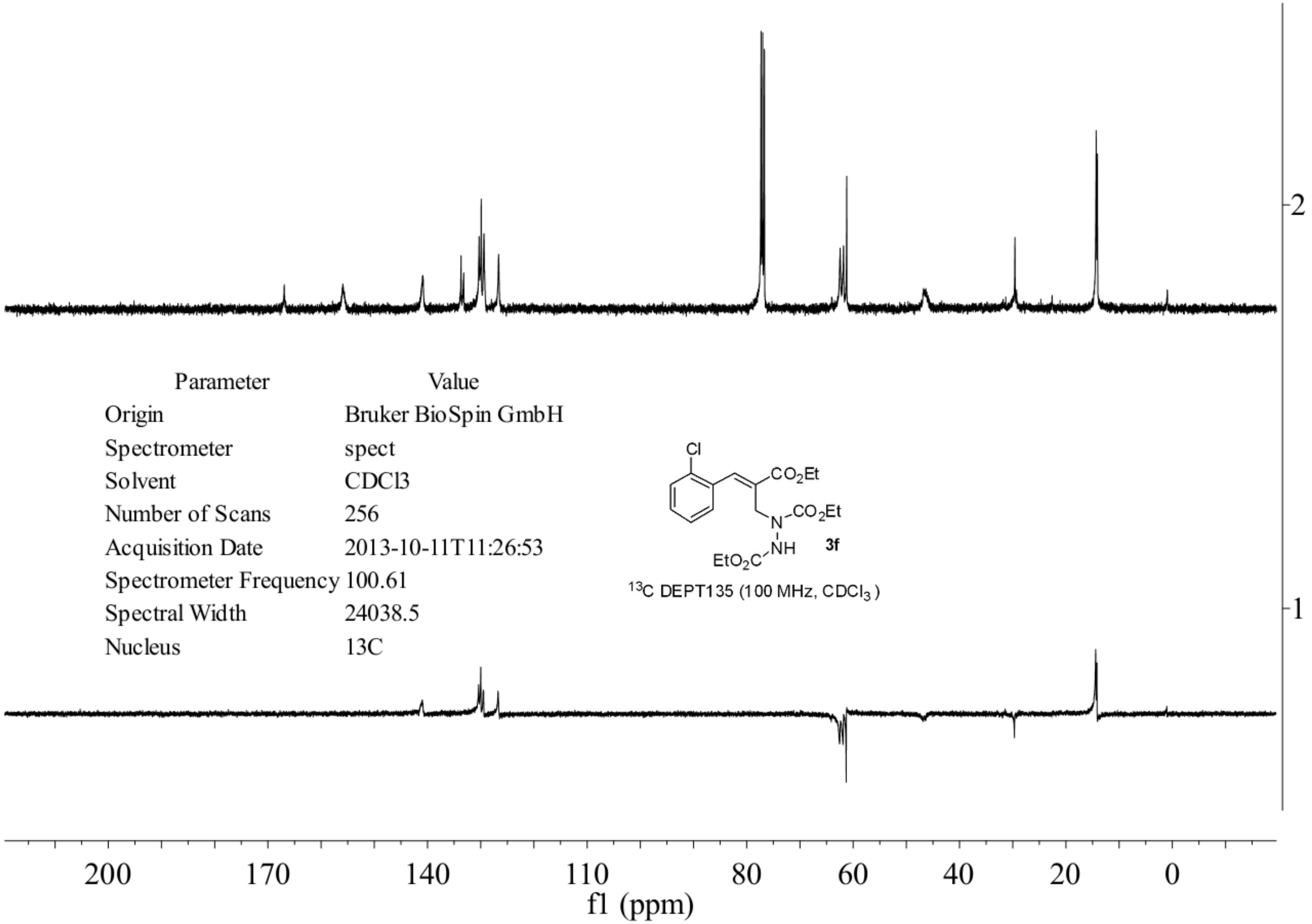
Parameter Value
Origin Bruker BioSpin GmbH
Spectrometer spect
Solvent CDCl3
Number of Scans 256
Acquisition Date 2013-10-11T11:26:53
Spectrometer Frequency 100.61
Spectral Width 24038.5
Nucleus 13C
Cl
CO_2Et
N
CO_2Et
NH
EtO_2C
3f
^{13}C DEPT135 (100 MHz, $CDCl_3$)
2
1
200
170
140
110
80
60
40
20
0
f1 (ppm)

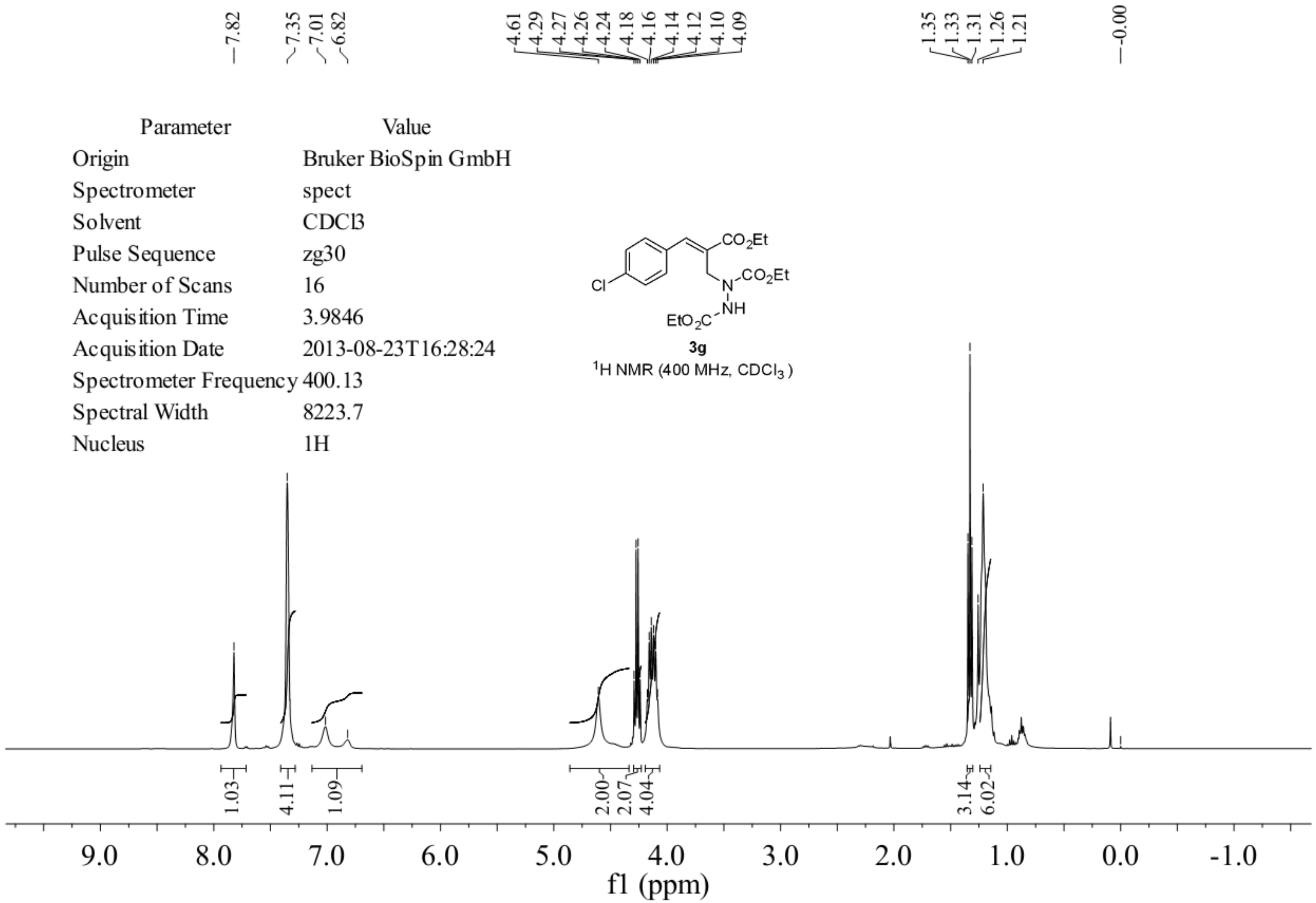
7.82
7.35
7.01
6.82
4.61
4.29
4.27
4.26
4.24
4.18
4.16
4.14
4.12
4.10
4.09
1.35
1.33
1.31
1.26
1.21
-0.00
Parameter Value
Origin Bruker BioSpin GmbH
Spectrometer spect
Solvent CDCl3
Pulse Sequence zg30
Number of Scans 16
Acquisition Time 3.9846
Acquisition Date 2013-08-23T16:28:24
Spectrometer Frequency 400.13
Spectral Width 8223.7
Nucleus 1H
CO2Et
Cl
N
CO2Et
NH
EtO2C
3g
1H NMR (400 MHz, CDCl3)
1.03
4.11
1.09
2.00
2.07
4.04
3.14
6.02
9.0
8.0
7.0
6.0
5.0
4.0
3.0
2.0
1.0
0.0
-1.0
f1 (ppm)

Parameter	Value
Origin	Bruker BioSpin GmbH
Spectrometer	spect
Solvent	CDCl3
Pulse Sequence	zgpg30
Number of Scans	564
Acquisition Time	1.3631
Acquisition Date	2013-08-20T19:19:58
Spectrometer Frequency	100.61
Spectral Width	24038.5
Nucleus	13C

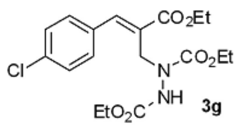

3g

^{13}C NMR (100 MHz, $CDCl_3$)

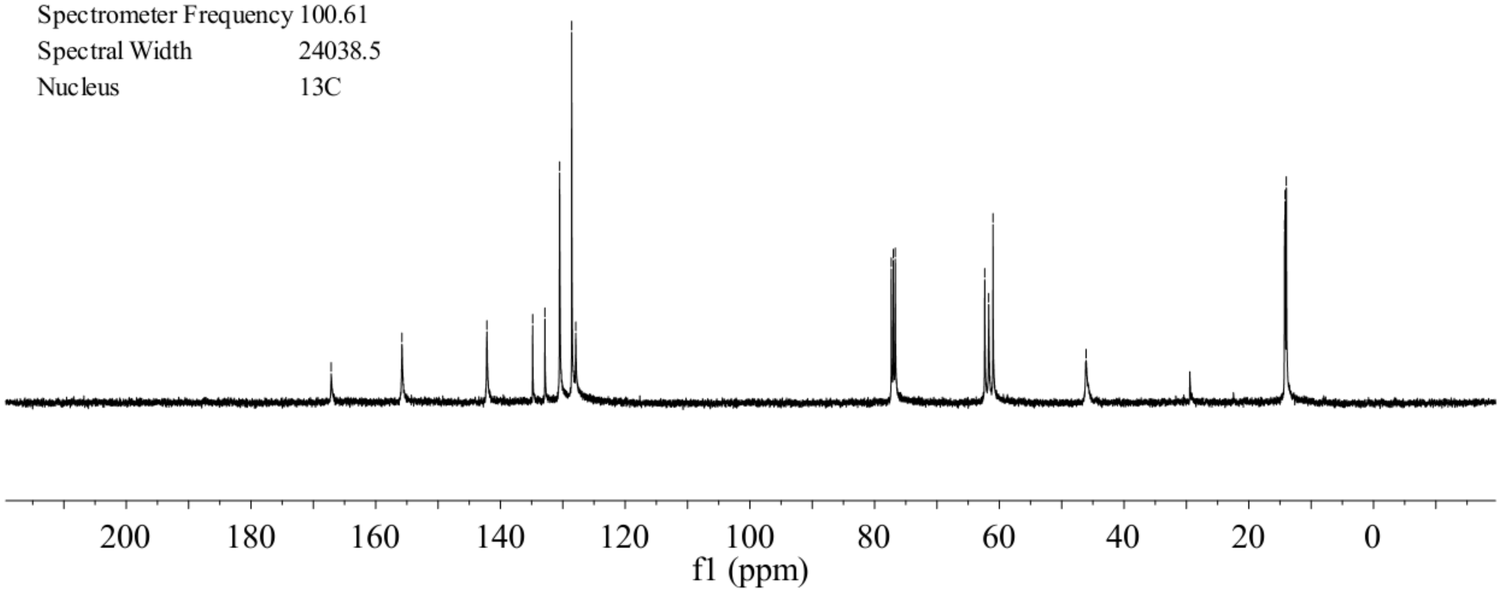

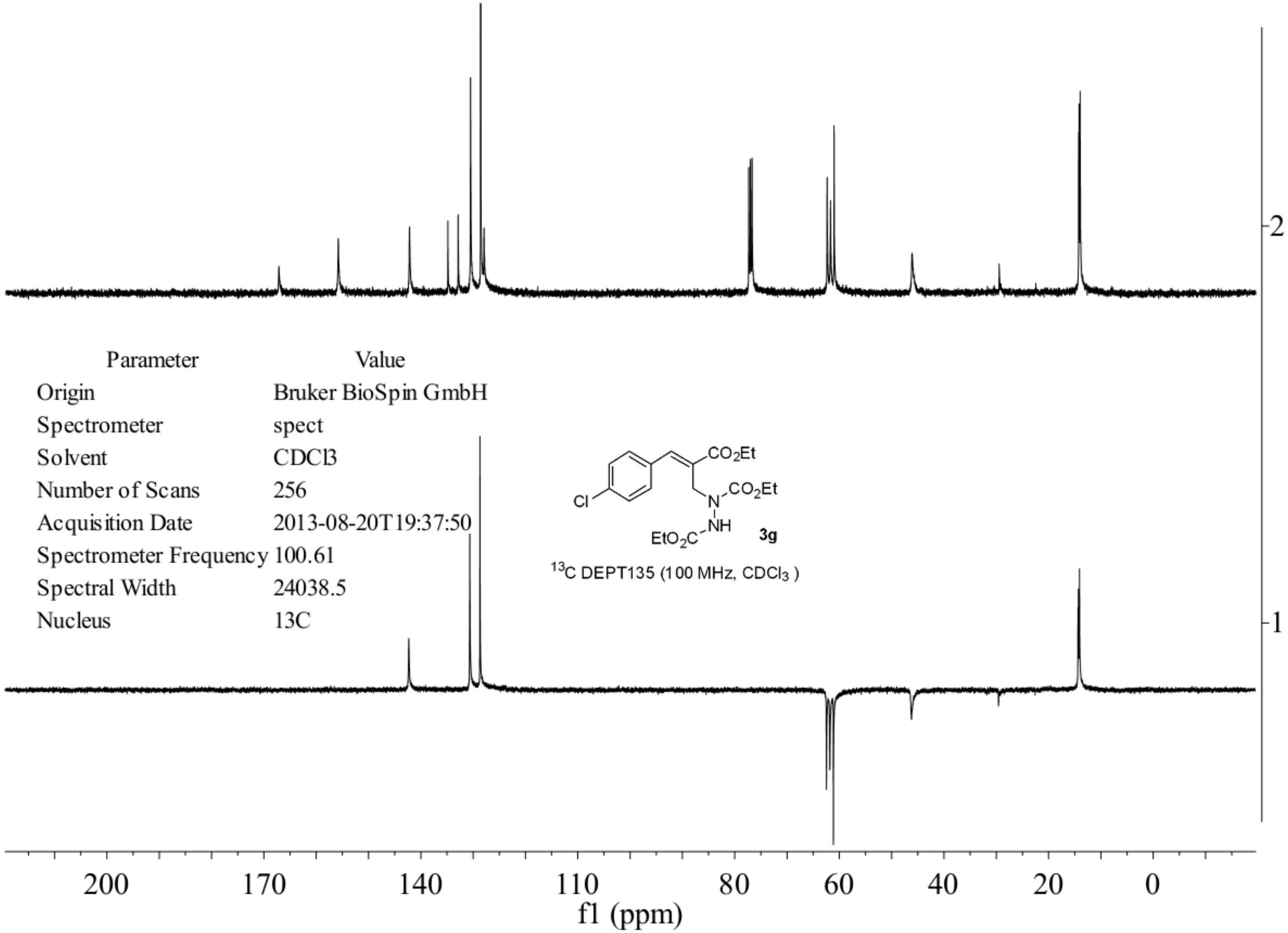

Parameter	Value
Origin	Bruker BioSpin GmbH
Spectrometer	spect
Solvent	CDCl3
Number of Scans	256
Acquisition Date	2013-08-20T19:37:50
Spectrometer Frequency	100.61
Spectral Width	24038.5
Nucleus	13C

3g

^{13}C DEPT135 (100 MHz, $CDCl_3$)

Parameter	Value
Origin	Bruker BioSpin GmbH
Spectrometer	spect
Solvent	CDCl3
Number of Scans	16
Acquisition Date	2013-08-23T16:52:26
Spectrometer Frequency	400.13
Spectral Width	8223.7
Nucleus	1H

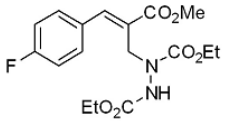

3h

^{1}H NMR(400 MHz, $CDCl_3$)

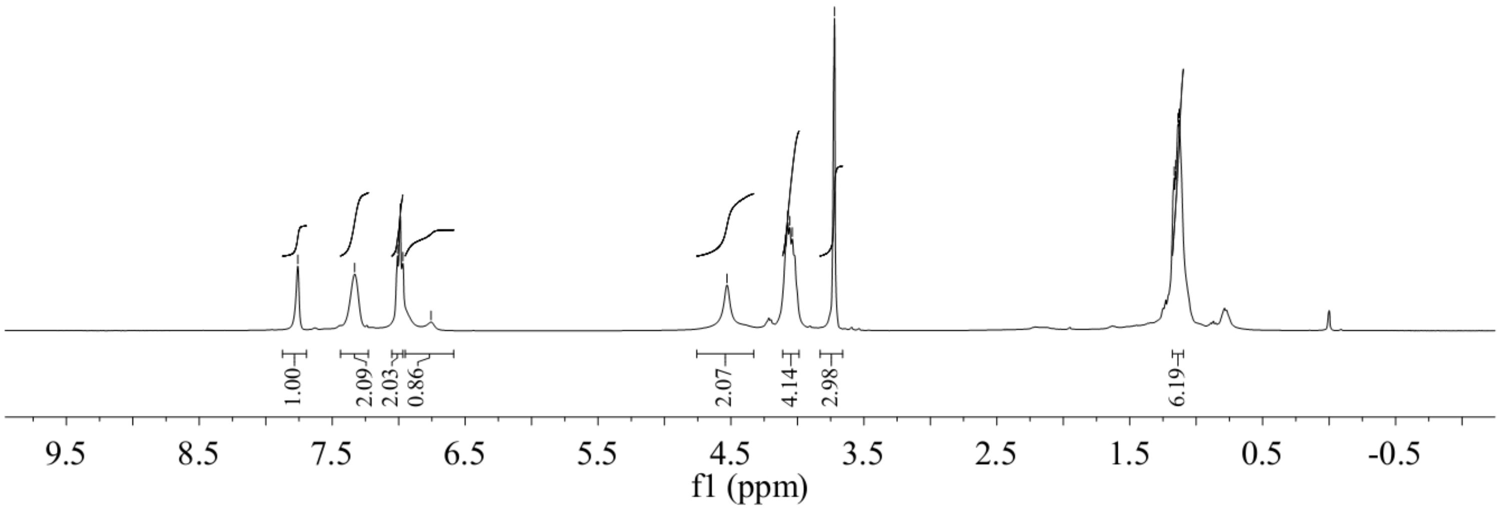

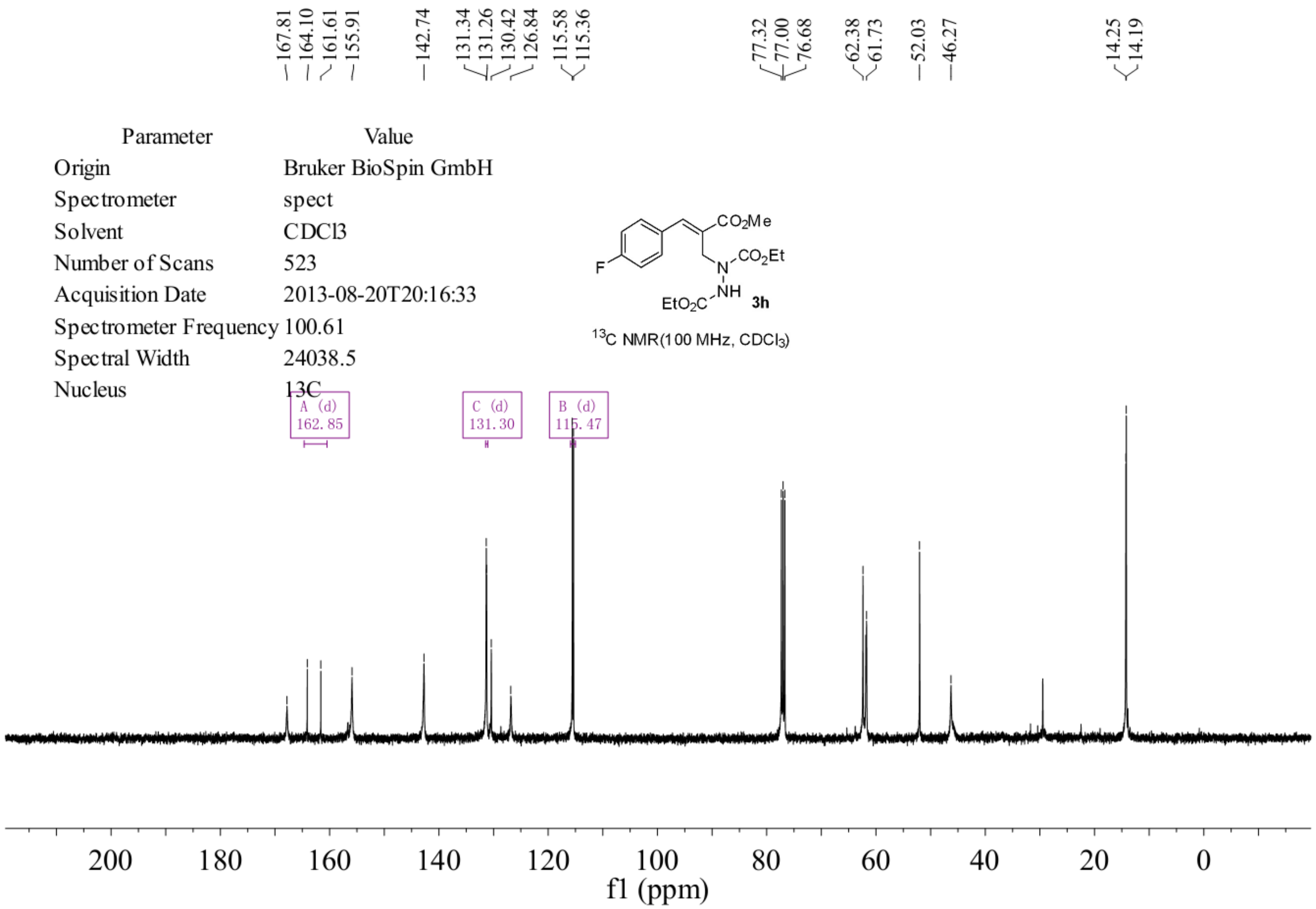

Parameter	Value
Origin	Bruker BioSpin GmbH
Spectrometer	spect
Solvent	CDCl3
Number of Scans	523
Acquisition Date	2013-08-20T20:16:33
Spectrometer Frequency	100.61
Spectral Width	24038.5
Nucleus	13C

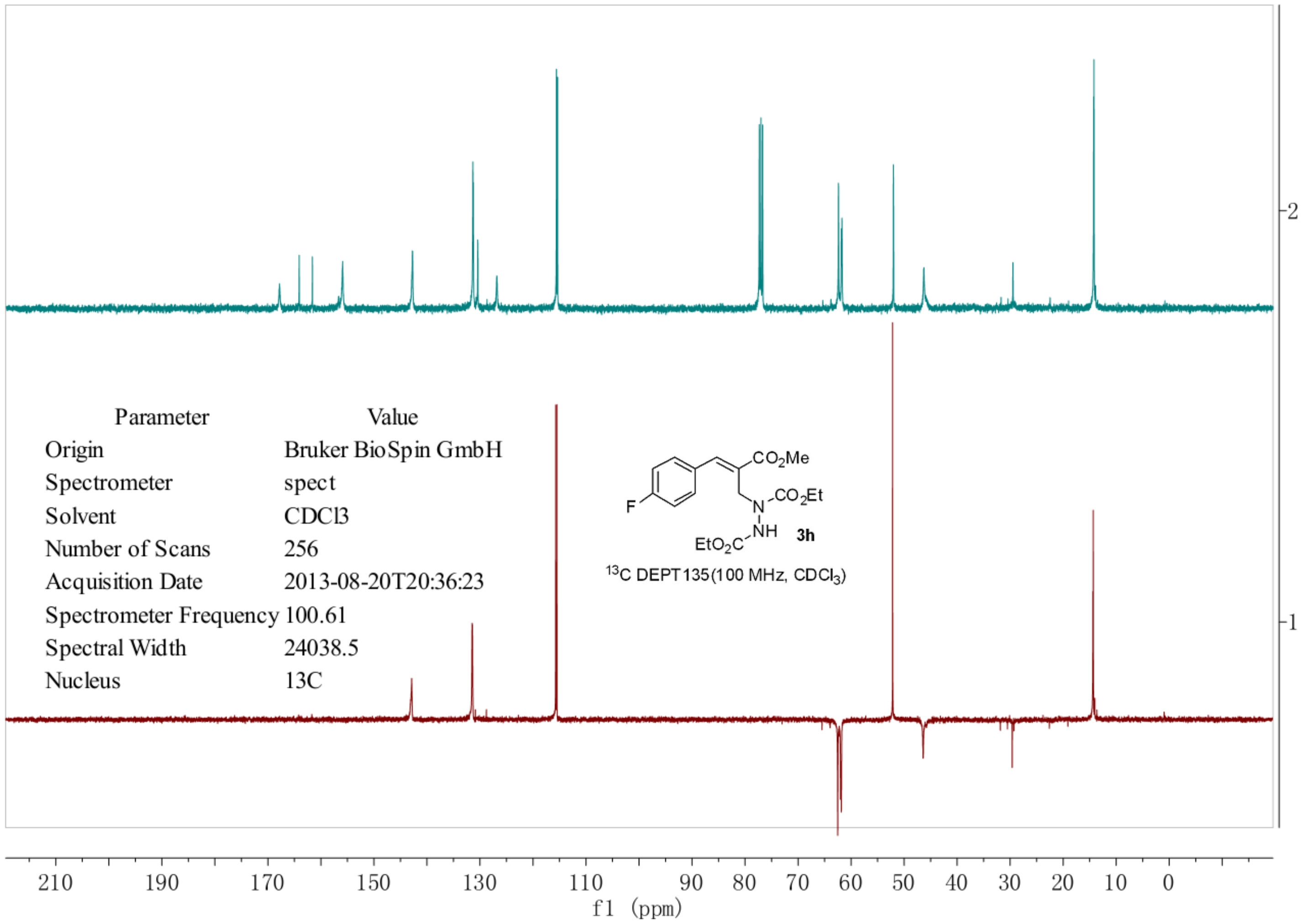

Parameter
Value
Origin
Bruker BioSpin GmbH
Spectrometer
spect
Solvent
CDCl3
Number of Scans
256
Acquisition Date
2013-08-20T20:36:23
Spectrometer Frequency
100.61
Spectral Width
24038.5
Nucleus
13C
CO2Me
CO2Et
F
N
NH
EtO2C
3h
13C DEPT135(100 MHz, CDCl3)
210
190
170
150
130
110
90
80
70
60
50
40
30
20
10
0
f1 (ppm)
2
1

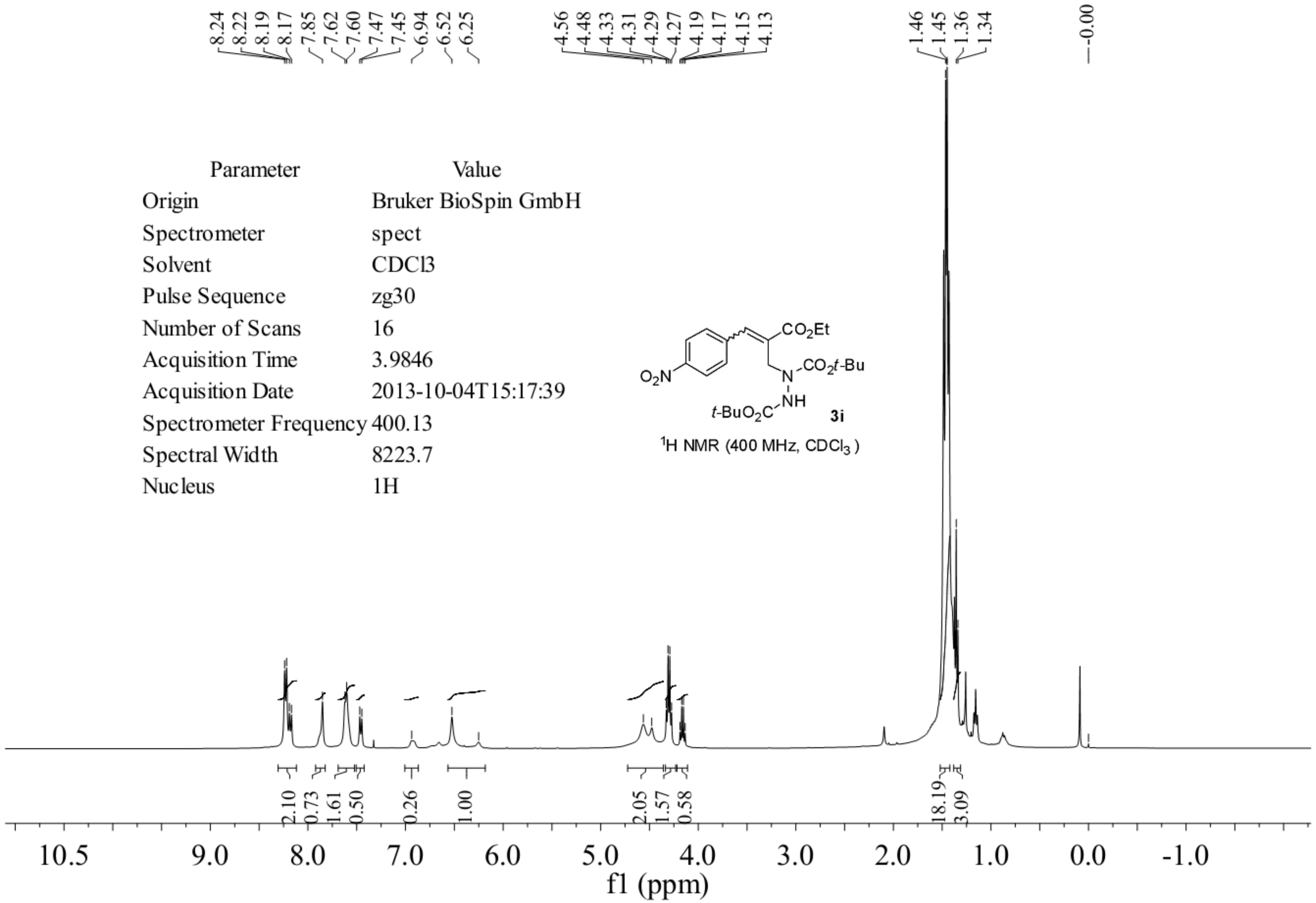
8.24
8.22
8.19
8.17
7.85
7.62
7.60
7.47
7.45
6.94
6.52
6.25
4.56
4.48
4.33
4.31
4.29
4.27
4.19
4.17
4.15
4.13
1.46
1.45
1.36
1.34
-0.00
Parameter Value
Origin Bruker BioSpin GmbH
Spectrometer spect
Solvent CDCl3
Pulse Sequence zg30
Number of Scans 16
Acquisition Time 3.9846
Acquisition Date 2013-10-04T15:17:39
Spectrometer Frequency 400.13
Spectral Width 8223.7
Nucleus 1H
CO2Et
O2N
N
CO2t-Bu
NH
t-BuO2C
3i
1H NMR (400 MHz, CDCl3)
2.10
0.73
1.61
0.50
0.26
1.00
2.05
1.57
0.58
18.19
3.09
10.5
9.0
8.0
7.0
6.0
5.0
4.0
3.0
2.0
1.0
0.0
-1.0
f1 (ppm)

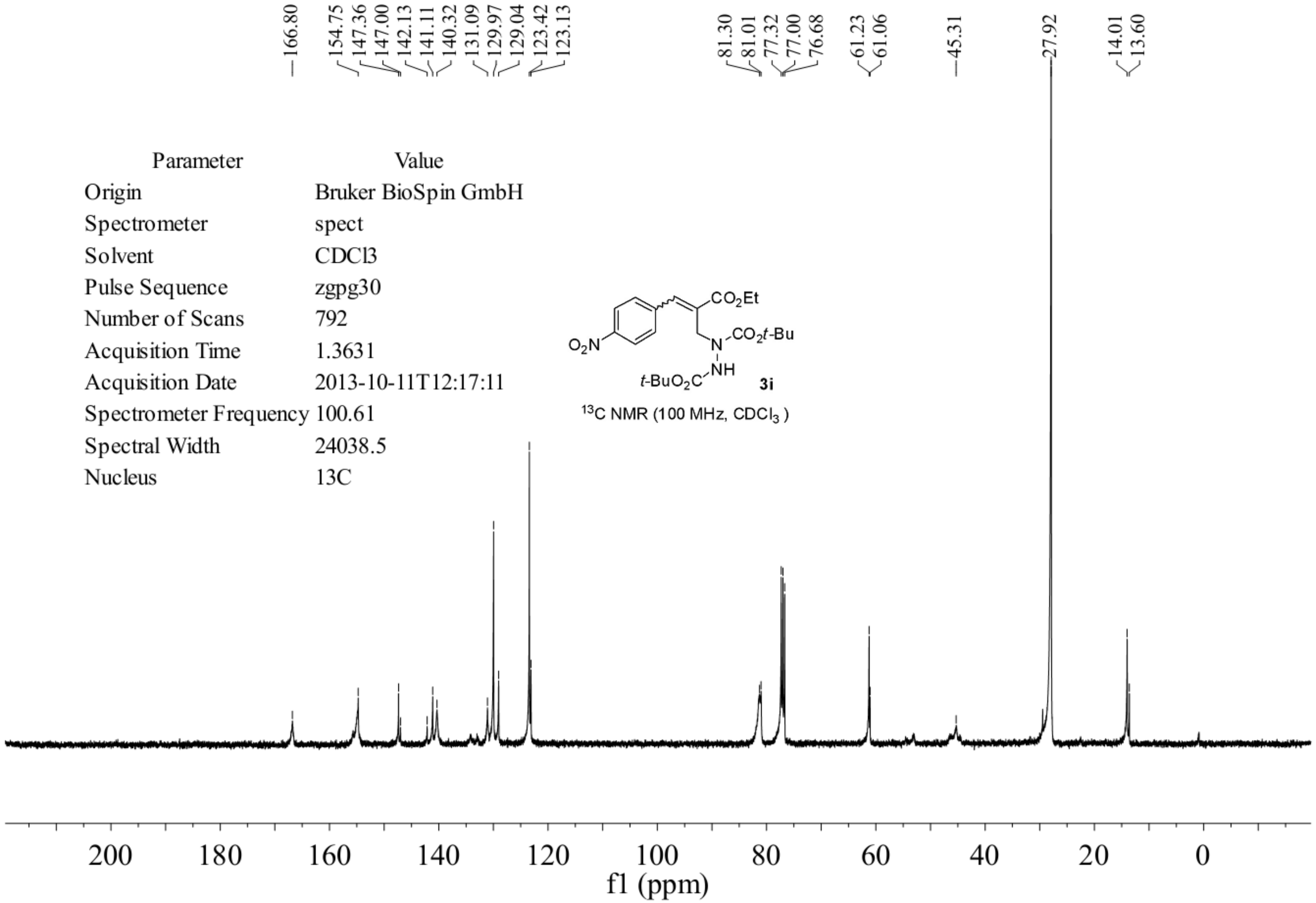

Parameter	Value
Origin	Bruker BioSpin GmbH
Spectrometer	spect
Solvent	CDCl3
Pulse Sequence	zgpg30
Number of Scans	792
Acquisition Time	1.3631
Acquisition Date	2013-10-11T12:17:11
Spectrometer Frequency	100.61
Spectral Width	24038.5
Nucleus	13C

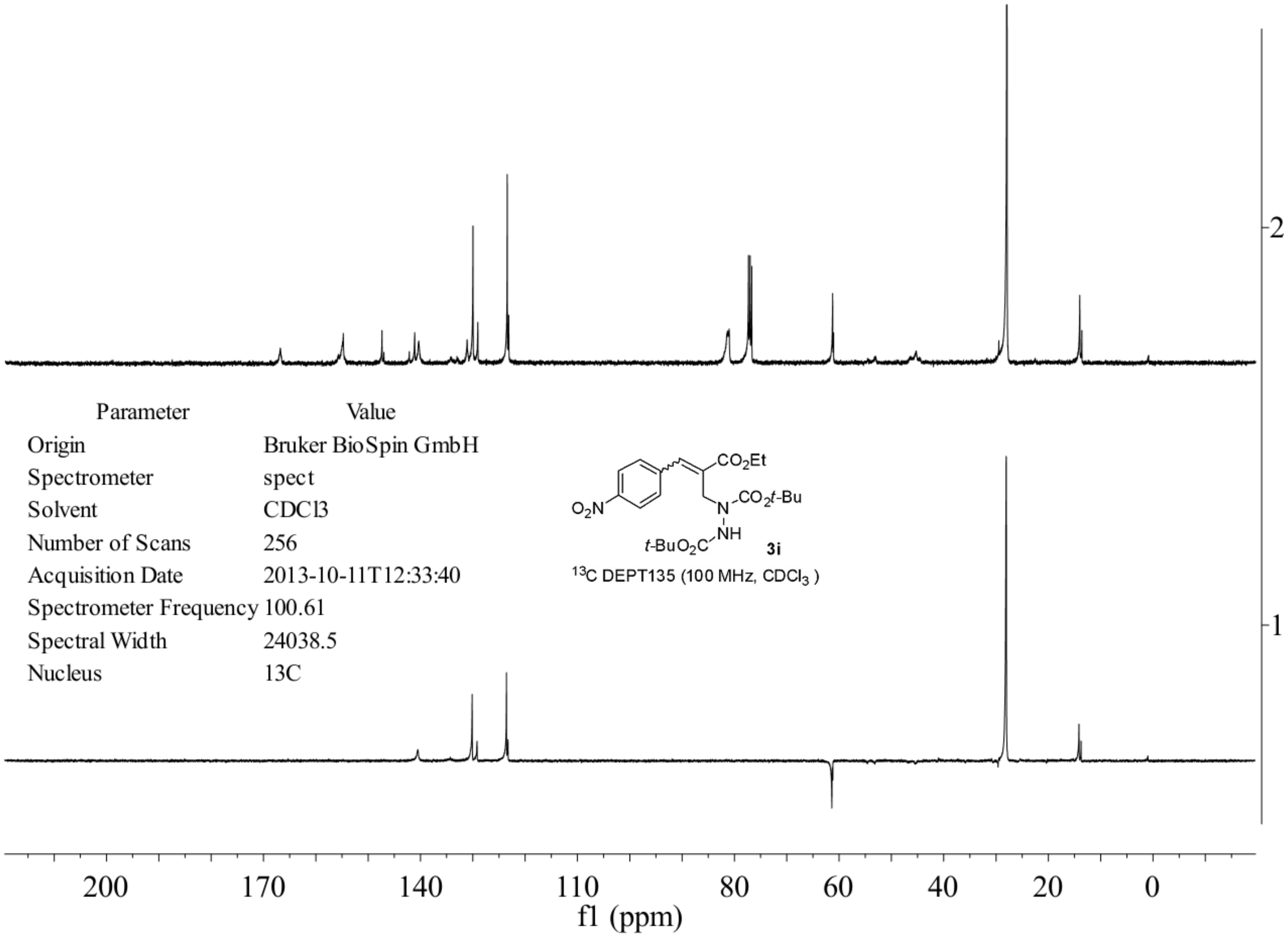

Parameter
Value
Origin
Bruker BioSpin GmbH
Spectrometer
spect
Solvent
CDCl3
Number of Scans
256
Acquisition Date
2013-10-11T12:33:40
Spectrometer Frequency
100.61
Spectral Width
24038.5
Nucleus
13C
CO2Et
O2N
CO2t-Bu
NH
t-BuO2C
3i
13C DEPT135 (100 MHz, CDCl3)
2
1
200
170
140
110
80
60
40
20
0
f1 (ppm)

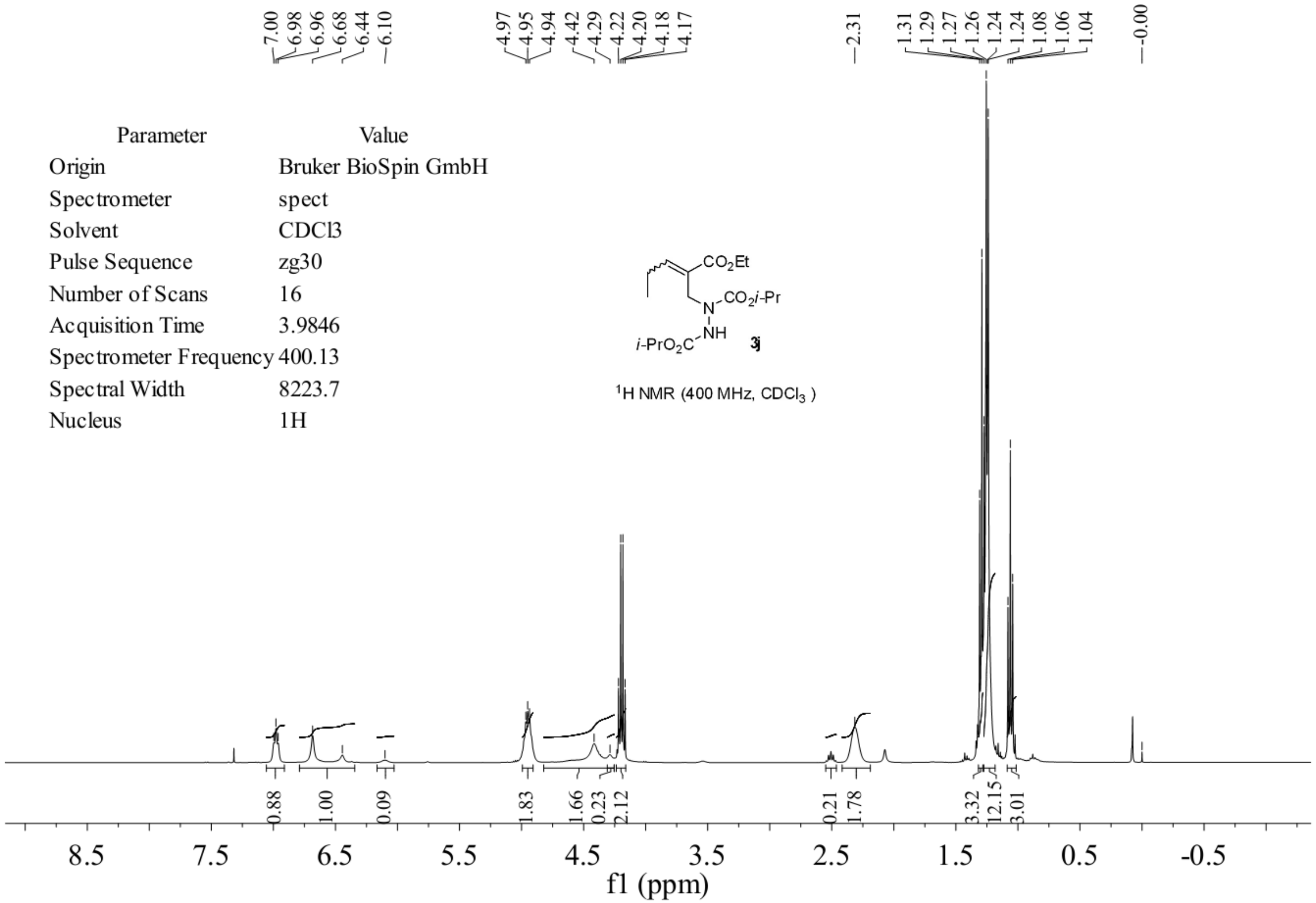
7.00 6.98 6.96 6.68 6.44 6.10
4.97 4.95 4.94 4.42 4.29 4.22 4.20 4.18 4.17
2.31
1.31 1.29 1.27 1.26 1.24 1.24 1.08 1.06 1.04
-0.00
Parameter Value
Origin Bruker BioSpin GmbH
Spectrometer spect
Solvent CDCl3
Pulse Sequence zg30
Number of Scans 16
Acquisition Time 3.9846
Spectrometer Frequency 400.13
Spectral Width 8223.7
Nucleus 1H
CO2Et
N CO2i-Pr
NH
i-PrO2C
3j
1H NMR (400 MHz, CDCl3)
0.88 1.00 0.09 1.83 1.66 0.23 2.12 0.21 1.78 3.32 12.15 3.01
8.5 7.5 6.5 5.5 4.5 3.5 2.5 1.5 0.5 -0.5
f1 (ppm)

Parameter	Value
Origin	Bruker BioSpin GmbH
Spectrometer	spect
Solvent	CDCl3
Pulse Sequence	zgpg30
Number of Scans	764
Acquisition Time	1.3631
Acquisition Date	2013-09-03T12:27:16
Spectrometer Frequency	100.61
Spectral Width	24038.5
Nucleus	13C

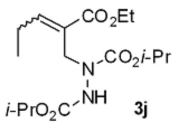

3j

^{13}C NMR (100 MHz, $CDCl_3$)

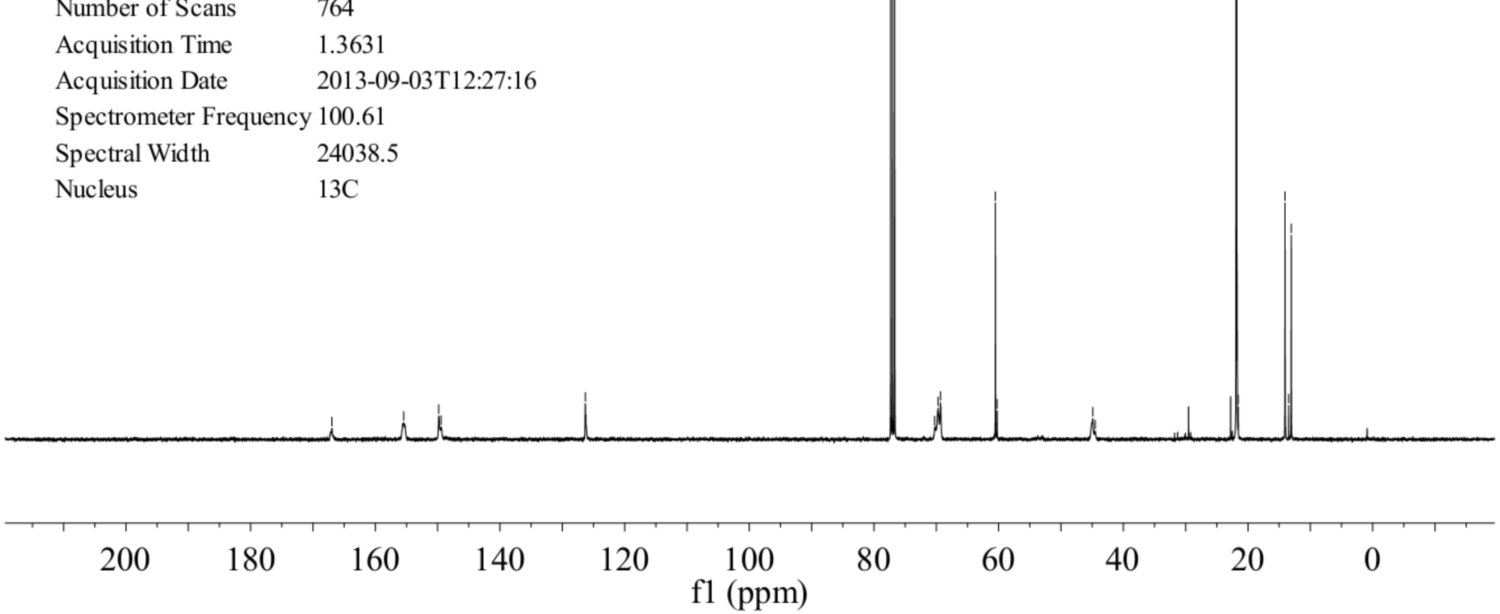

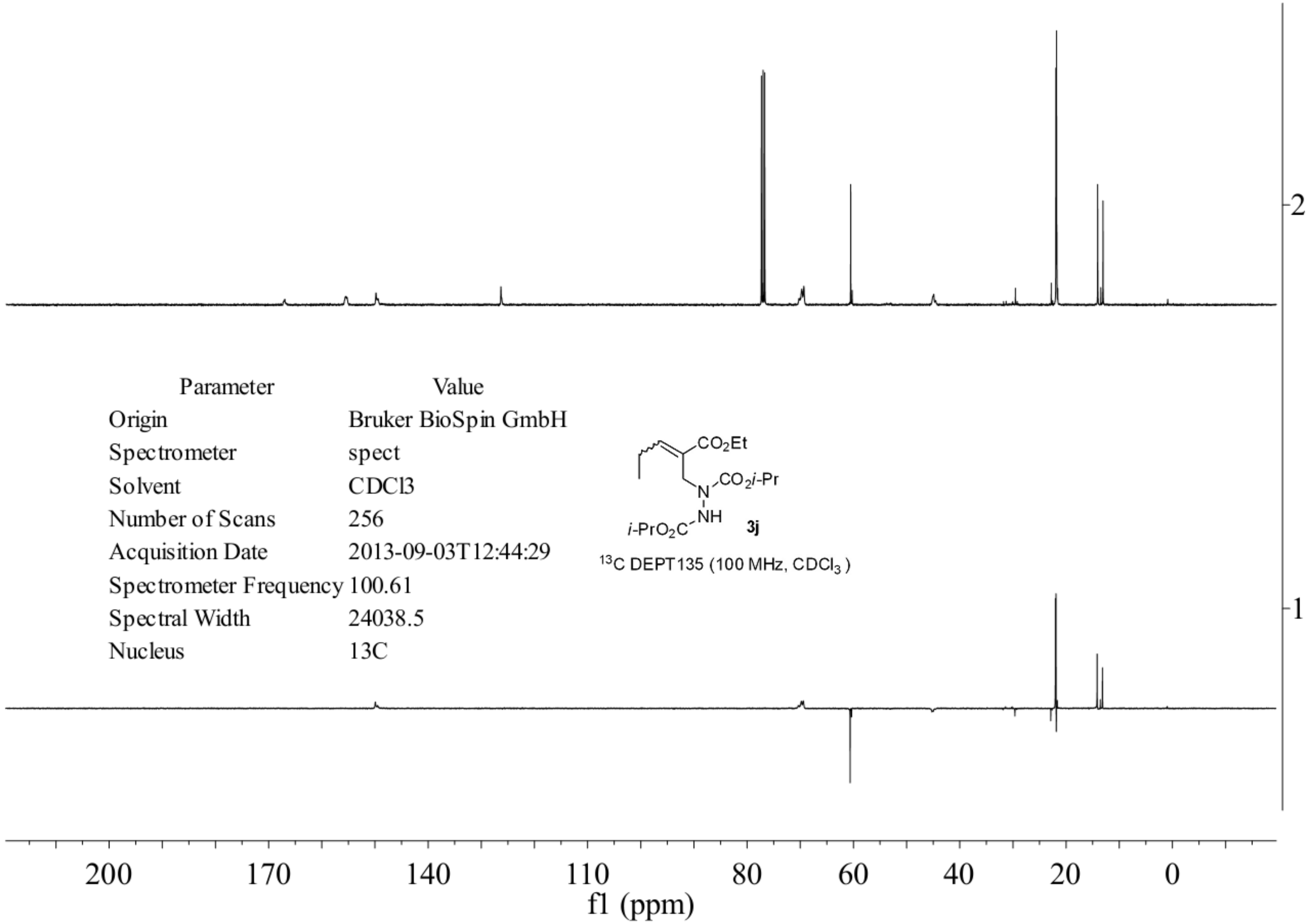
Parameter Value
Origin Bruker BioSpin GmbH
Spectrometer spect
Solvent CDCl3
Number of Scans 256
Acquisition Date 2013-09-03T12:44:29
Spectrometer Frequency 100.61
Spectral Width 24038.5
Nucleus 13C
CO2Et
CO2i-Pr
N
NH
i-PrO2C
3j
13C DEPT135 (100 MHz, CDCl3)
2
1
200 170 140 110 80 60 40 20 0
f1 (ppm)

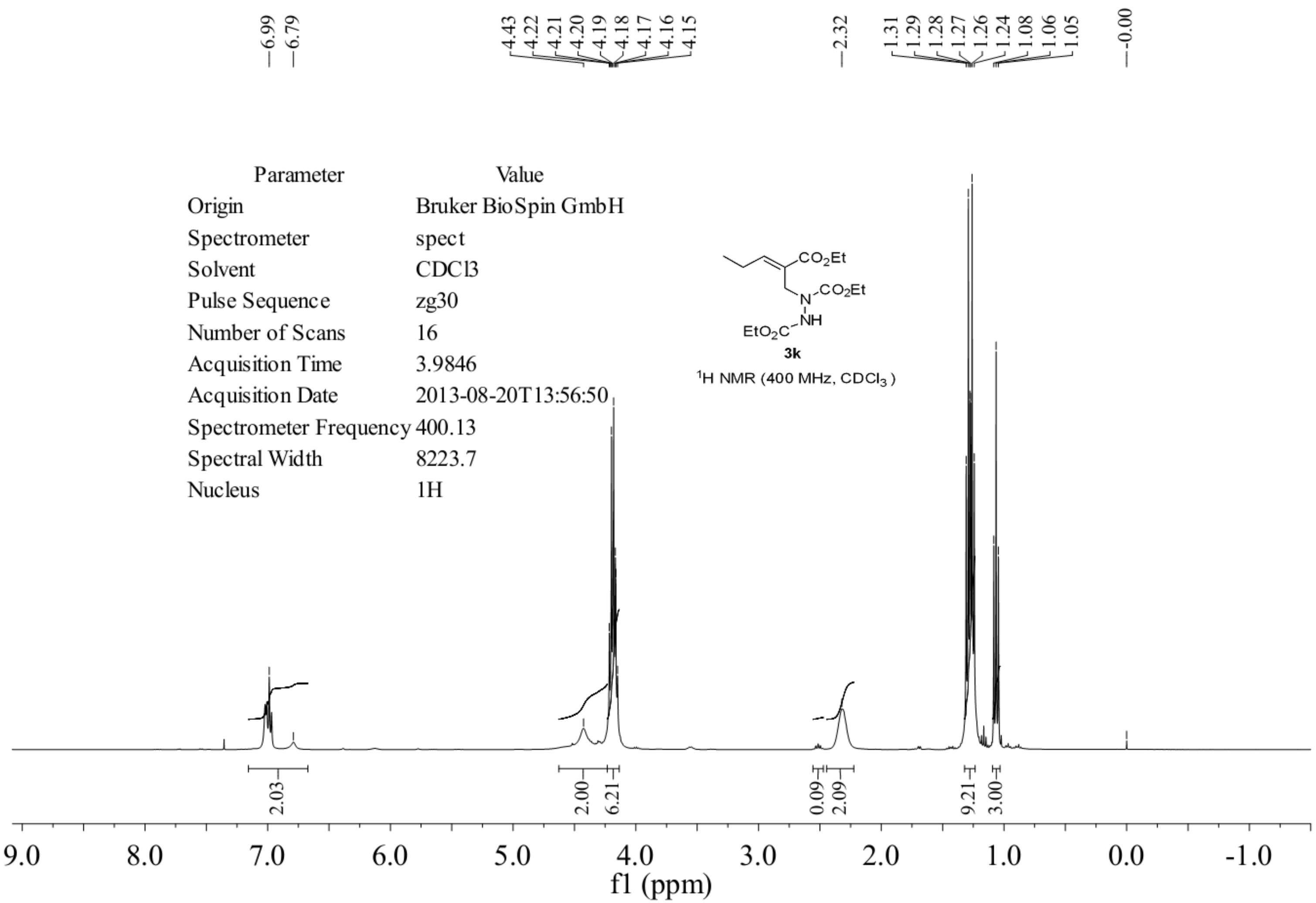
6.99
6.79
4.43
4.22
4.21
4.20
4.19
4.18
4.17
4.16
4.15
2.32
1.31
1.29
1.28
1.27
1.26
1.24
1.08
1.06
1.05
-0.00
Parameter
Value
Origin
Bruker BioSpin GmbH
Spectrometer
spect
Solvent
CDCl3
Pulse Sequence
zg30
Number of Scans
16
Acquisition Time
3.9846
Acquisition Date
2013-08-20T13:56:50
Spectrometer Frequency
400.13
Spectral Width
8223.7
Nucleus
1H
CO2Et
N
CO2Et
NH
EtO2C
3k
1H NMR (400 MHz, CDCl3)
2.03
2.00
6.21
0.09
2.09
9.21
3.00
9.0
8.0
7.0
6.0
5.0
4.0
3.0
2.0
1.0
0.0
-1.0
f1 (ppm)

Parameter	Value
Origin	Bruker BioSpin GmbH
Spectrometer	spect
Solvent	CDCl3
Pulse Sequence	zgpg30
Number of Scans	527
Acquisition Time	1.3631
Acquisition Date	2013-08-20T14:30:20
Spectrometer Frequency	100.61
Spectral Width	24038.5
Nucleus	13C

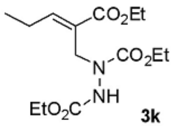

3k

^{13}C NMR (100 MHz, $CDCl_3$)

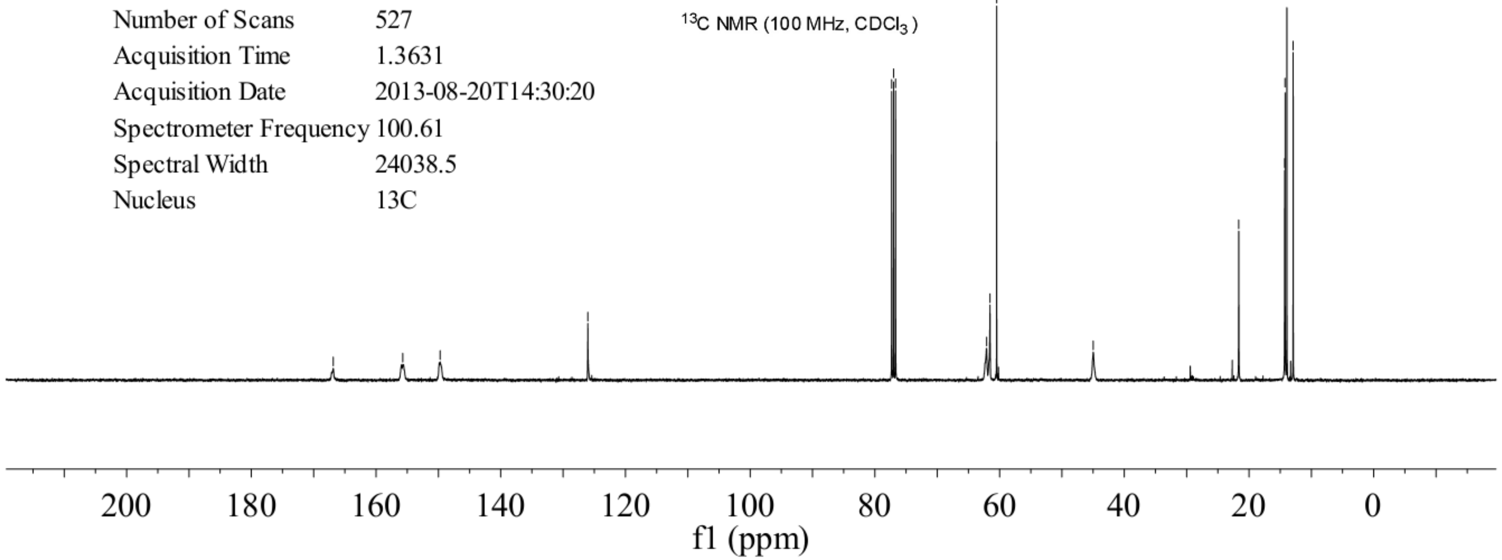

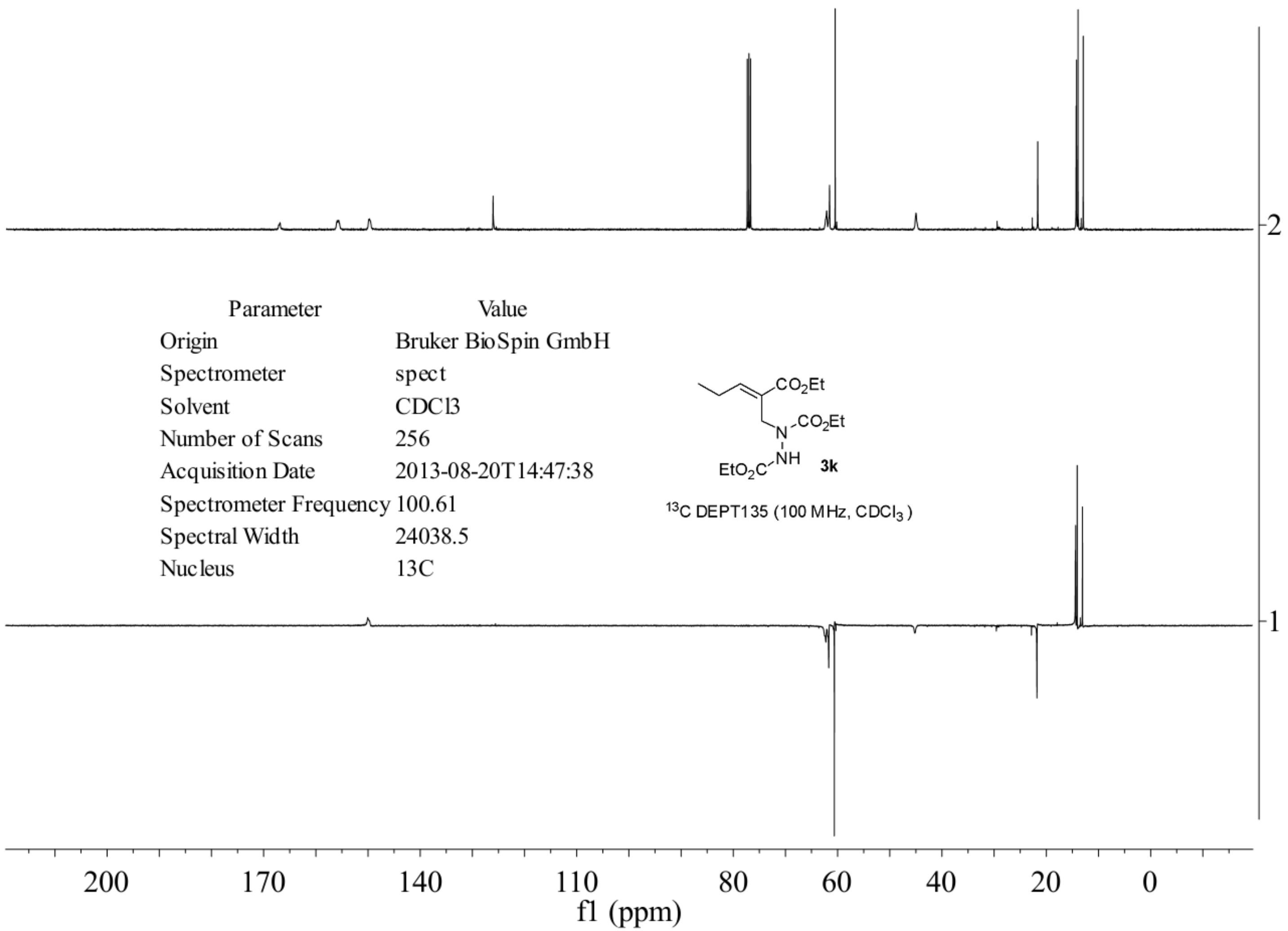

Parameter	Value
Origin	Bruker BioSpin GmbH
Spectrometer	spect
Solvent	CDCl3
Number of Scans	256
Acquisition Date	2013-08-20T14:47:38
Spectrometer Frequency	100.61
Spectral Width	24038.5
Nucleus	13C

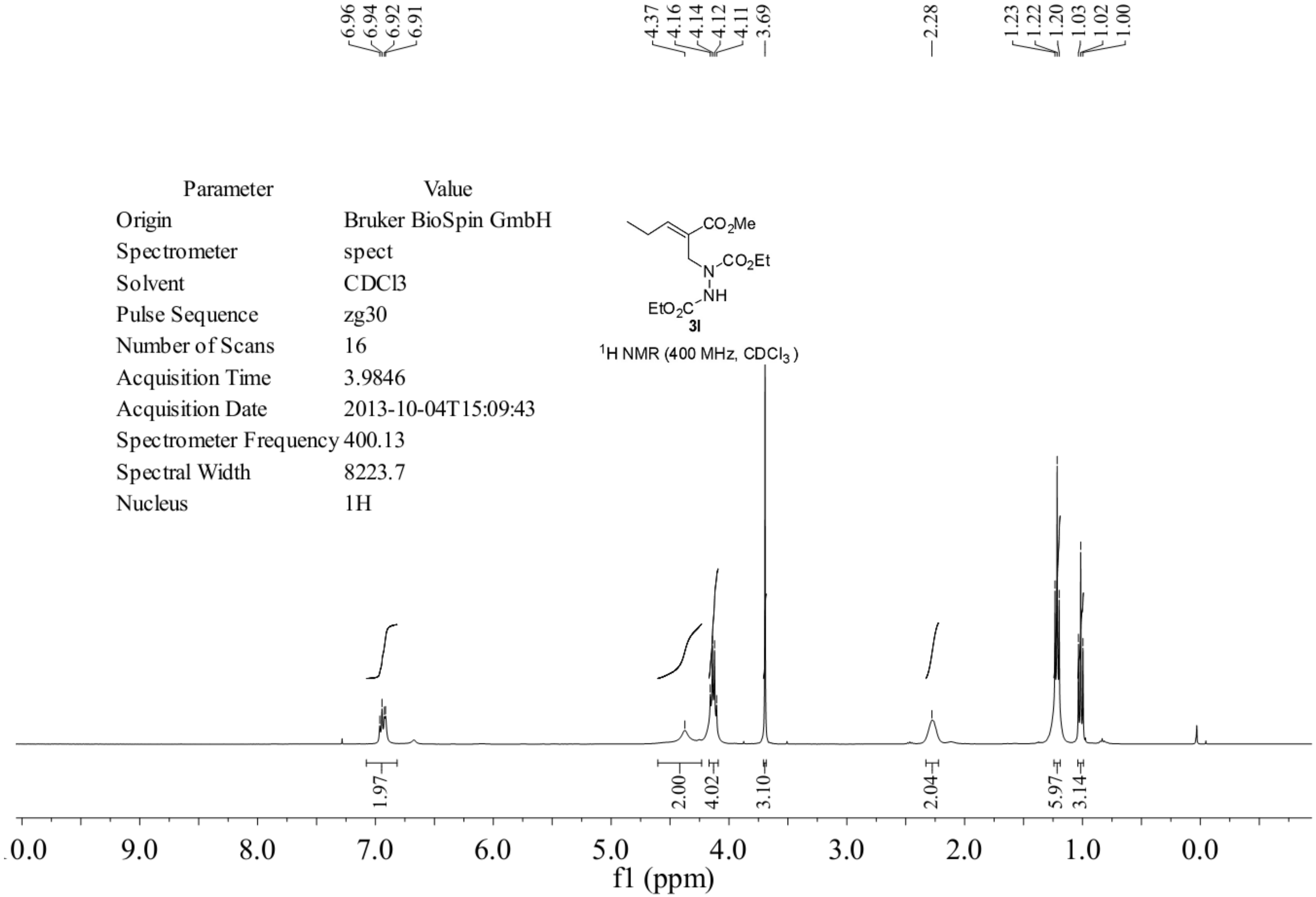

Parameter	Value
Origin	Bruker BioSpin GmbH
Spectrometer	spect
Solvent	CDCl3
Pulse Sequence	zg30
Number of Scans	16
Acquisition Time	3.9846
Acquisition Date	2013-10-04T15:09:43
Spectrometer Frequency	400.13
Spectral Width	8223.7
Nucleus	1H

Parameter	Value
Origin	Bruker BioSpin GmbH
Spectrometer	spect
Solvent	CDCl3
Pulse Sequence	zgpg30
Number of Scans	837
Acquisition Time	1.3631
Acquisition Date	2013-10-11T08:10:36
Spectrometer Frequency	100.61
Spectral Width	24038.5
Nucleus	13C

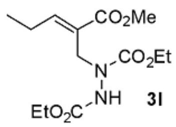

3l

^{13}C NMR (100 MHz, $CDCl_3$)

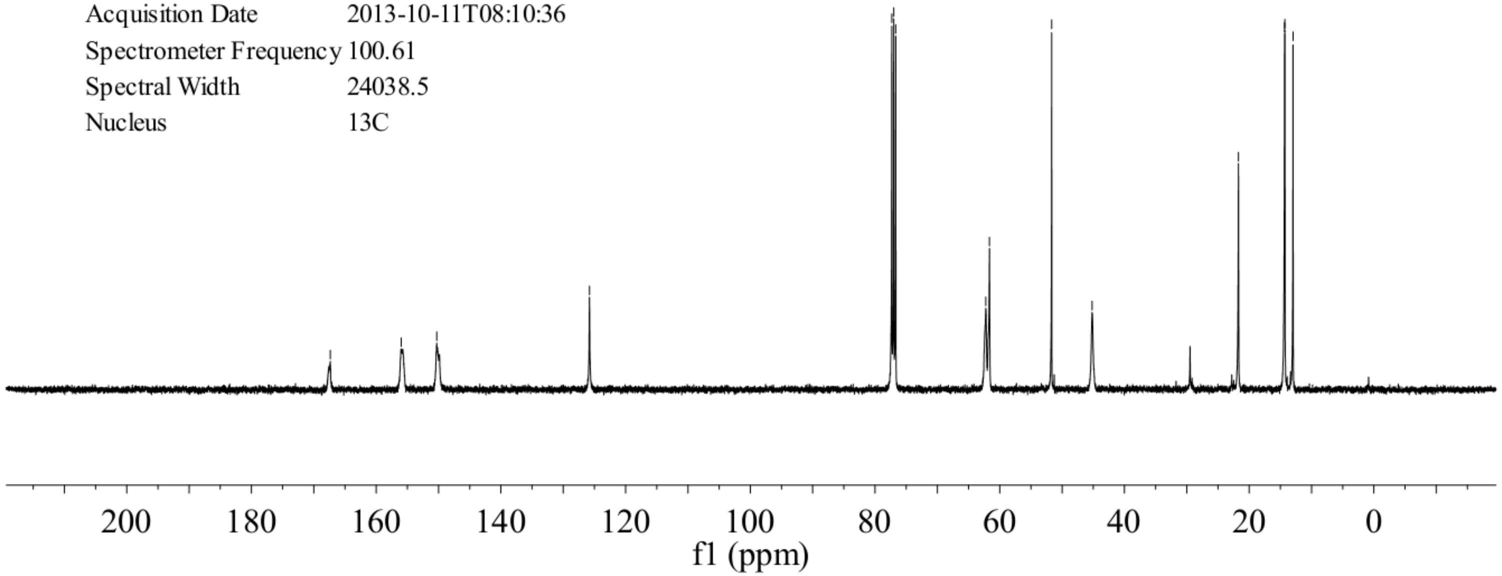

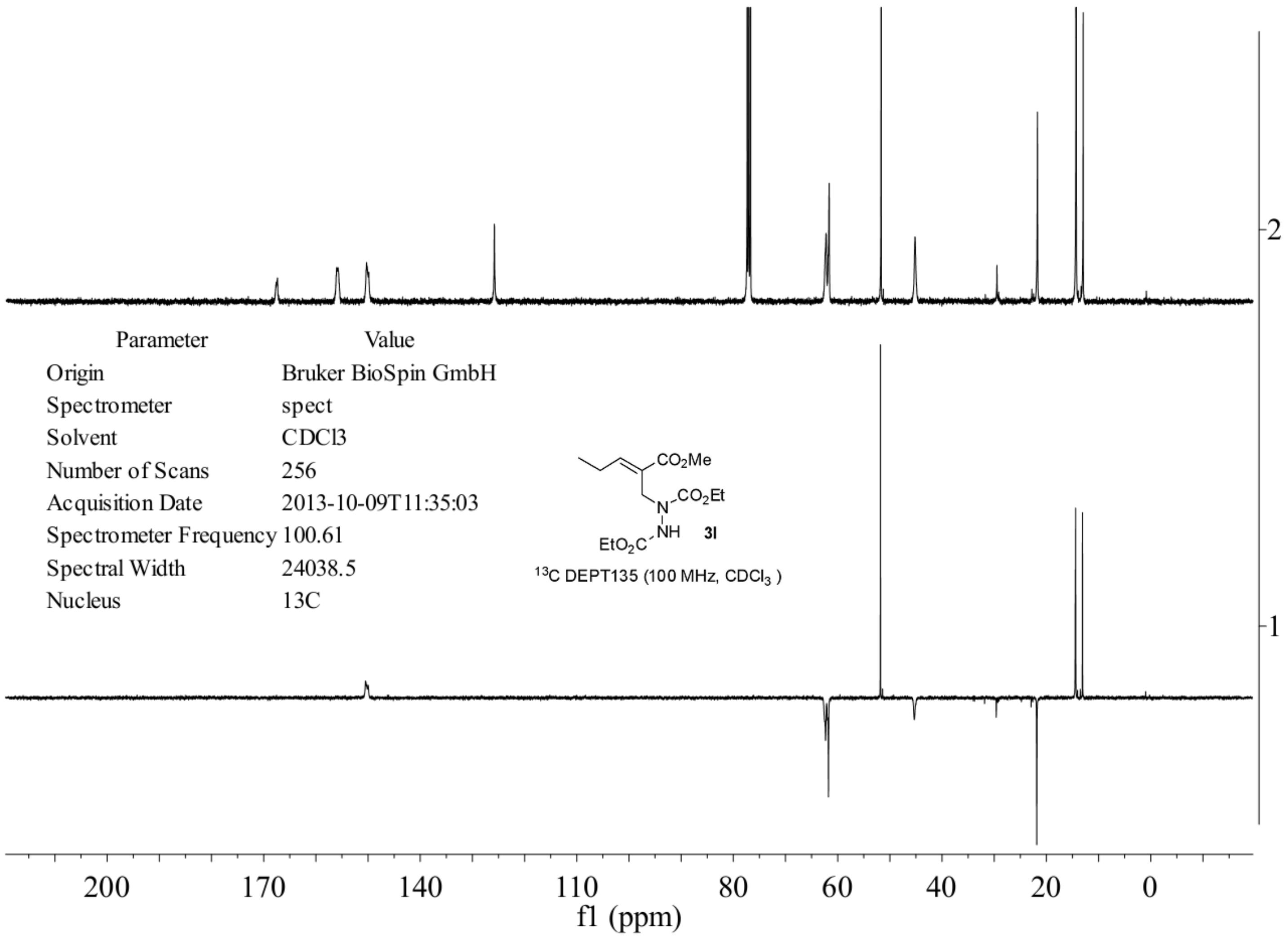

Parameter
Value
Origin
Bruker BioSpin GmbH
Spectrometer
spect
Solvent
CDCl3
Number of Scans
256
Acquisition Date
2013-10-09T11:35:03
Spectrometer Frequency
100.61
Spectral Width
24038.5
Nucleus
13C
CO2Me
CO2Et
N
NH
EtO2C
3l
13C DEPT135 (100 MHz, CDCl3)
2
1
200
170
140
110
80
60
40
20
0
f1 (ppm)

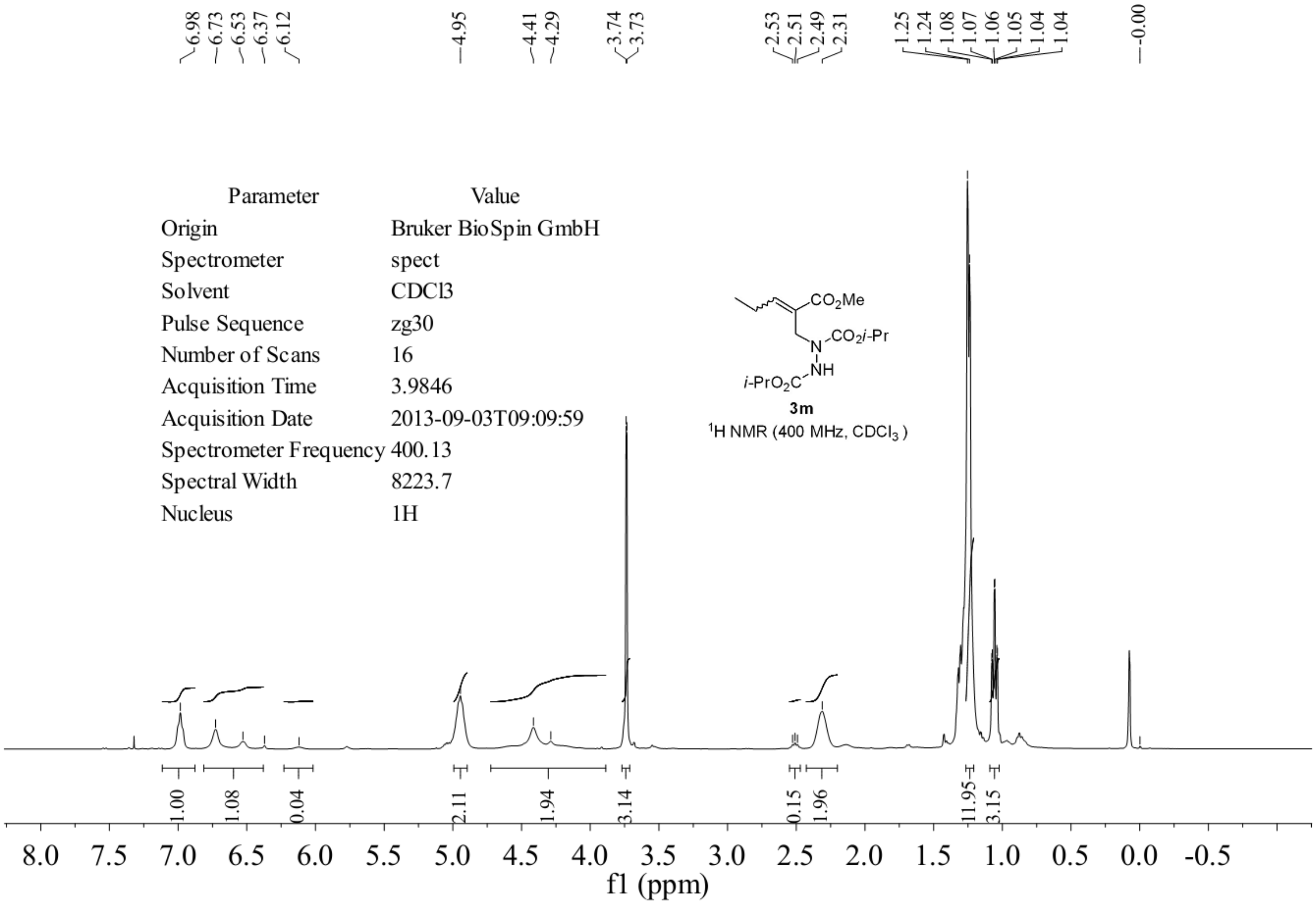
6.98
6.73
6.53
6.37
6.12
4.95
4.41
4.29
3.74
3.73
2.53
2.51
2.49
2.31
1.25
1.24
1.08
1.07
1.06
1.05
1.04
1.04
-0.00
Parameter
Value
Origin
Bruker BioSpin GmbH
Spectrometer
spect
Solvent
CDCl3
Pulse Sequence
zg30
Number of Scans
16
Acquisition Time
3.9846
Acquisition Date
2013-09-03T09:09:59
Spectrometer Frequency
400.13
Spectral Width
8223.7
Nucleus
1H
CO2Me
CO2i-Pr
N
NH
i-PrO2C
3m
1H NMR (400 MHz, CDCl3)
1.00
1.08
0.04
2.11
1.94
3.14
0.15
1.96
11.95
3.15
8.0 7.5 7.0 6.5 6.0 5.5 5.0 4.5 4.0 3.5 3.0 2.5 2.0 1.5 1.0 0.5 0.0 -0.5
f1 (ppm)

Parameter	Value
Origin	Bruker BioSpin GmbH
Spectrometer	spect
Solvent	CDCl3
Pulse Sequence	zgpg30
Number of Scans	876
Acquisition Time	1.3631
Acquisition Date	2013-09-03T10:04:34
Spectrometer Frequency	100.61
Spectral Width	24038.5
Nucleus	13C

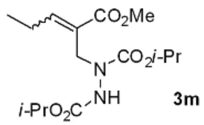

3m

^{13}C NMR (100 MHz, $CDCl_3$)

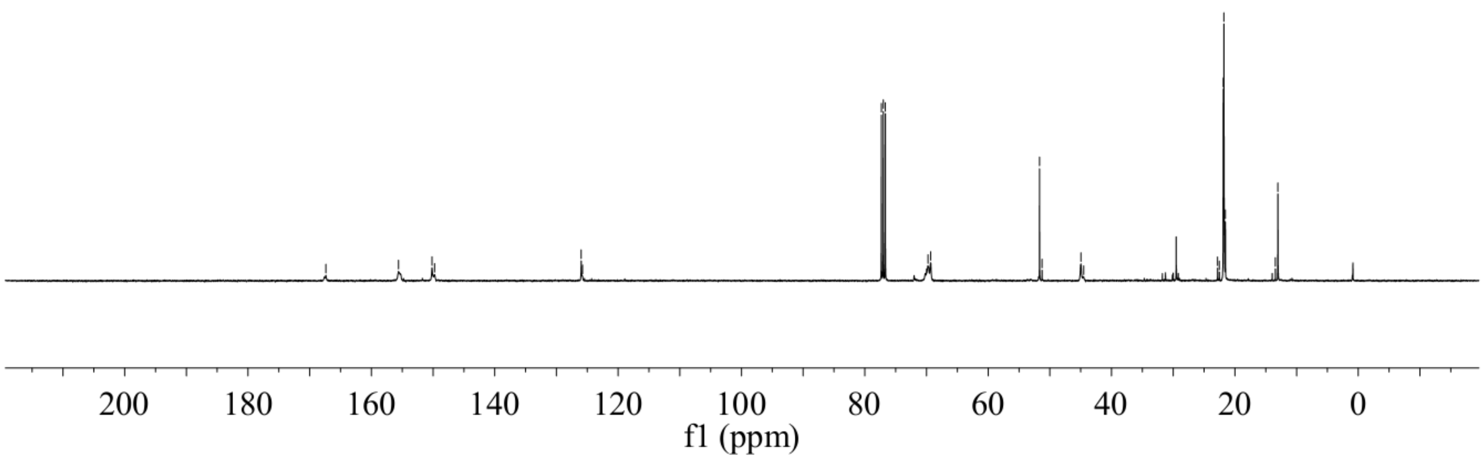

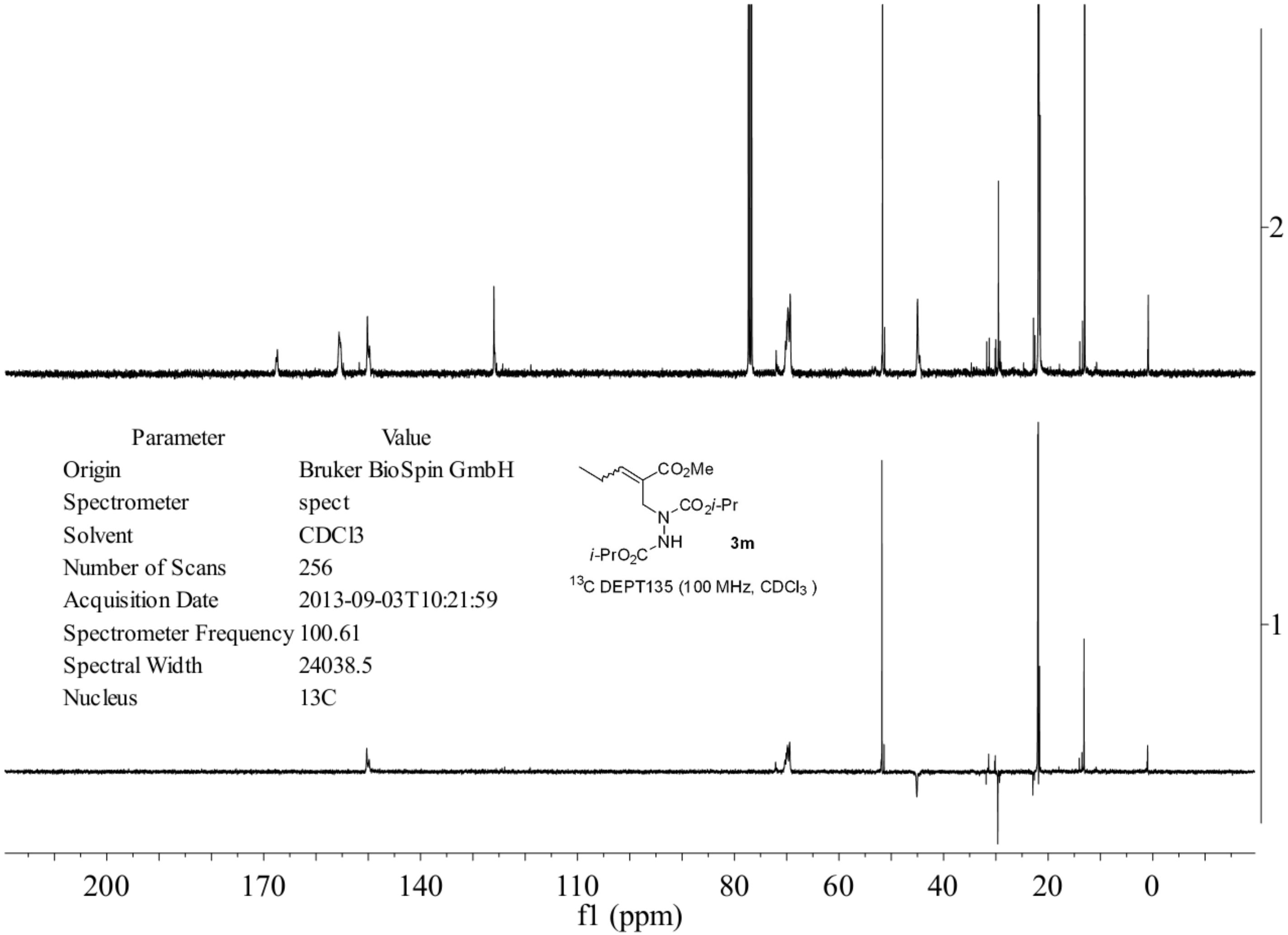
Parameter Value
Origin Bruker BioSpin GmbH
Spectrometer spect
Solvent CDCl3
Number of Scans 256
Acquisition Date 2013-09-03T10:21:59
Spectrometer Frequency 100.61
Spectral Width 24038.5
Nucleus 13C
CO2Me
CO2i-Pr
N
NH
i-PrO2C
3m
13C DEPT135 (100 MHz, CDCl3)
2
1
200
170
140
110
80
60
40
20
0
f1 (ppm)

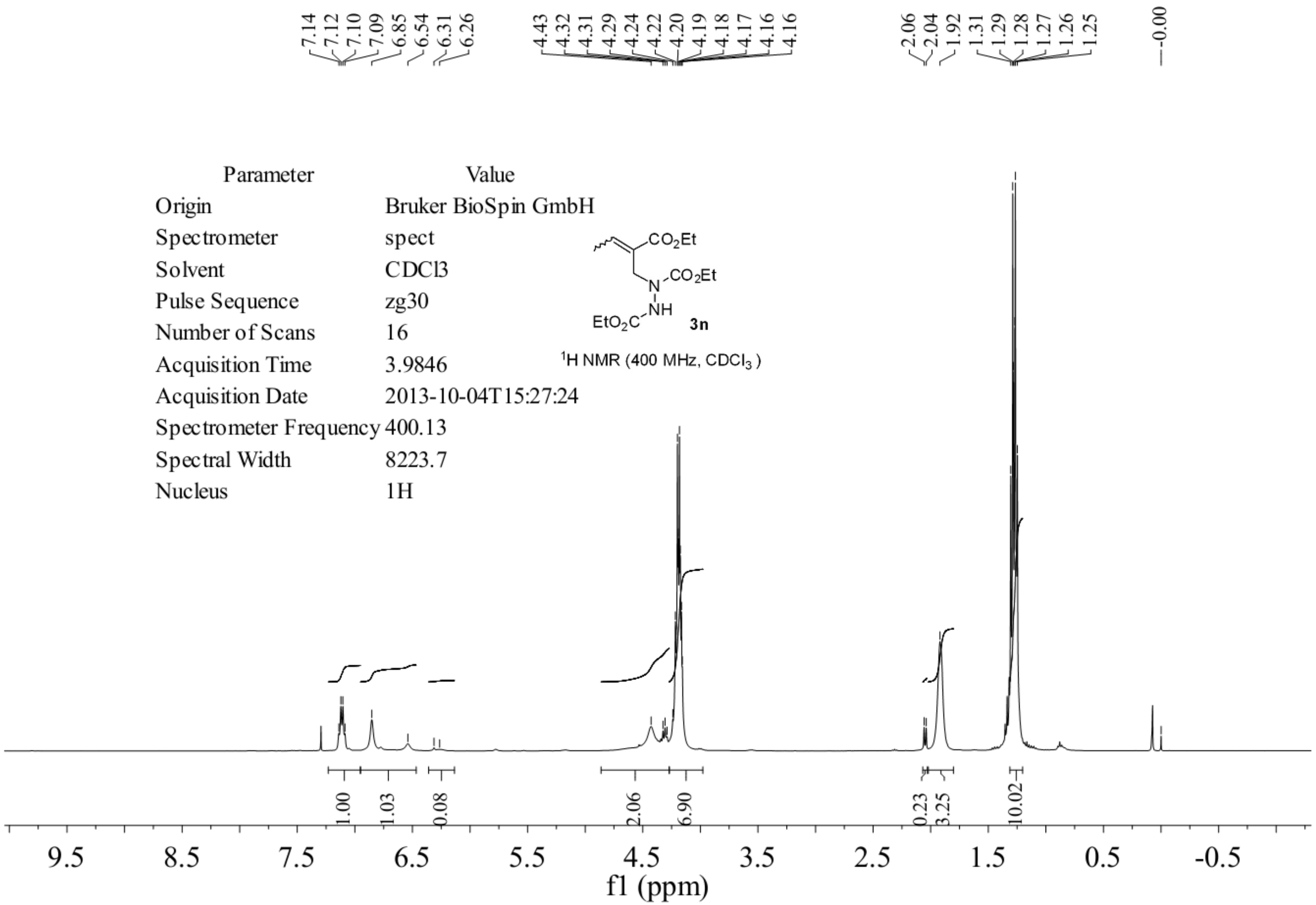

Parameter	Value
Origin	Bruker BioSpin GmbH
Spectrometer	spect
Solvent	CDCl3
Pulse Sequence	zg30
Number of Scans	16
Acquisition Time	3.9846
Acquisition Date	2013-10-04T15:27:24
Spectrometer Frequency	400.13
Spectral Width	8223.7
Nucleus	1H

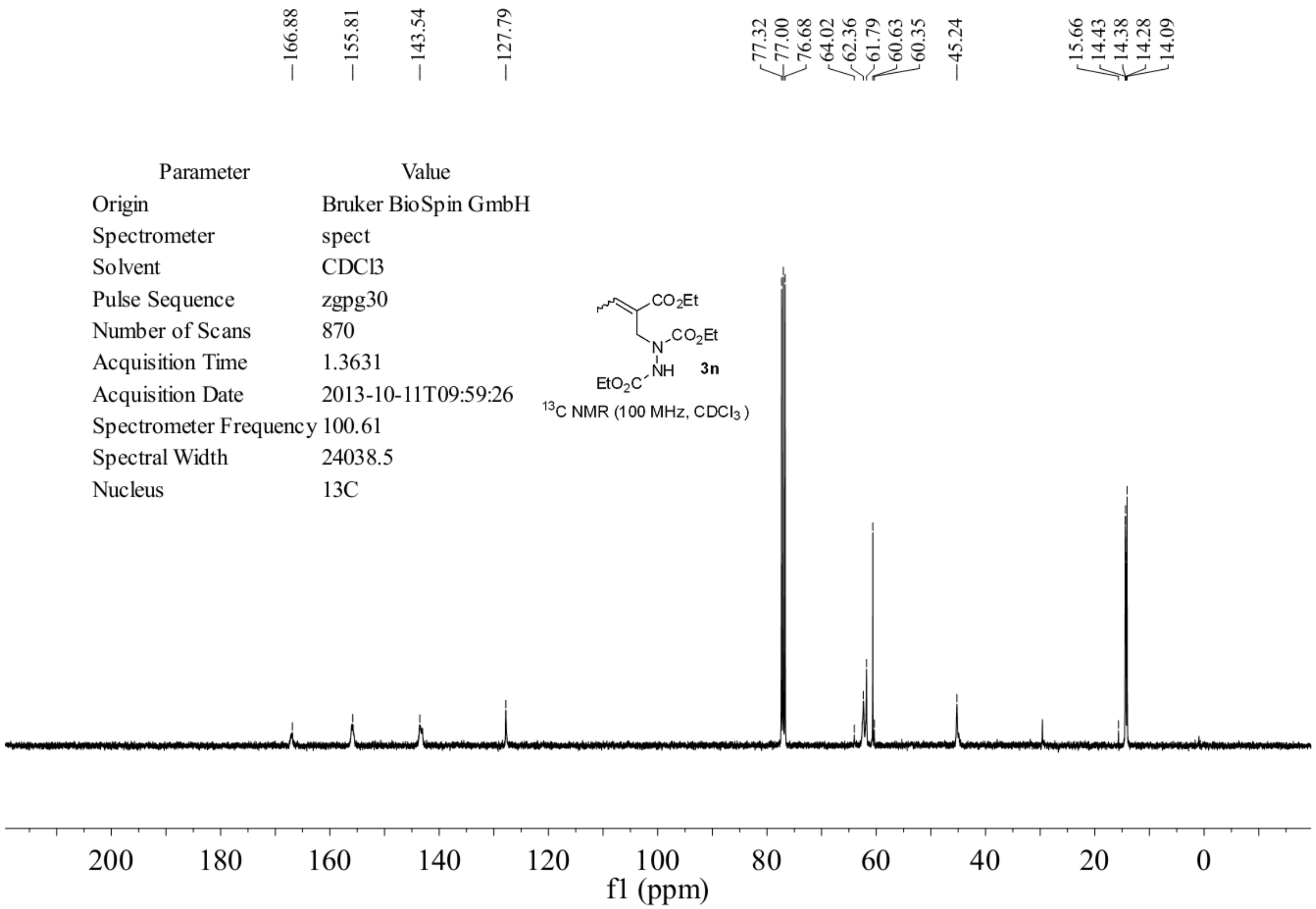

166.88
155.81
143.54
127.79
77.32
77.00
76.68
64.02
62.36
61.79
60.63
60.35
45.24
15.66
14.43
14.38
14.28
14.09
Parameter Value
Origin Bruker BioSpin GmbH
Spectrometer spect
Solvent CDCl3
Pulse Sequence zgpg30
Number of Scans 870
Acquisition Time 1.3631
Acquisition Date 2013-10-11T09:59:26
Spectrometer Frequency 100.61
Spectral Width 24038.5
Nucleus 13C
CO2Et
CO2Et
N
NH
EtO2C
3n
13C NMR (100 MHz, CDCl3)
200 180 160 140 120 100 80 60 40 20 0
f1 (ppm)

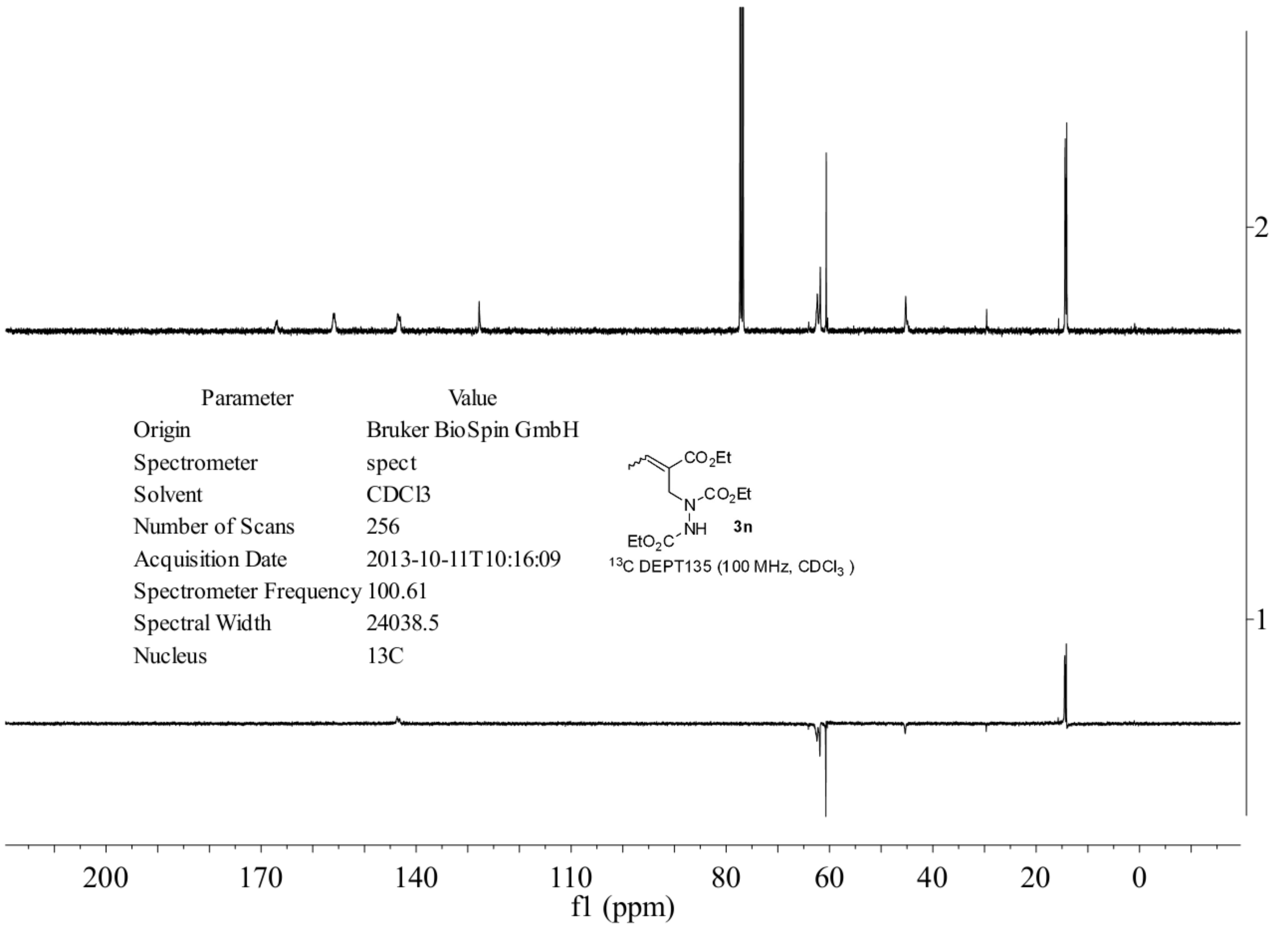

Parameter Value
Origin Bruker BioSpin GmbH
Spectrometer spect
Solvent CDCl3
Number of Scans 256
Acquisition Date 2013-10-11T10:16:09
Spectrometer Frequency 100.61
Spectral Width 24038.5
Nucleus 13C
CO2Et
N CO2Et
EtO2C NH
3n
13C DEPT135 (100 MHz, CDCl3)
2
1
200 170 140 110 80 60 40 20 0
f1 (ppm)

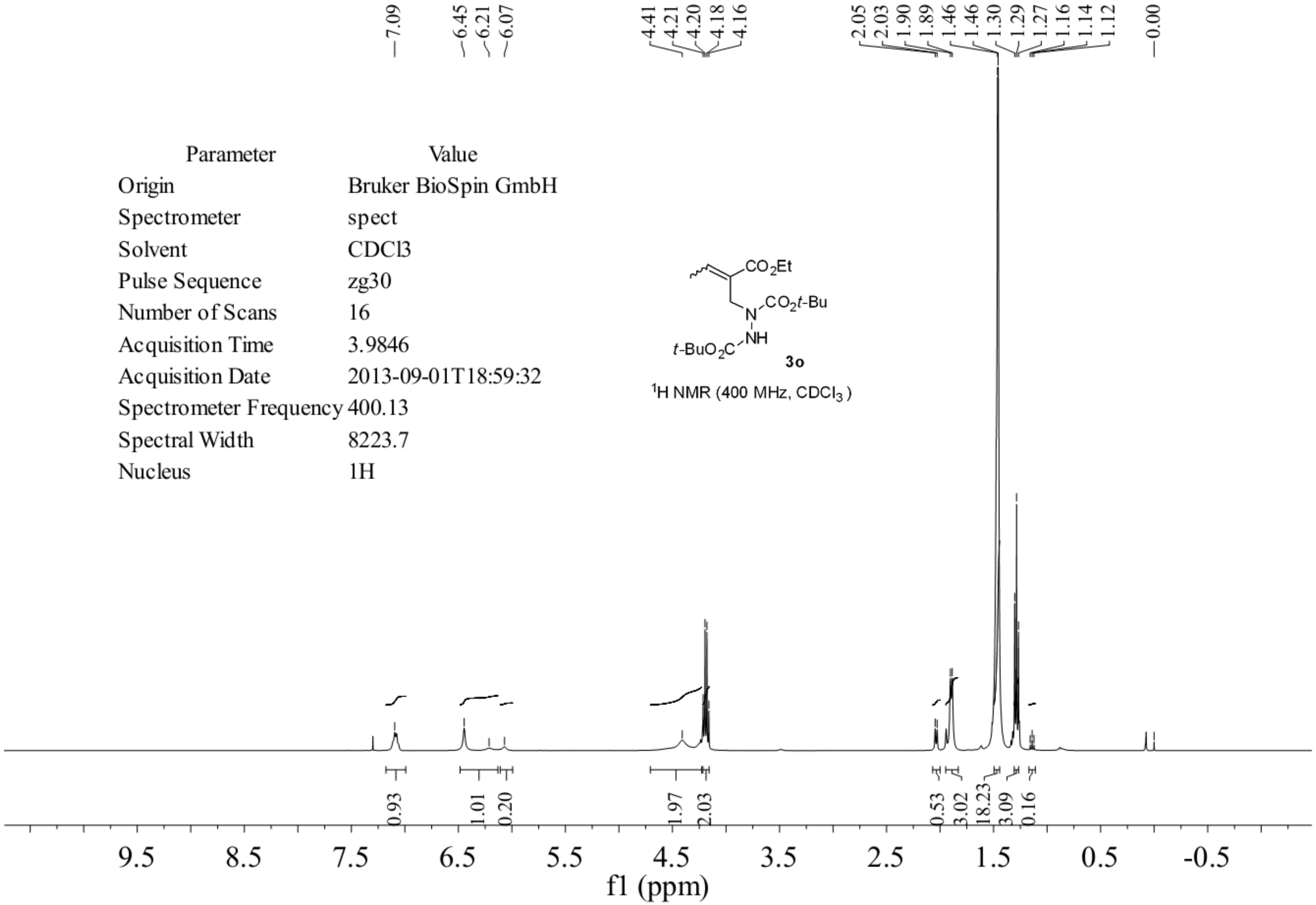
7.09
6.45
6.21
6.07
4.41
4.21
4.20
4.18
4.16
2.05
2.03
1.90
1.89
1.46
1.46
1.30
1.29
1.27
1.16
1.14
1.12
0.00
Parameter Value
Origin Bruker BioSpin GmbH
Spectrometer spect
Solvent CDCl3
Pulse Sequence zg30
Number of Scans 16
Acquisition Time 3.9846
Acquisition Date 2013-09-01T18:59:32
Spectrometer Frequency 400.13
Spectral Width 8223.7
Nucleus 1H
CO2Et
N
CO2t-Bu
NH
t-BuO2C
3o
1H NMR (400 MHz, CDCl3)
0.93
1.01
0.20
1.97
2.03
0.53
3.02
18.23
3.09
0.16
9.5
8.5
7.5
6.5
5.5
4.5
3.5
2.5
1.5
0.5
-0.5
f1 (ppm)

Parameter	Value
Origin	Bruker BioSpin GmbH
Spectrometer	spect
Solvent	CDCl3
Pulse Sequence	zgpg30
Number of Scans	590
Acquisition Time	1.3631
Acquisition Date	2013-09-01T19:36:21
Spectrometer Frequency	100.61
Spectral Width	24038.5
Nucleus	13C

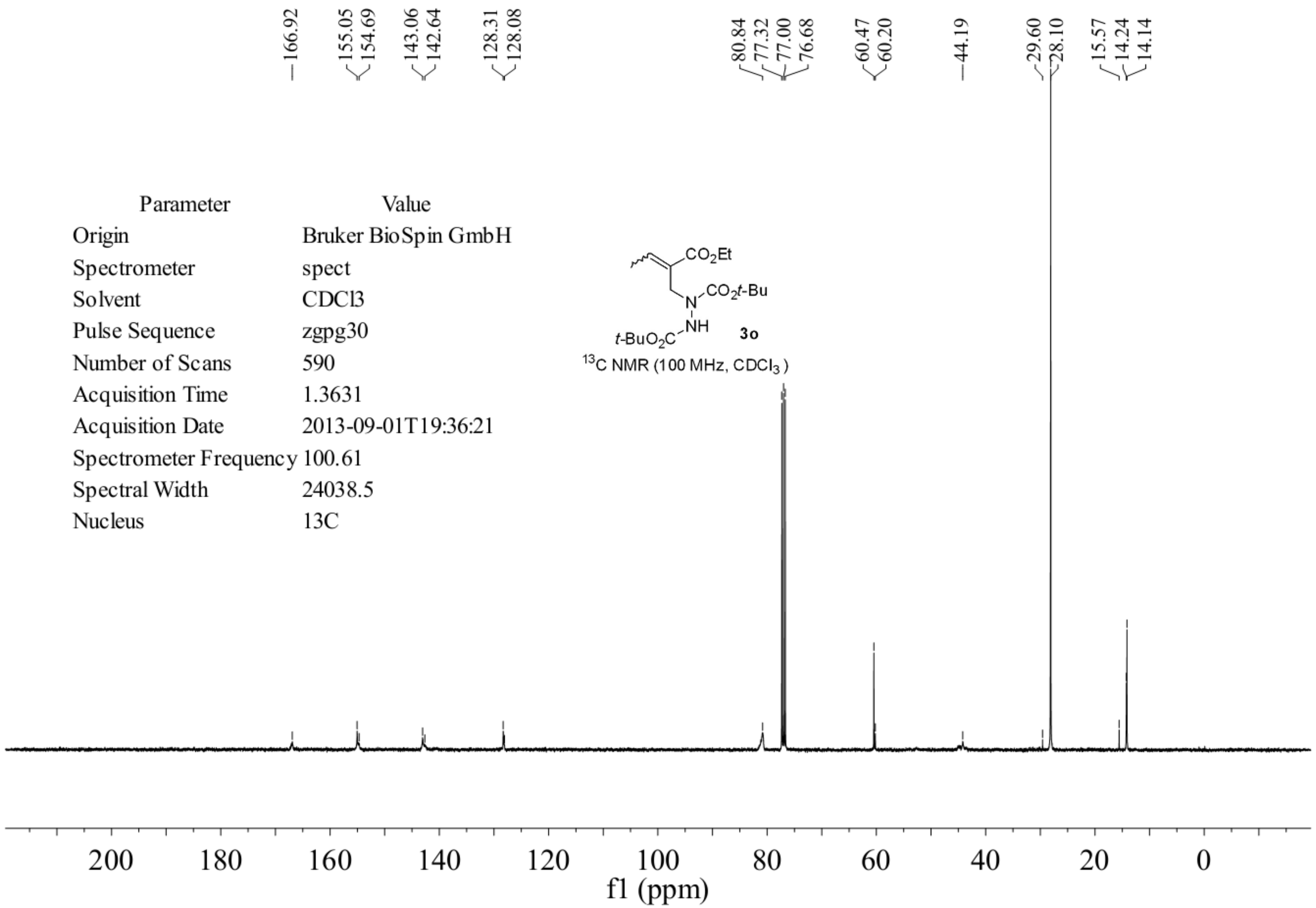

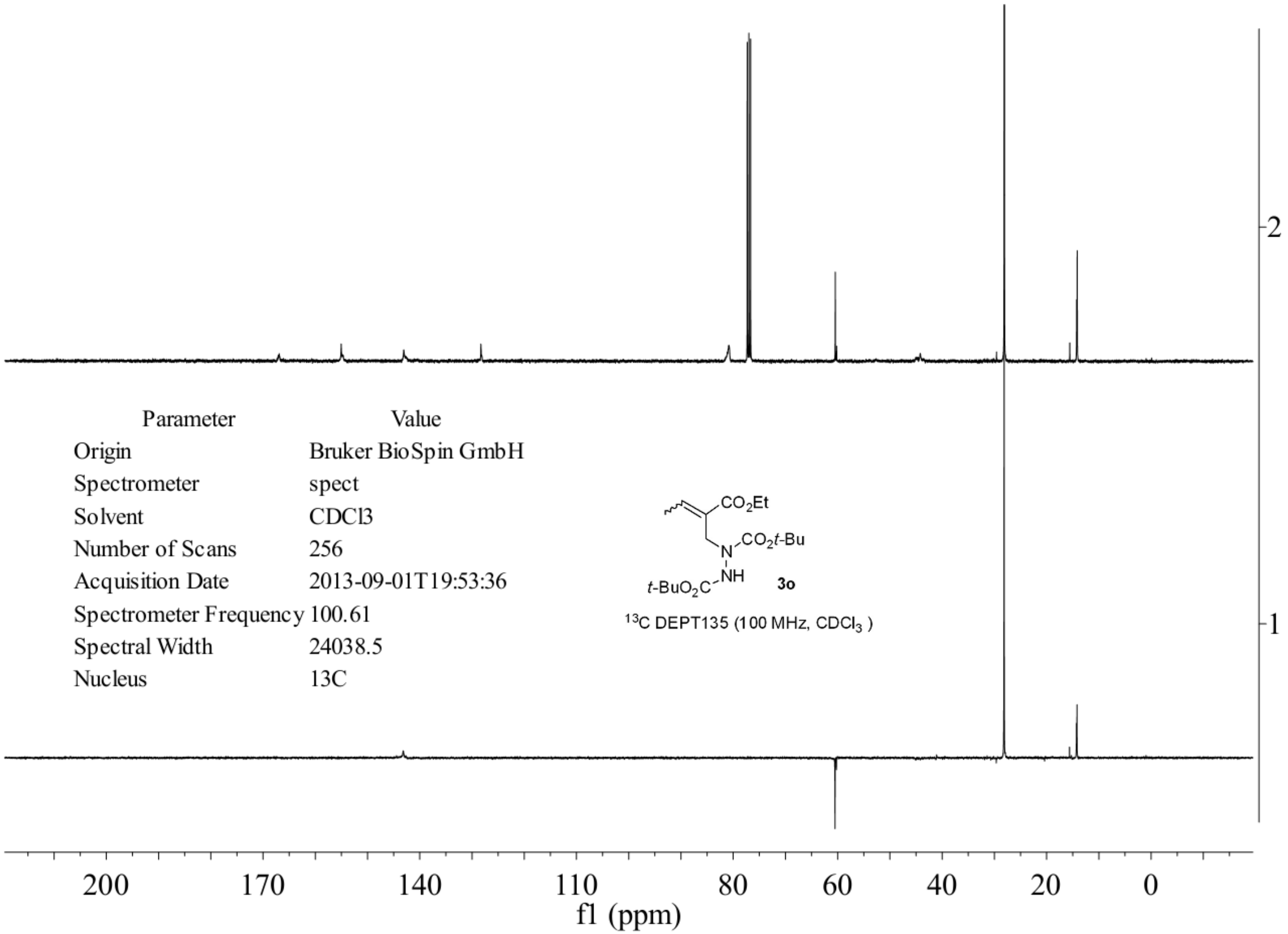

Parameter Value
Origin Bruker BioSpin GmbH
Spectrometer spect
Solvent CDCl3
Number of Scans 256
Acquisition Date 2013-09-01T19:53:36
Spectrometer Frequency 100.61
Spectral Width 24038.5
Nucleus 13C
CO2Et
CO2t-Bu
N
NH
t-BuO2C
3o
13C DEPT135 (100 MHz, CDCl3)
2
1
200
170
140
110
80
60
40
20
0
f1 (ppm)

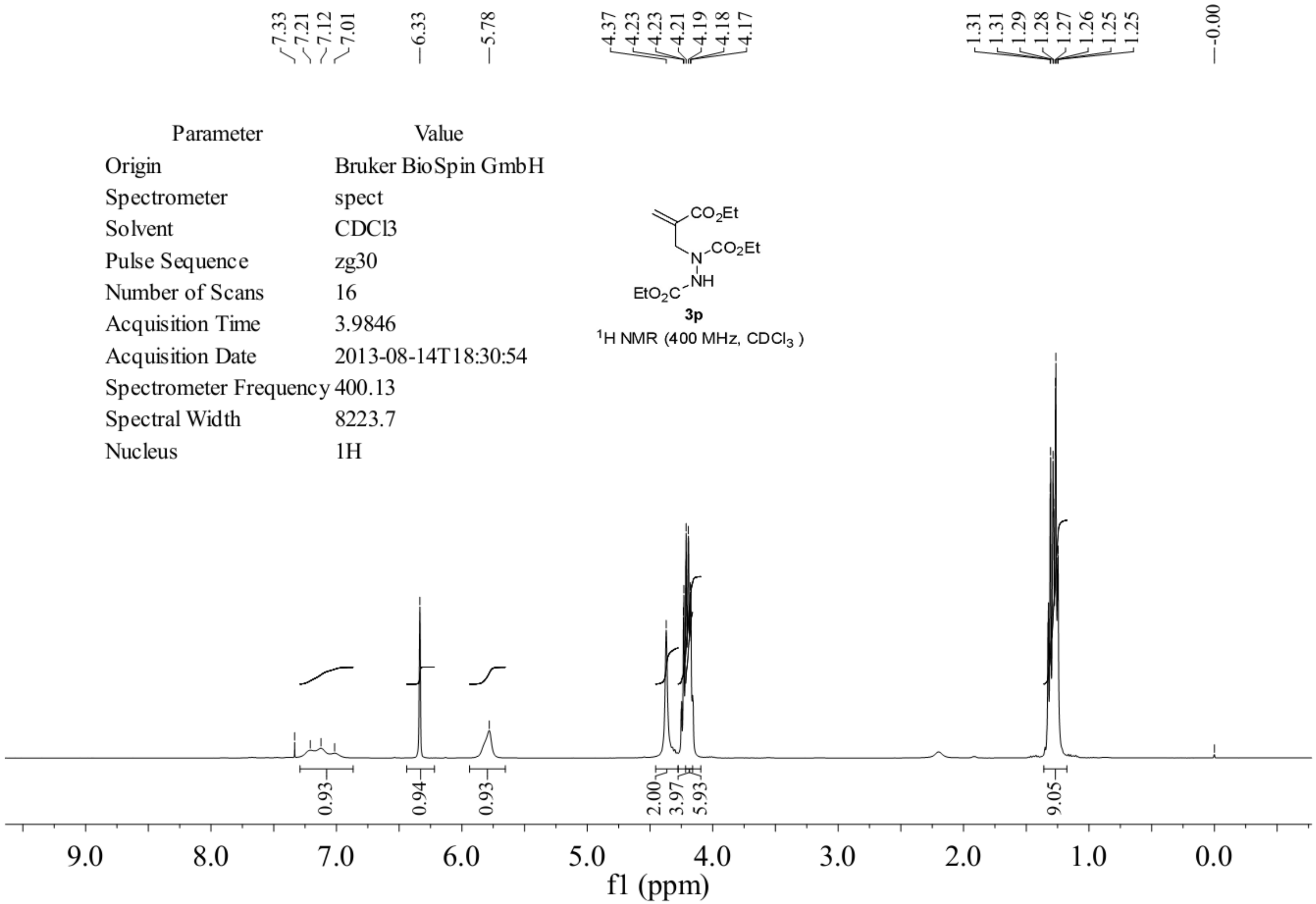
7.33
7.21
7.12
7.01
6.33
5.78
4.37
4.23
4.23
4.21
4.19
4.18
4.17
1.31
1.31
1.29
1.28
1.27
1.26
1.25
1.25
-0.00
Parameter Value
Origin Bruker BioSpin GmbH
Spectrometer spect
Solvent CDCl3
Pulse Sequence zg30
Number of Scans 16
Acquisition Time 3.9846
Acquisition Date 2013-08-14T18:30:54
Spectrometer Frequency 400.13
Spectral Width 8223.7
Nucleus 1H
CO2Et
N
CO2Et
NH
EtO2C
3p
1H NMR (400 MHz, CDCl3)
0.93
0.94
0.93
2.00
3.97
5.93
9.05
9.0
8.0
7.0
6.0
5.0
4.0
3.0
2.0
1.0
0.0
f1 (ppm)

Parameter	Value
Origin	Bruker BioSpin GmbH
Spectrometer	spect
Solvent	CDCl3
Number of Scans	370
Acquisition Date	2013-08-14T18:55:20
Spectrometer Frequency	100.61
Spectral Width	24038.5
Nucleus	13C

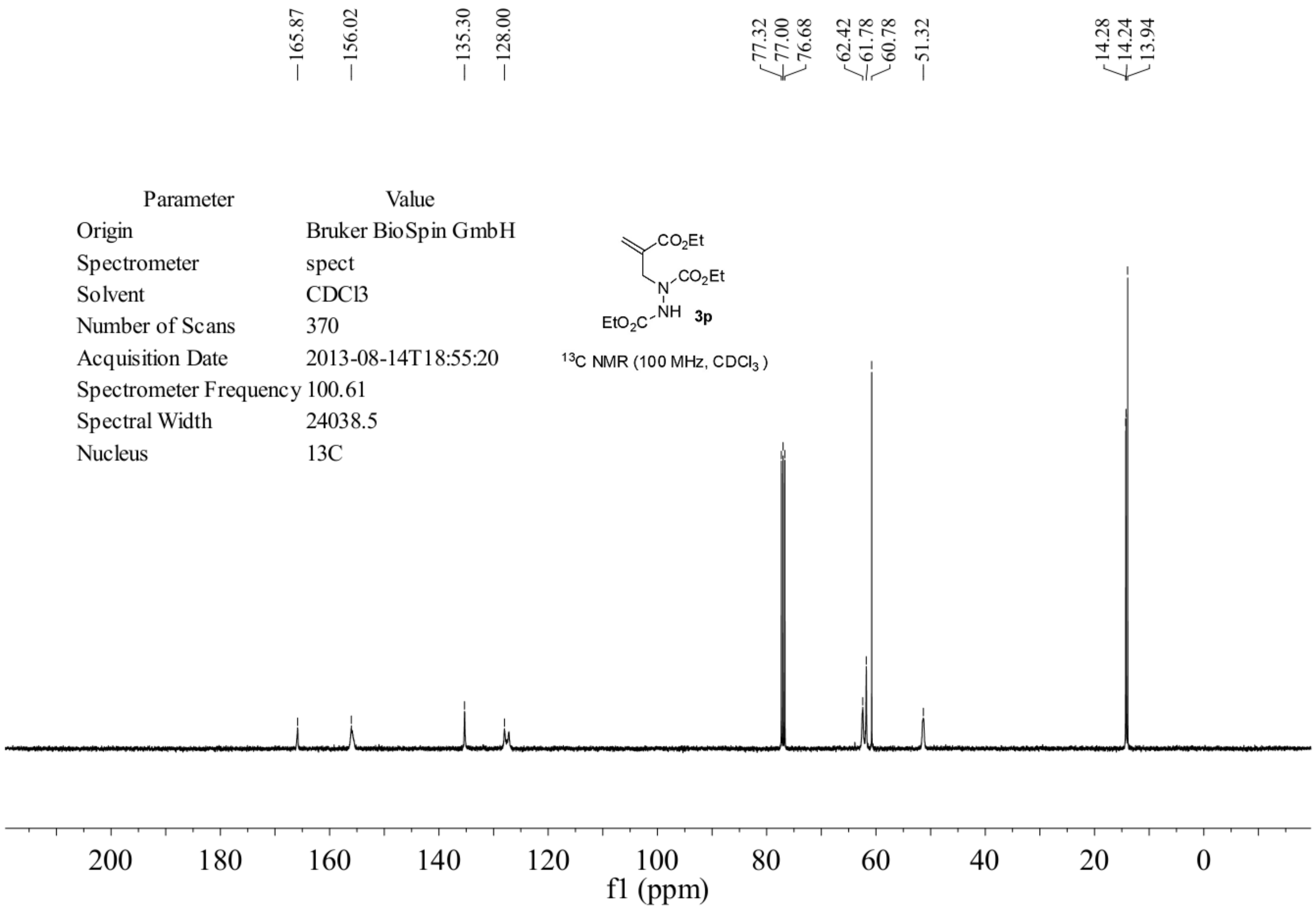

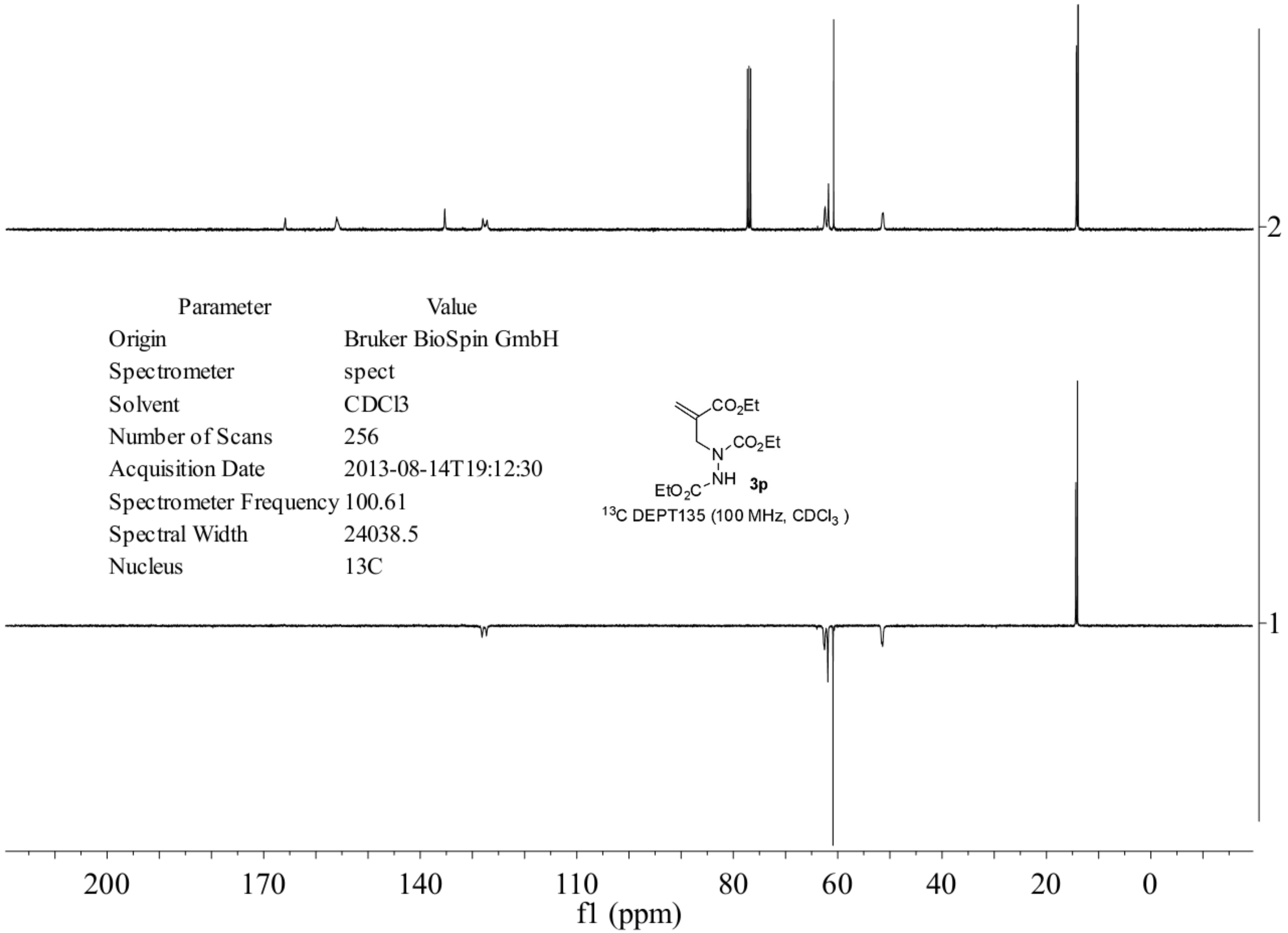
Parameter
Value
Origin
Bruker BioSpin GmbH
Spectrometer
spect
Solvent
CDCl3
Number of Scans
256
Acquisition Date
2013-08-14T19:12:30
Spectrometer Frequency
100.61
Spectral Width
24038.5
Nucleus
13C
CO2Et
CO2Et
N
NH
EtO2C
3p
13C DEPT135 (100 MHz, CDCl3)
2
1
200
170
140
110
80
60
40
20
0
f1 (ppm)

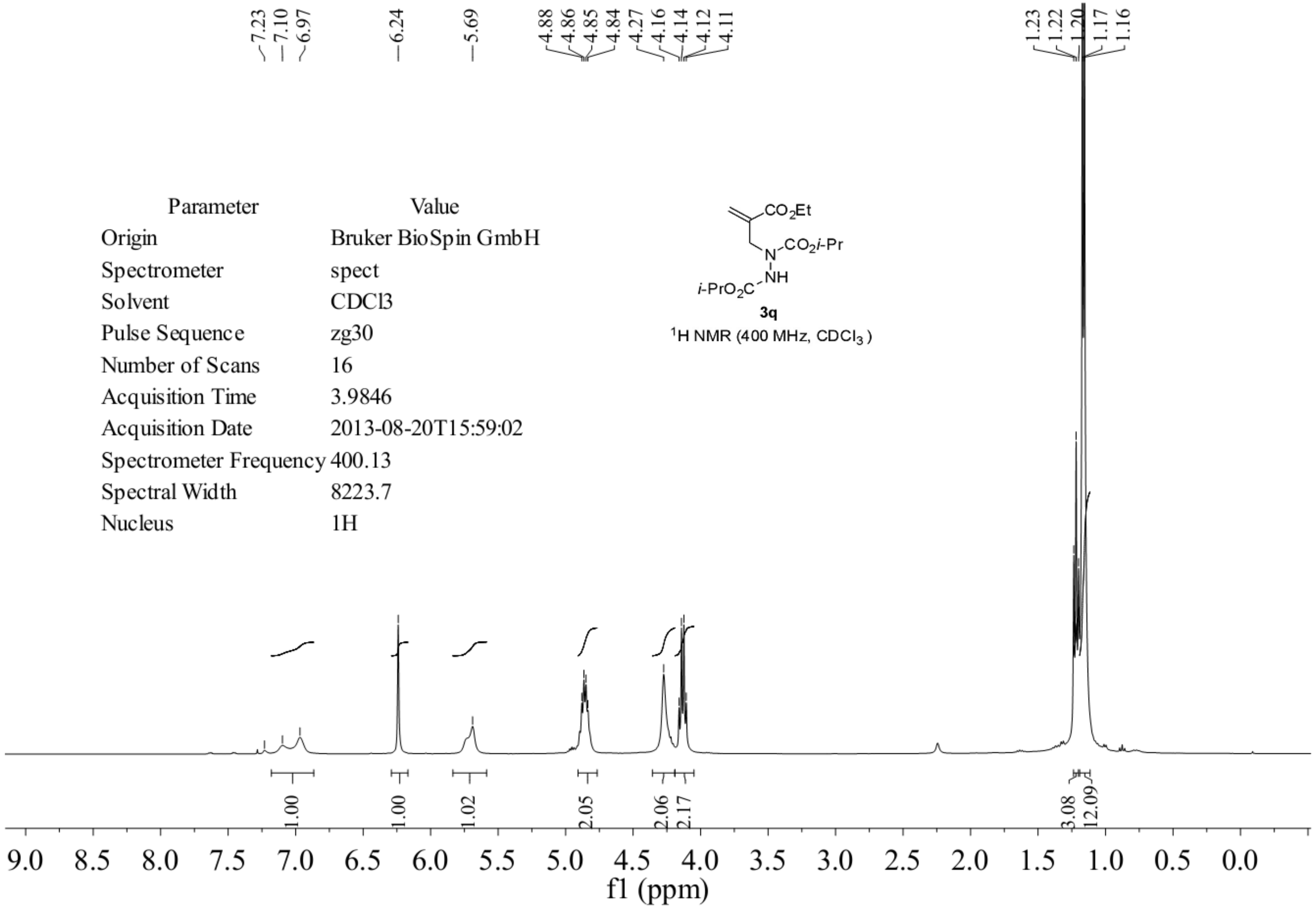
7.23
7.10
6.97
6.24
5.69
4.88
4.86
4.85
4.84
4.27
4.16
4.14
4.12
4.11
1.23
1.22
1.20
1.17
1.16
Parameter Value
Origin Bruker BioSpin GmbH
Spectrometer spect
Solvent CDCl3
Pulse Sequence zg30
Number of Scans 16
Acquisition Time 3.9846
Acquisition Date 2013-08-20T15:59:02
Spectrometer Frequency 400.13
Spectral Width 8223.7
Nucleus 1H
CO2Et
N
CO2i-Pr
NH
i-PrO2C
3q
1H NMR (400 MHz, CDCl3)
1.00
1.00
1.02
2.05
2.06
2.17
3.08
12.09
9.0 8.5 8.0 7.5 7.0 6.5 6.0 5.5 5.0 4.5 4.0 3.5 3.0 2.5 2.0 1.5 1.0 0.5 0.0
f1 (ppm)

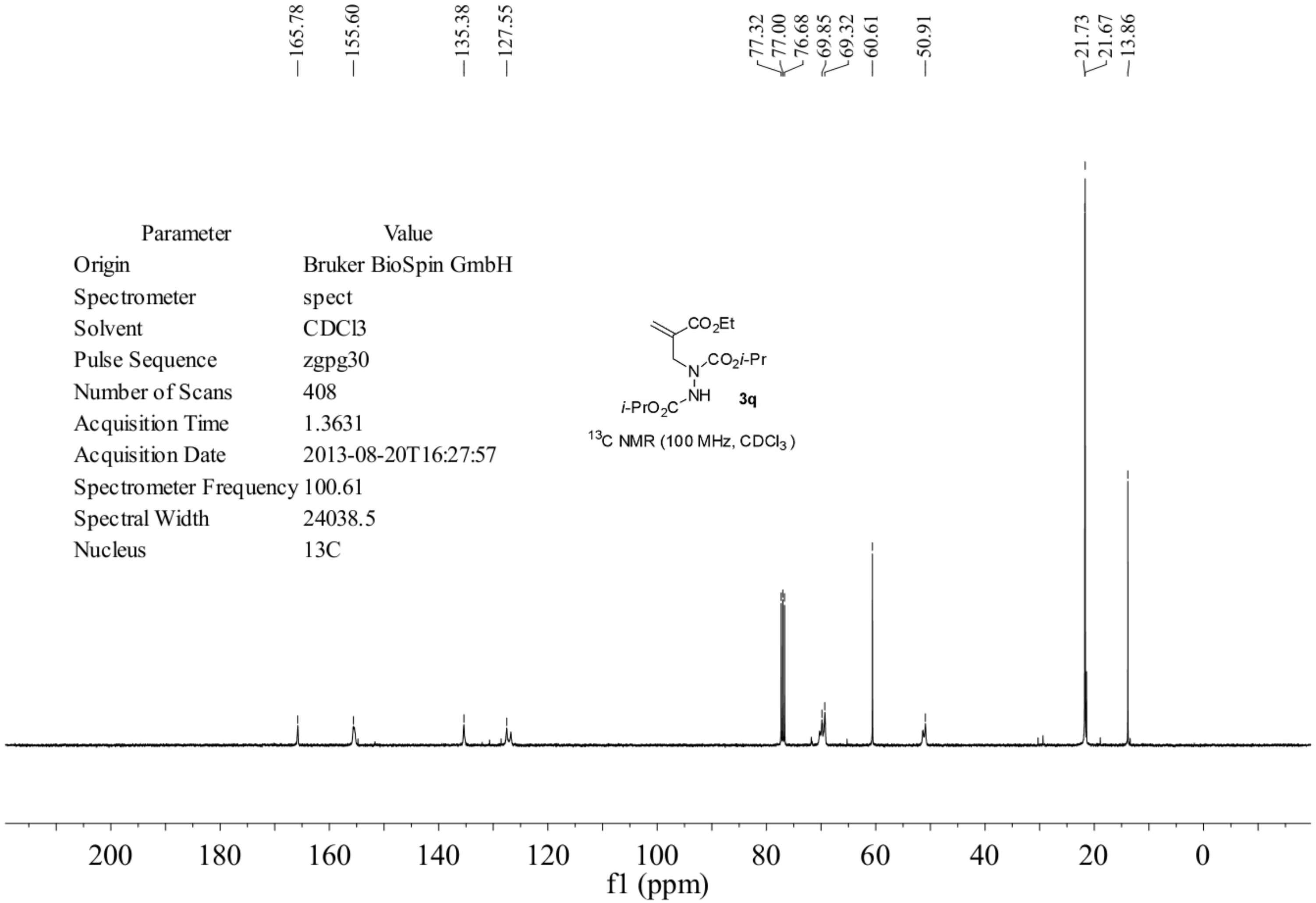
165.78
155.60
135.38
127.55
77.32
77.00
76.68
69.85
69.32
60.61
50.91
21.73
21.67
13.86
Parameter Value
Origin Bruker BioSpin GmbH
Spectrometer spect
Solvent CDCl3
Pulse Sequence zgpg30
Number of Scans 408
Acquisition Time 1.3631
Acquisition Date 2013-08-20T16:27:57
Spectrometer Frequency 100.61
Spectral Width 24038.5
Nucleus 13C
CO2Et
CO2i-Pr
N
NH
i-PrO2C
3q
13C NMR (100 MHz, CDCl3)
200
180
160
140
120
100
80
60
40
20
0
f1 (ppm)

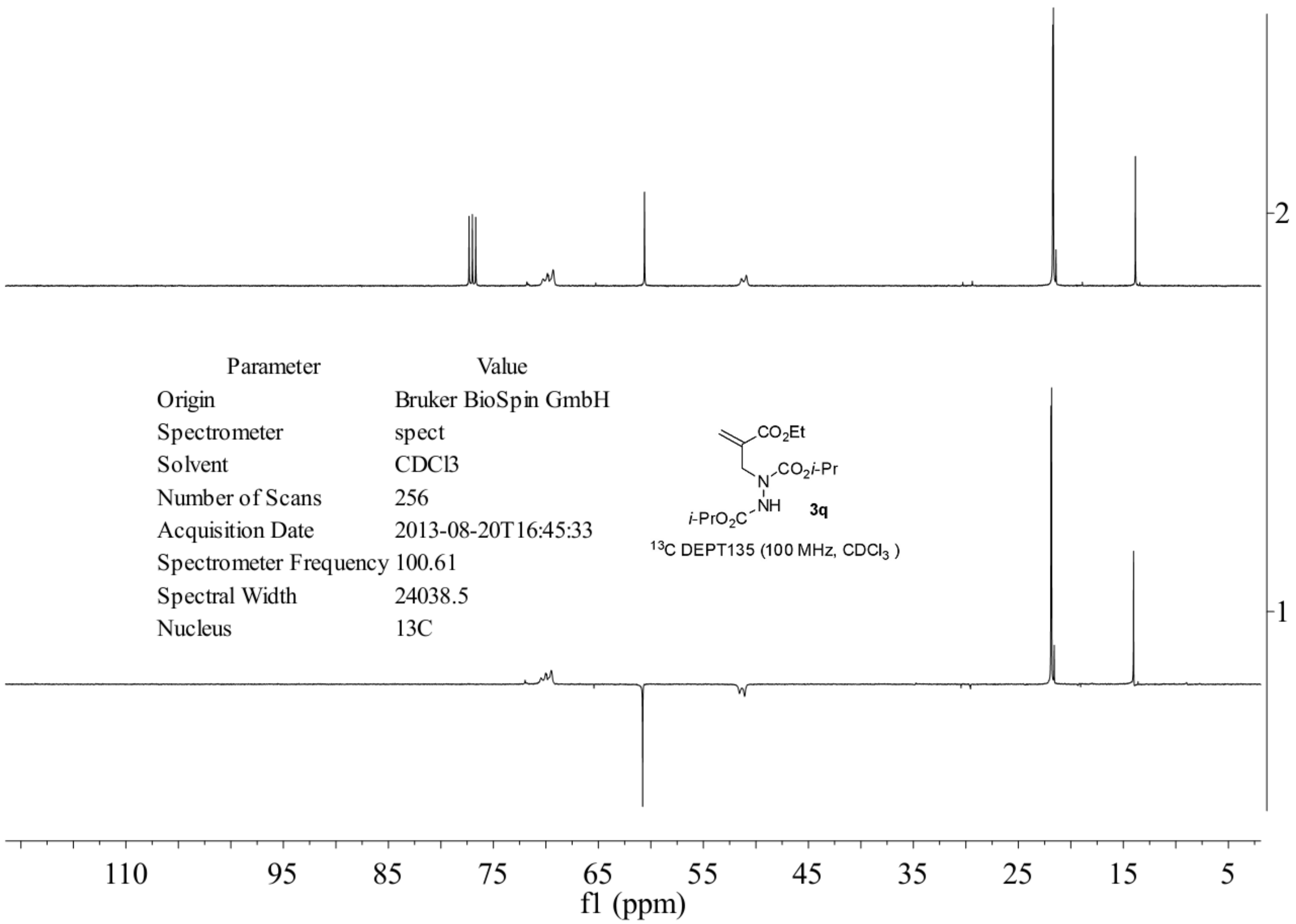

Parameter
Value
Origin
Bruker BioSpin GmbH
Spectrometer
spect
Solvent
CDCl3
Number of Scans
256
Acquisition Date
2013-08-20T16:45:33
Spectrometer Frequency
100.61
Spectral Width
24038.5
Nucleus
13C
CO2Et
CO2i-Pr
N
NH
i-PrO2C
3q
13C DEPT135 (100 MHz, CDCl3)
2
1
110
95
85
75
65
55
45
35
25
15
5
f1 (ppm)

Parameter	Value
Origin	Bruker BioSpin GmbH
Spectrometer	spect
Solvent	CDCl3
Pulse Sequence	zg30
Number of Scans	16
Acquisition Time	3.9846
Acquisition Date	2013-09-01T17:33:27
Spectrometer Frequency	400.13
Spectral Width	8223.7
Nucleus	1H

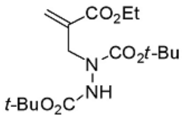

3r

^1H NMR (400 MHz, $CDCl_3$)

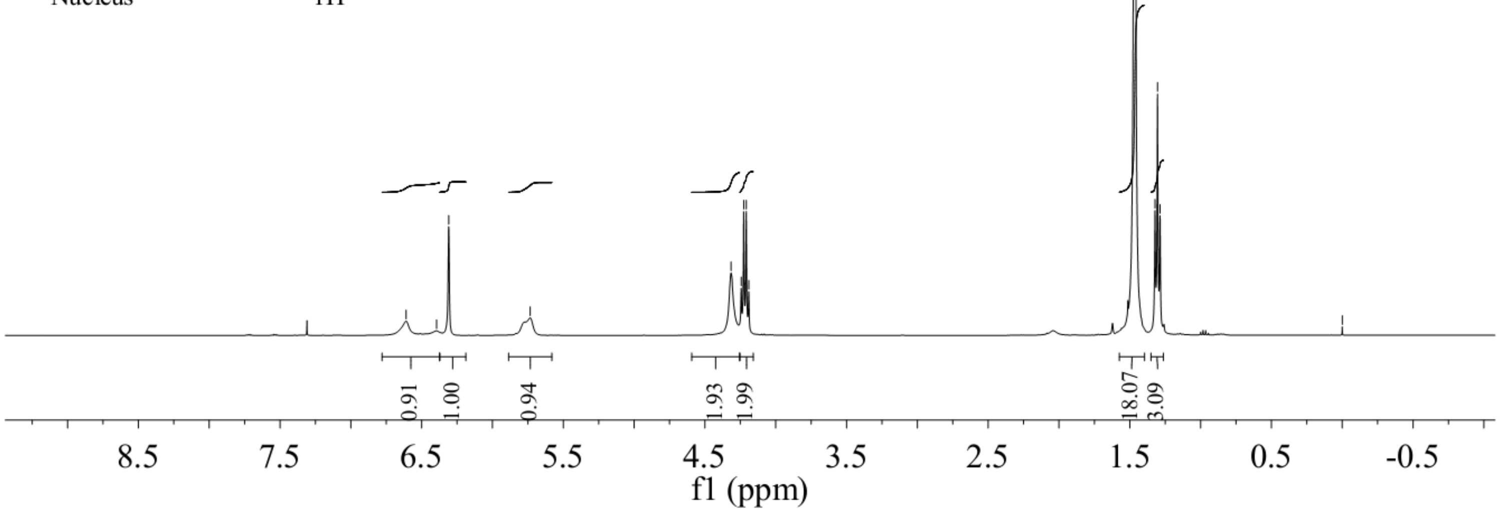

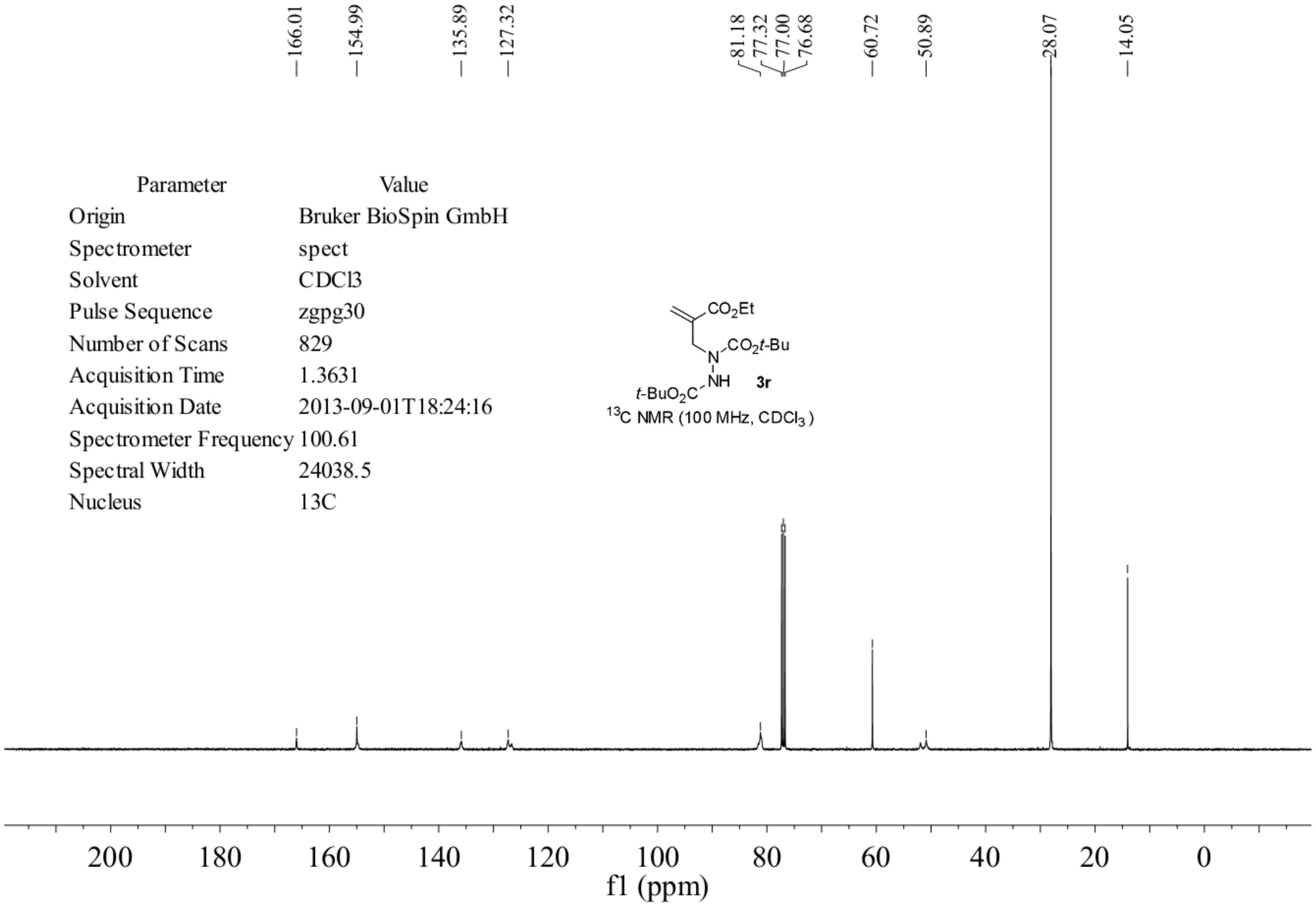
166.01
154.99
135.89
127.32
81.18
77.32
77.00
76.68
60.72
50.89
28.07
14.05
Parameter Value
Origin Bruker BioSpin GmbH
Spectrometer spect
Solvent CDCl3
Pulse Sequence zgpg30
Number of Scans 829
Acquisition Time 1.3631
Acquisition Date 2013-09-01T18:24:16
Spectrometer Frequency 100.61
Spectral Width 24038.5
Nucleus 13C
CO2Et
CO2t-Bu
N
NH
t-BuO2C
3r
13C NMR (100 MHz, CDCl3)
200 180 160 140 120 100 80 60 40 20 0
f1 (ppm)

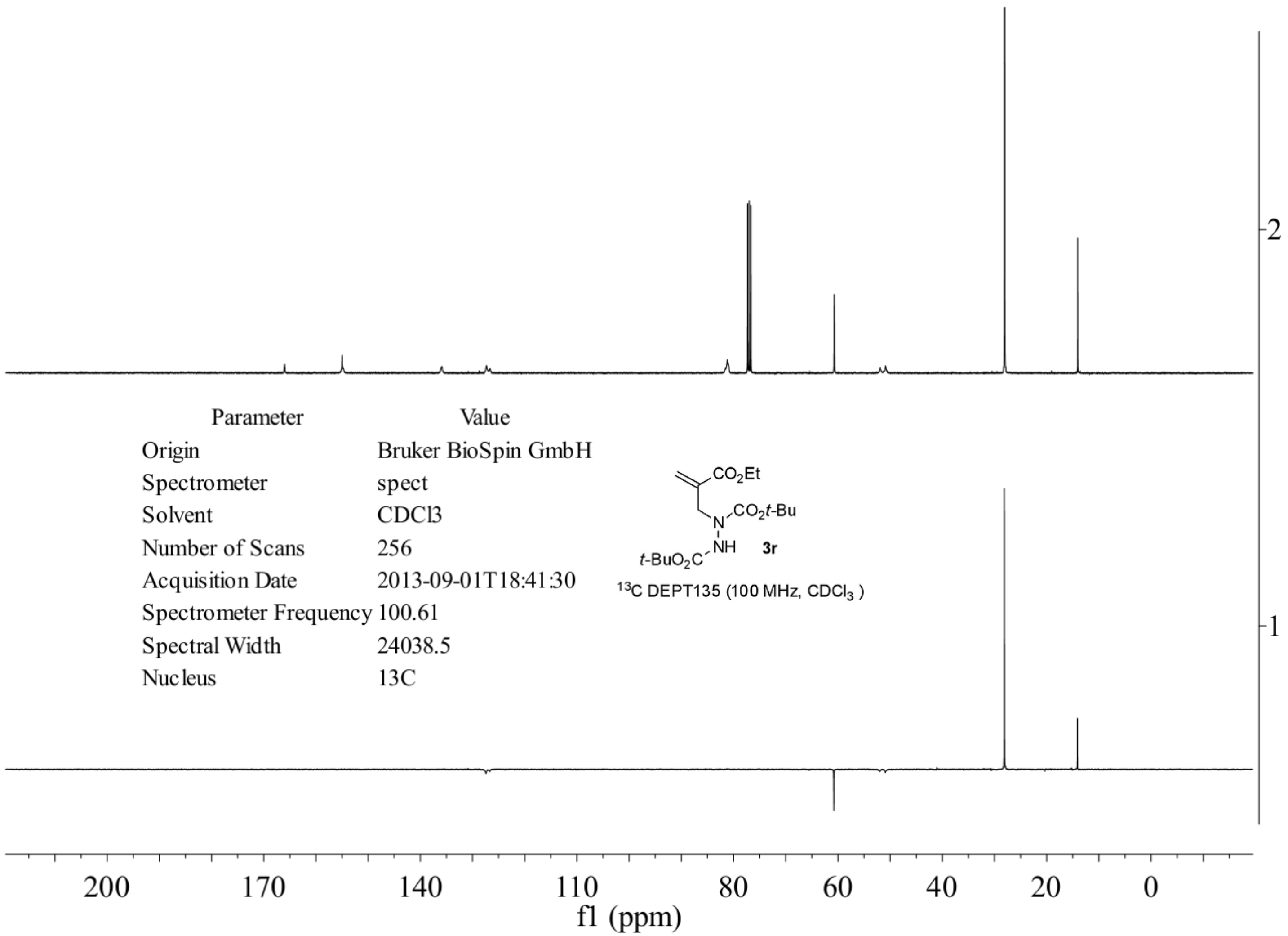
Parameter Value
Origin Bruker BioSpin GmbH
Spectrometer spect
Solvent CDCl3
Number of Scans 256
Acquisition Date 2013-09-01T18:41:30
Spectrometer Frequency 100.61
Spectral Width 24038.5
Nucleus 13C
CO2Et
CO2t-Bu
N
NH
t-BuO2C
3r
13C DEPT135 (100 MHz, CDCl3)
2
1
200 170 140 110 80 60 40 20 0
f1 (ppm)

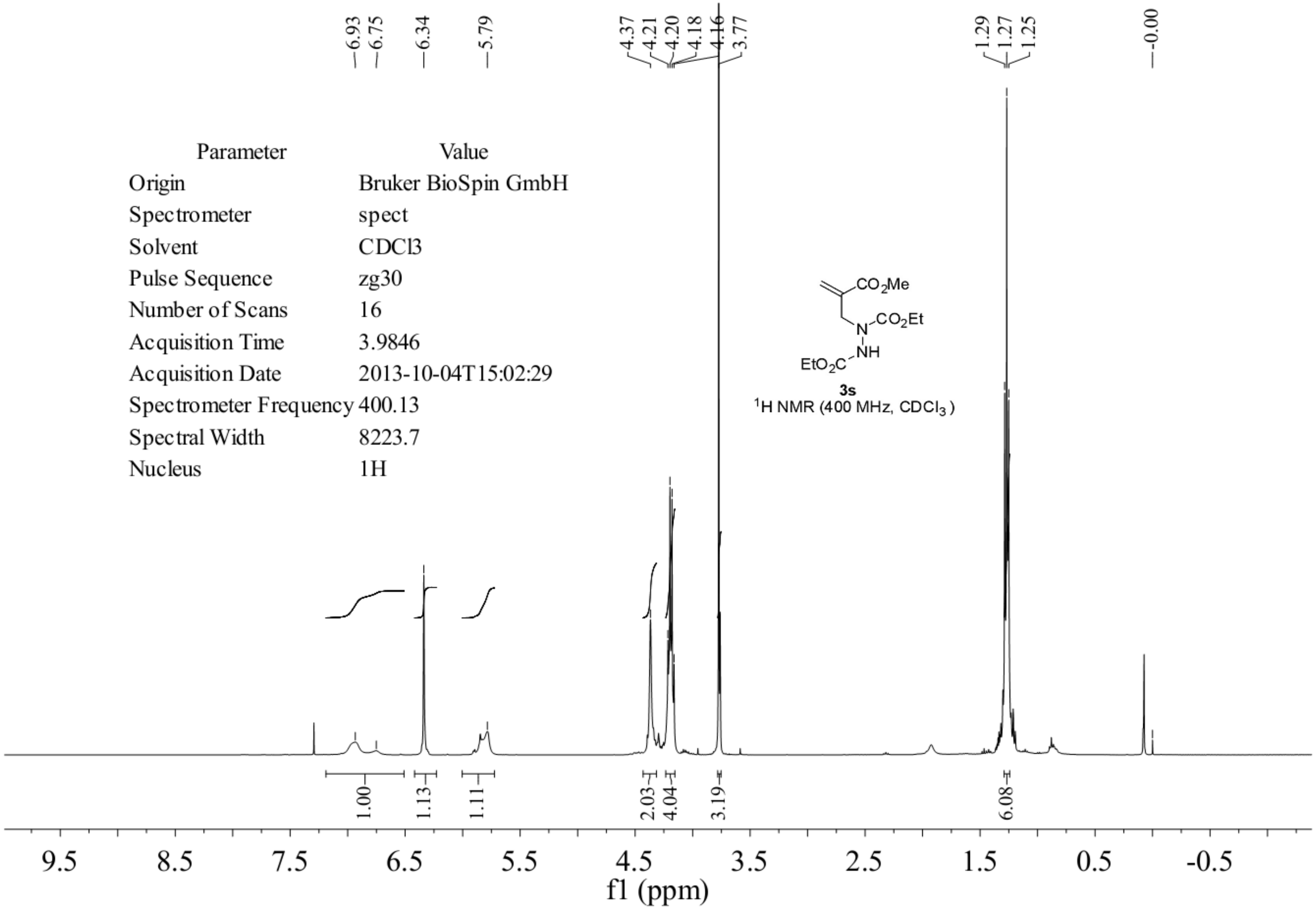
6.93
6.75
6.34
5.79
4.37
4.21
4.20
4.18
4.16
3.77
1.29
1.27
1.25
0.00
Parameter Value
Origin Bruker BioSpin GmbH
Spectrometer spect
Solvent CDCl3
Pulse Sequence zg30
Number of Scans 16
Acquisition Time 3.9846
Acquisition Date 2013-10-04T15:02:29
Spectrometer Frequency 400.13
Spectral Width 8223.7
Nucleus 1H
CO2Me
CO2Et
N
NH
EtO2C
3s
1H NMR (400 MHz, CDCl3)
1.00
1.13
1.11
2.03
4.04
3.19
6.08
9.5
8.5
7.5
6.5
5.5
4.5
3.5
2.5
1.5
0.5
-0.5
f1 (ppm)

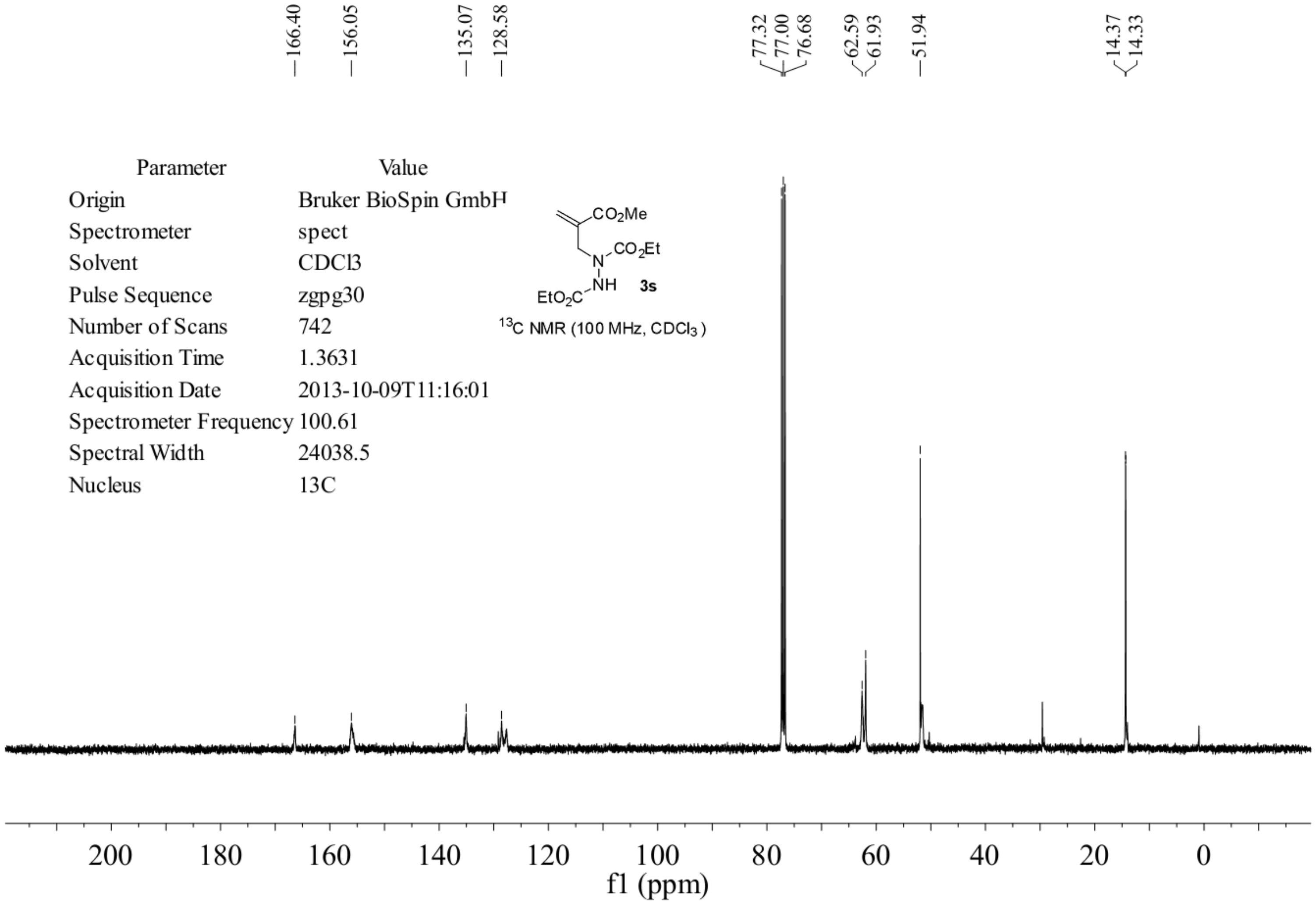
166.40
156.05
135.07
128.58
77.32
77.00
76.68
62.59
61.93
51.94
14.37
14.33
Parameter Value
Origin Bruker BioSpin GmbH
Spectrometer spect
Solvent CDCl3
Pulse Sequence zgpg30
Number of Scans 742
Acquisition Time 1.3631
Acquisition Date 2013-10-09T11:16:01
Spectrometer Frequency 100.61
Spectral Width 24038.5
Nucleus 13C
CO2Me
CO2Et
N
NH
EtO2C
3s
13C NMR (100 MHz, CDCl3)
200 180 160 140 120 100 80 60 40 20 0
f1 (ppm)

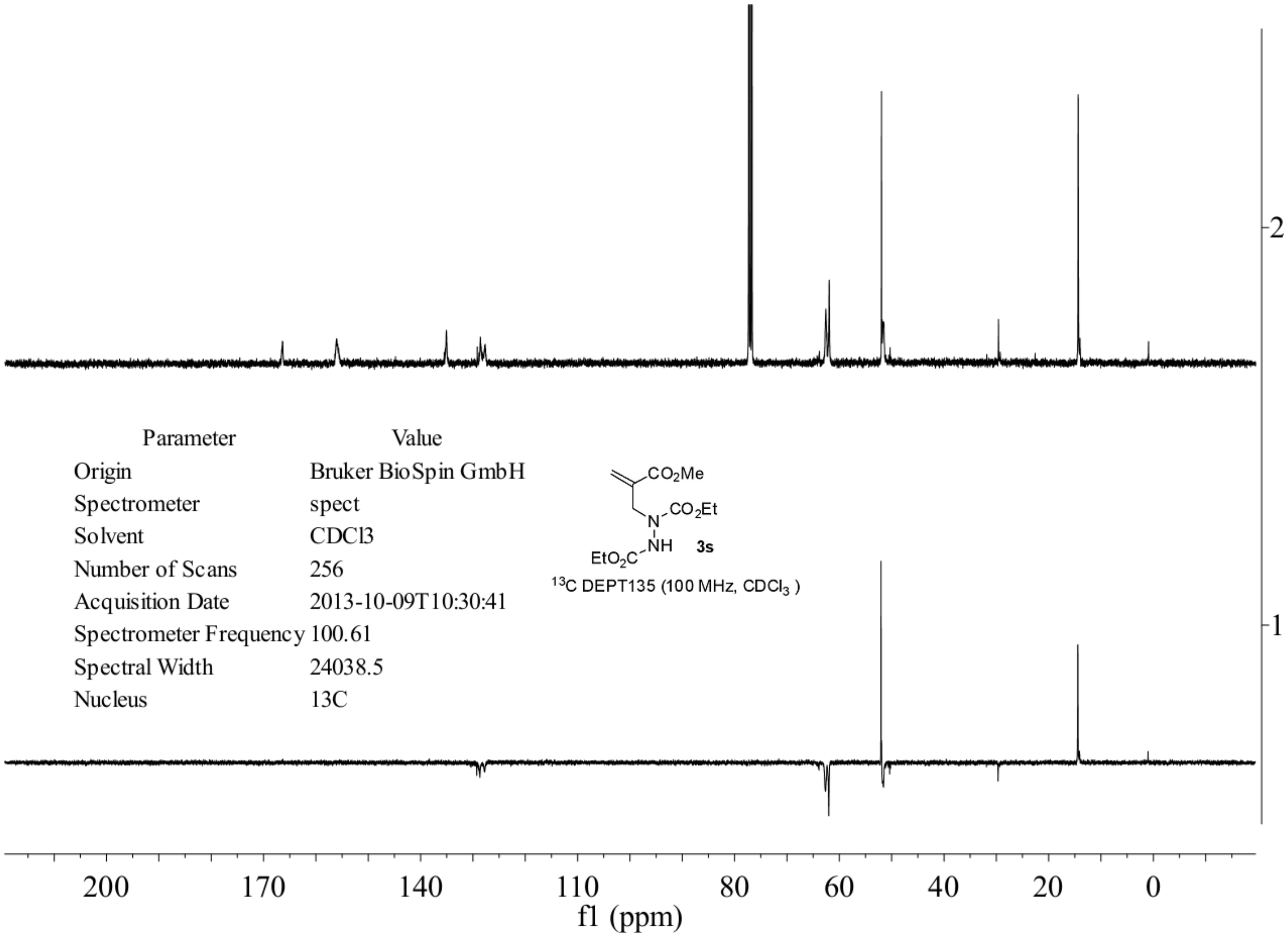

Parameter Value
Origin Bruker BioSpin GmbH
Spectrometer spect
Solvent CDCl3
Number of Scans 256
Acquisition Date 2013-10-09T10:30:41
Spectrometer Frequency 100.61
Spectral Width 24038.5
Nucleus 13C
CO2Me
CO2Et
N
NH
EtO2C
3s
13C DEPT135 (100 MHz, CDCl3)
2
1
200
170
140
110
80
60
40
20
0
f1 (ppm)

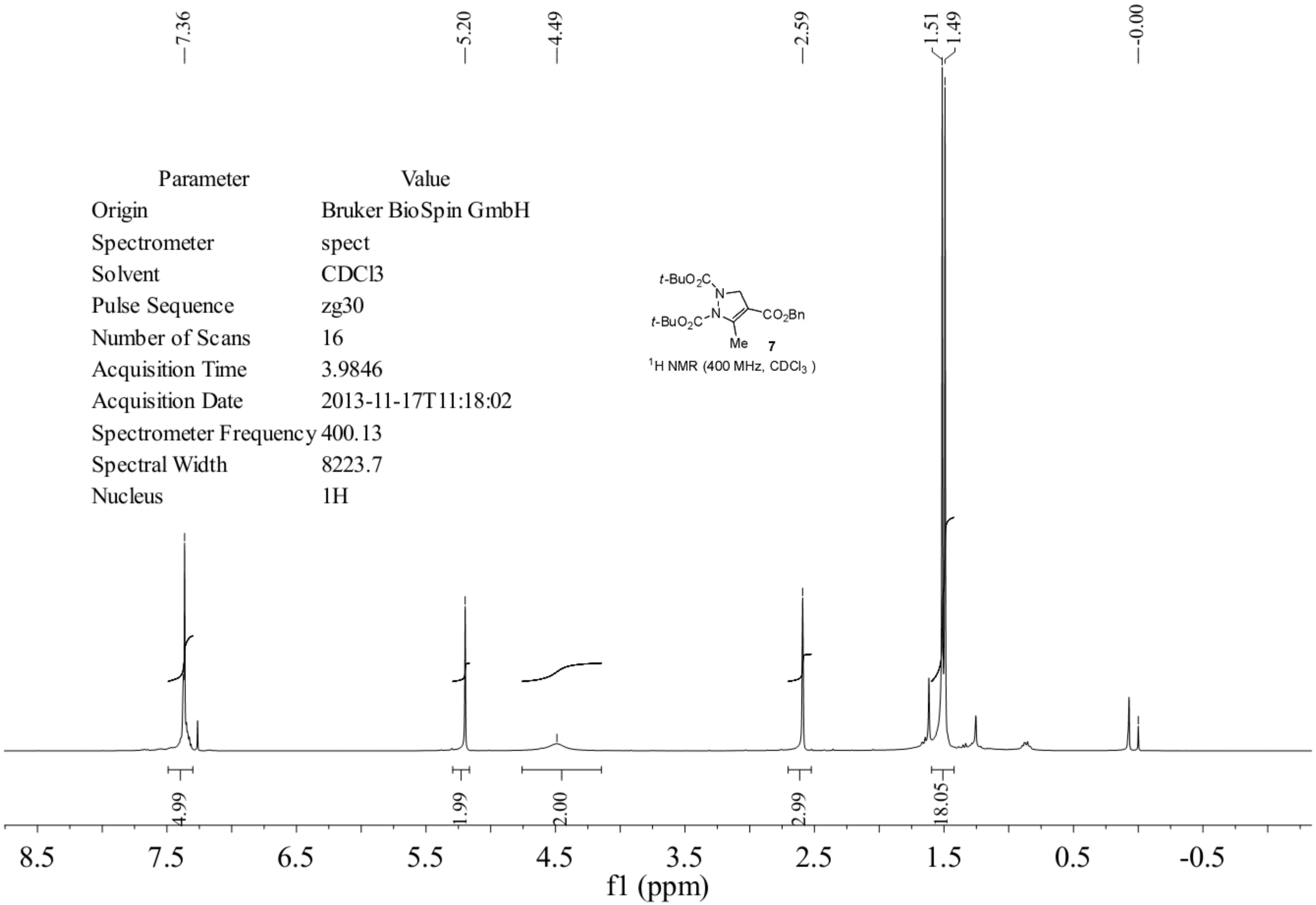
7.36
5.20
4.49
2.59
1.51
1.49
-0.00
Parameter Value
Origin Bruker BioSpin GmbH
Spectrometer spect
Solvent CDCl3
Pulse Sequence zg30
Number of Scans 16
Acquisition Time 3.9846
Acquisition Date 2013-11-17T11:18:02
Spectrometer Frequency 400.13
Spectral Width 8223.7
Nucleus 1H
t-BuO2C
N
t-BuO2C
N
CO2Bn
Me
7
1H NMR (400 MHz, CDCl3)
4.99
1.99
2.00
2.99
18.05
8.5
7.5
6.5
5.5
4.5
3.5
2.5
1.5
0.5
-0.5
f1 (ppm)

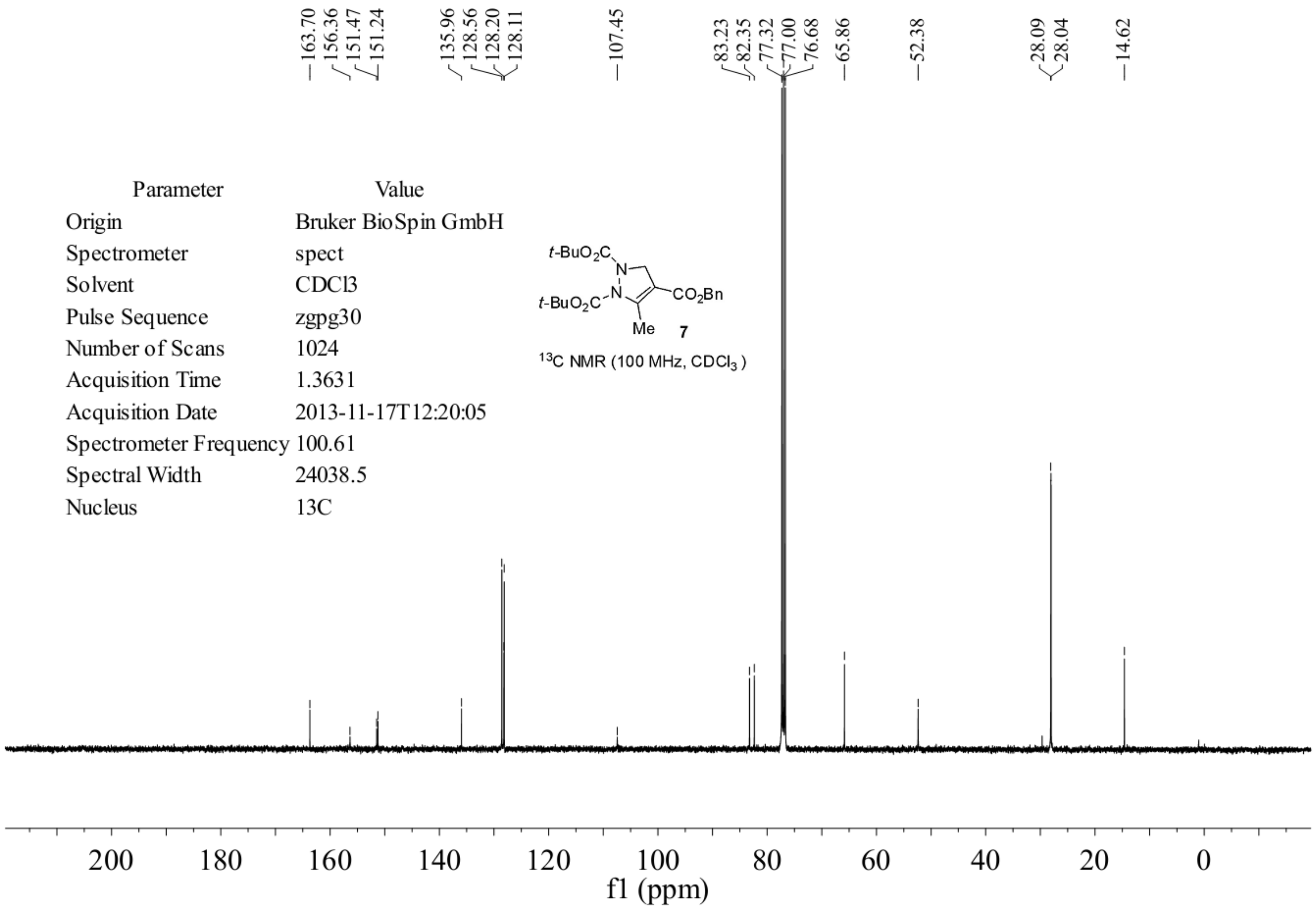

163.70
156.36
151.47
151.24
135.96
128.56
128.20
128.11
107.45
83.23
82.35
77.32
77.00
76.68
65.86
52.38
28.09
28.04
14.62
Parameter
Value
Origin
Bruker BioSpin GmbH
Spectrometer
spect
Solvent
CDCl3
Pulse Sequence
zgpg30
Number of Scans
1024
Acquisition Time
1.3631
Acquisition Date
2013-11-17T12:20:05
Spectrometer Frequency
100.61
Spectral Width
24038.5
Nucleus
13C
t-BuO2C
N
t-BuO2C
N
CO2Bn
Me
7
13C NMR (100 MHz, CDCl3)
200
180
160
140
120
100
80
60
40
20
0
f1 (ppm)

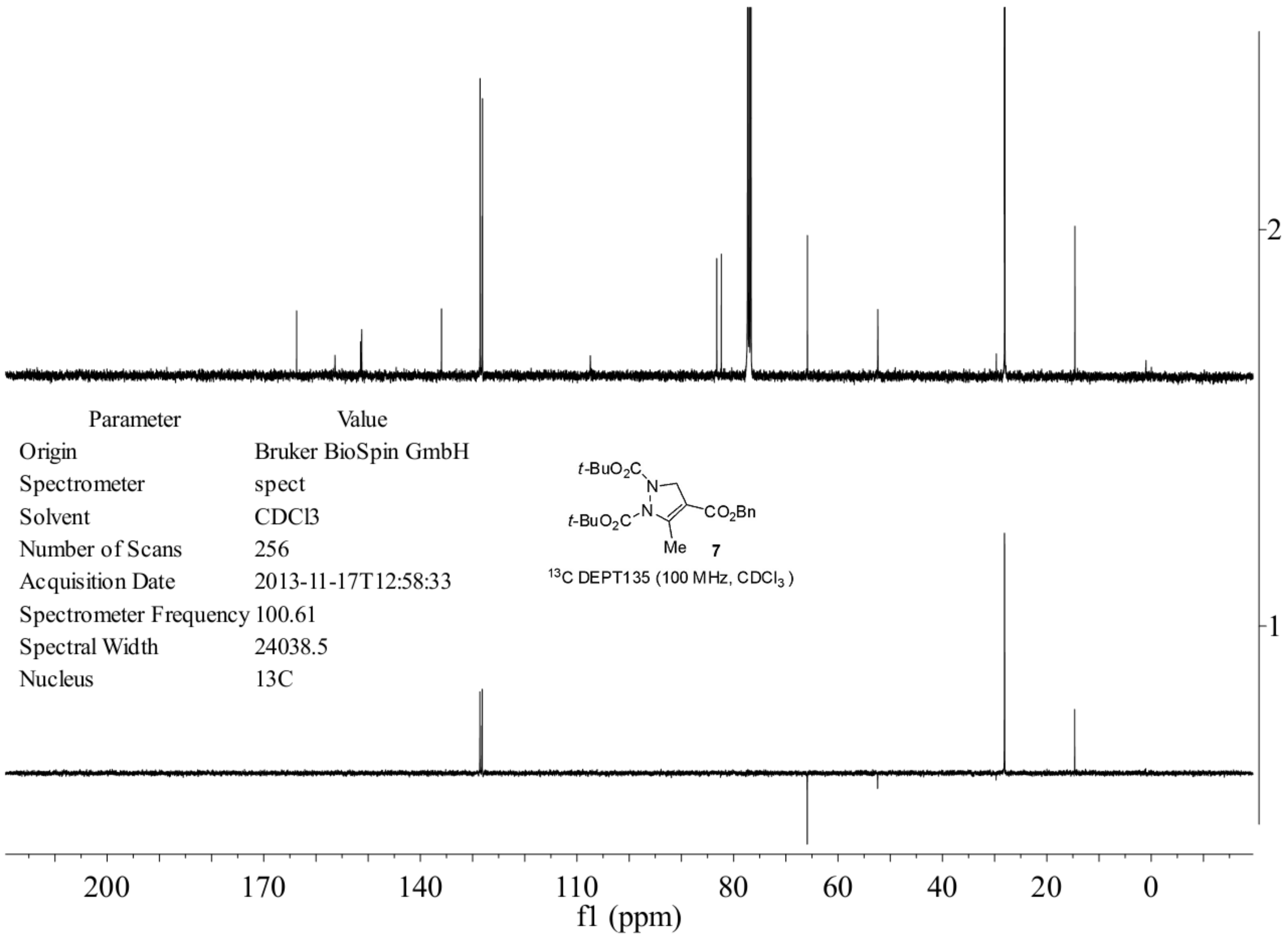

Parameter	Value
Origin	Bruker BioSpin GmbH
Spectrometer	spect
Solvent	CDCl3
Number of Scans	256
Acquisition Date	2013-11-17T12:58:33
Spectrometer Frequency	100.61
Spectral Width	24038.5
Nucleus	13C

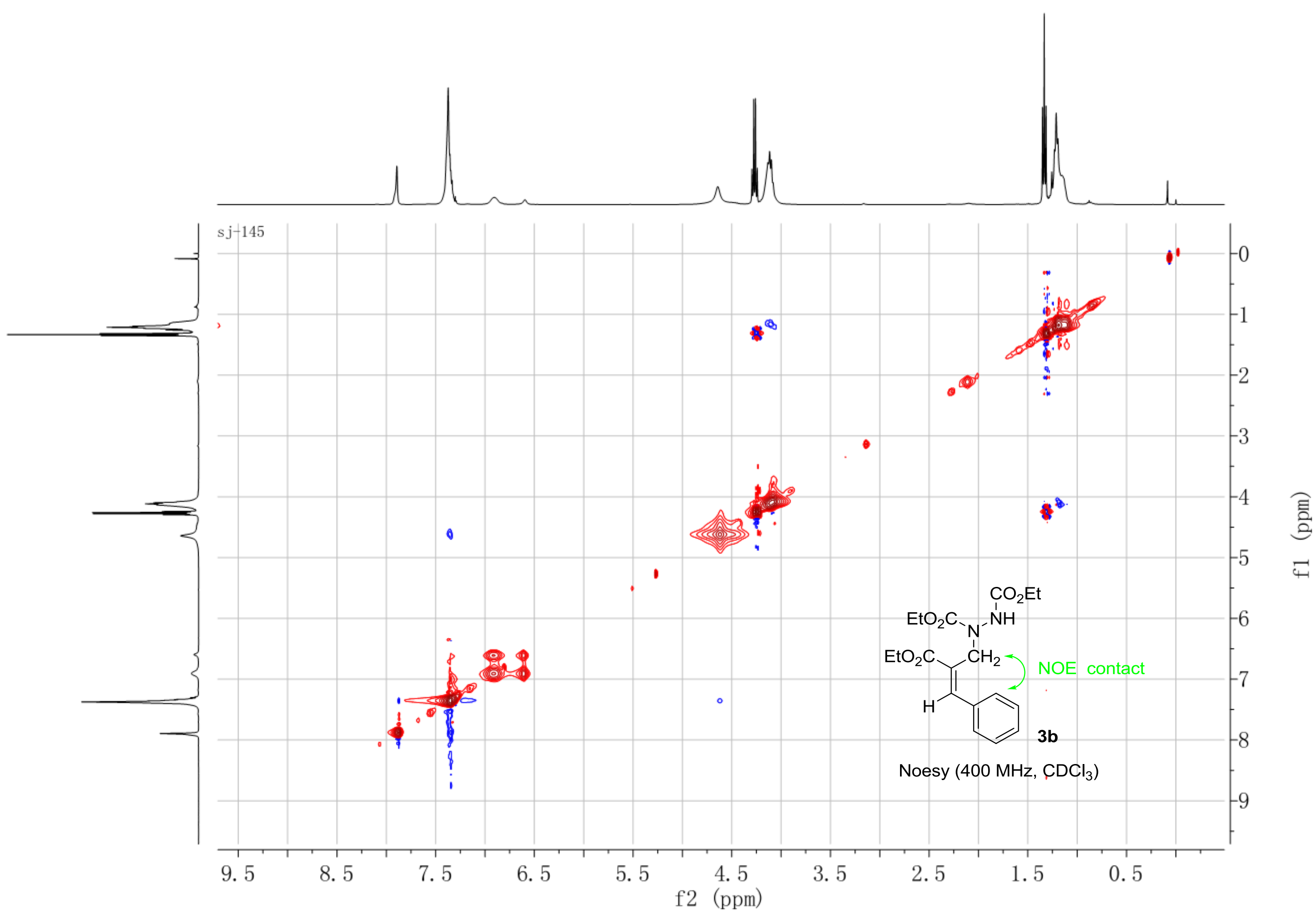
sj-145
EtO2C
CO2Et
NH
N
CH2
H
NOE contact
3b
Noesy (400 MHz, CDCl3)
9.5
8.5
7.5
6.5
5.5
4.5
3.5
2.5
1.5
0.5
f2 (ppm)
f1 (ppm)
0
1
2
3
4
5
6
7
8
9
